# NDR_A_: A single route model of response times in the reading aloud task based on discriminative learning

**DOI:** 10.1371/journal.pone.0218802

**Published:** 2019-07-31

**Authors:** Peter Hendrix, Michael Ramscar, Harald Baayen

**Affiliations:** Seminar für Sprachwissenschaft, Eberhard-Karls-Universität, Tübingen, Germany; University of Padova, ITALY

## Abstract

We present the Naive Discriminative Reading Aloud (ndr_a_) model. The ndr_a_ differs from existing models of response times in the reading aloud task in two ways. First, a single lexical architecture is responsible for both word and non-word naming. As such, the model differs from dual-route models, which consist of both a lexical route and a sub-lexical route that directly maps orthographic units onto phonological units. Second, the linguistic core of the ndr_a_ exclusively operates on the basis of the equilibrium equations for the well-established general human learning algorithm provided by the Rescorla-Wagner model. The model therefore does not posit language-specific processing mechanisms and avoids the problems of psychological and neurobiological implausibility associated with alternative computational implementations. We demonstrate that the single-route discriminative learning architecture of the ndr_a_ captures a wide range of effects documented in the experimental reading aloud literature and that the overall fit of the model is at least as good as that of state-of-the-art dual-route models.

## Introduction

Both Coltheart et al. [1] and Perry et al. [2] open what have become canonical papers in the reading literature with the observation that tremendous advances have been made in the development of reading models over the last decades. They note that early cognitive models in psychology provided mainly verbal descriptions of hypothesized cognitive architectures. These models took the form of flowchart diagrams in which boxes were used to depict mental representations, which were manipulated by cognitive processes represented as arrows that connected the various boxes (see Morton [3] for an application of box-and-arrow models to reading). Although such “verbal” models provide *descriptions* of behavioral data, their lack of specificity meant that they could only be related to the psychological and neurobiological reality of language processing at a very abstract level.

The recent development of more formal, computationally implementable models of reading [1, 2, 4, 5] has done much to address this shortcoming. As Coltheart et al. [1] remark, the development of a computational model requires a precise specification of any processes and representations that are to be implemented. As a result, computational models offer a clear improvement in specificity over informal “verbal” models of reading. Because computational models generate precise and explicit predictions, Coltheart et al. [1] continue, it is possible to evaluate them against existing behavioral data, and even falsify them through later findings. In addition, recent advances in cognitive and computational neuroscience have provided opportunities to complement this approach with even more stringent tests that investigate the neurobiological plausibility of a model’s architecture and processing mechanisms.

Since the initial implementation of the Dual-Route-Cascaded model by Coltheart et al. [1], a decade-and-a-half of recursive implementation and assessment of computational models have provided valuable insights into the successes of and the challenges for models of reading aloud. Consequently, the qualitative and quantitative performance of current state-of-the-art models of reading aloud is orders of magnitude better than that of previous generations of models.

Although current state-of-the-art models of reading aloud [1, 2, 4, 5] differ with respect to the exact mechanisms they propose, they all divide the process of reading aloud into two “routes” (i.e., sub-processes). The first route is a “lexical route”, in which mappings from orthography to phonology are mediated by lexical representations. This allows the reading of known words such as “wood” and “blood” to be simulated. The second route is a “sub-lexical” route that directly maps orthographic units onto phonological units and allows for the simulation of reading potentially unknown words, such as “snood”. As such, the general consensus seems to be that reading aloud is best modeled through a dual-route architecture. To cite Coltheart et al. [1], p. 303), “Nothing ever guarantees, of course, that any theory in any branch of science is correct. But if there is no other theory in the field that has been demonstrated through computational modeling to be both complete and sufficient, resting on laurels is a reasonable thing to do until the emergence of such a competitor—that is, the emergence of a different theory that has also been shown to be both complete and sufficient.”

In what follows, we hope to breathe new life into the single versus dual-route debate by presenting a new single-route model of response times in the reading aloud task, the Naive Discriminative Reading aloud (ndr_a_) model. The ndr_a_ is an extension of the ndr model for silent reading by Baayen et al. [6], in which both words and non-words are read through a single lexical architecture. Following the fruitful tradition described above, we evaluate the performance of the ndr_a_ model for a wide range of effects documented in the experimental word and non-word naming literature. We show that the ndr_a_ successfully captures the linear and non-linear characteristics of these effects, as well as a hitherto unobserved frequency effect for non-words. We further demonstrate that the addition of a sub-lexical route to the ndr_a_ is redundant, in that it does not improve the performance of the model.

## Existing models

In the reading aloud task, participants are presented with printed words on a computer screen and asked to pronounce these words as quickly and accurately as possible. Orthography and phonology play an important role in this process. These roles are undisputed in all current models of reading aloud, which contain both orthographic and phonological representations in one form or another. The role of semantics has been subject to a little more debate. While previous single-route models of reading aloud mapped orthography directly onto phonology, however, the consensus in more recent models is that the orthography-to-phonology mapping is mediated by semantic representations at least some of the time. Dual-route models of reading aloud have posited that while non-words are read through a direct orthography-to-phonology mapping, reading real words involves lexico-semantic representations.

Below, we discuss some of the existing state-of-the-art models of reading aloud. First, the triangle model [4, 7, 8] will be introduced. Next, we discuss the Dual-Route Cascaded Model [1]. We conclude with a description of the model of reading aloud that currently yields the best simulation results: the Connectionist Dual Process model [2, 5, 9].

### The triangle model

**Fig 1:**
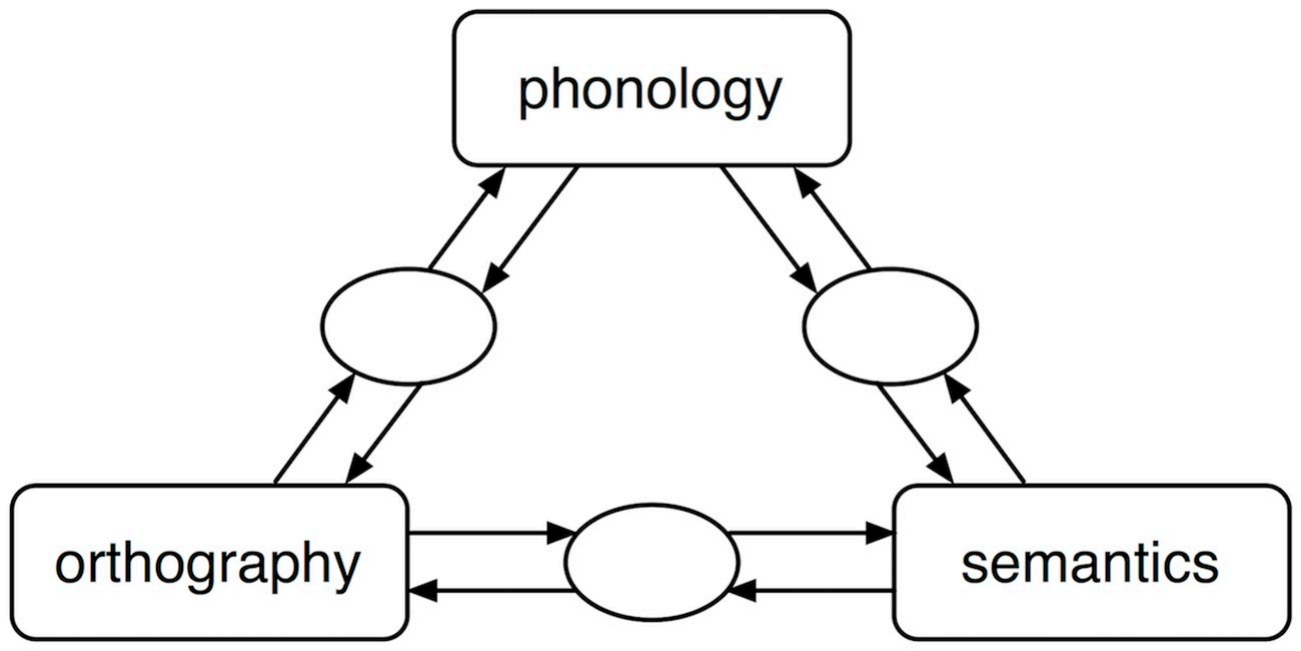
Triangle model. Basic architecture of the triangle model.

The triangle model [4, 7, 8] is a model comprising of three levels of description: orthography, phonology and semantics. Mappings between these levels of description are implemented as three-layer connectionist networks. The architecture of the model is presented in Fig 1.

In the original version of the triangle model only the direct mapping from orthography to phonology was implemented [7]. This original model therefore was a single-route model of reading aloud that directly mapped orthography onto phonology. Representations consisted of triplets of orthographic and phonological features [10] (Note, however, that these Wickelfeatures were replaced by more localist representations in Plaut et al. [8]). Associations between these orthographic and phonological units were learned through a 3-layer connectionist network.

Harm and Seidenberg [4] added semantics to the triangle model. The latest version of the model therefore has two routes from orthography to phonology. The first route is a direct mapping from orthography to phonology, as in Seidenberg and McClelland [7]. In the second route the mapping from orthography to phonology is mediated by semantic representations. The addition of a second route to the model allowed Harm and Seidenberg [4] to simulate a number of effects in the experimental literature that were not captured by previous versions of the triangle model, including effects of homophones and pseudo-homophones.

Being a connectionist model, the triangle model operates on the basis of a general learning mechanism. As such, the triangle model has increased plausibility over models that posit task-specific processing mechanisms [11]. Connectionism, however, has its own share of disadvantages. First, most connectionist networks are multi-layer networks, in which the mapping between input and output units is mediated by one or more layers of hidden units (Note, however, that Harm and Seidenberg [4] implemented a 2-layer orthography to phonology mapping that does not contain hidden units.). The contents of these hidden layer units are opaque. This reduces the transparency and interpretability of connectionist models [6].

In addition, connectionist models learn through back-propagation of error. In back-propagation learning the model output is compared to the target output. The model weights are then updated on the basis of the difference between the model output and the target output [11, 12]. As noted by Perry et al. [2], back-propagation learning has been criticized for being neurobiologically implausible [13–16].

### The Dual-Route Cascaded model

**Fig 2:**
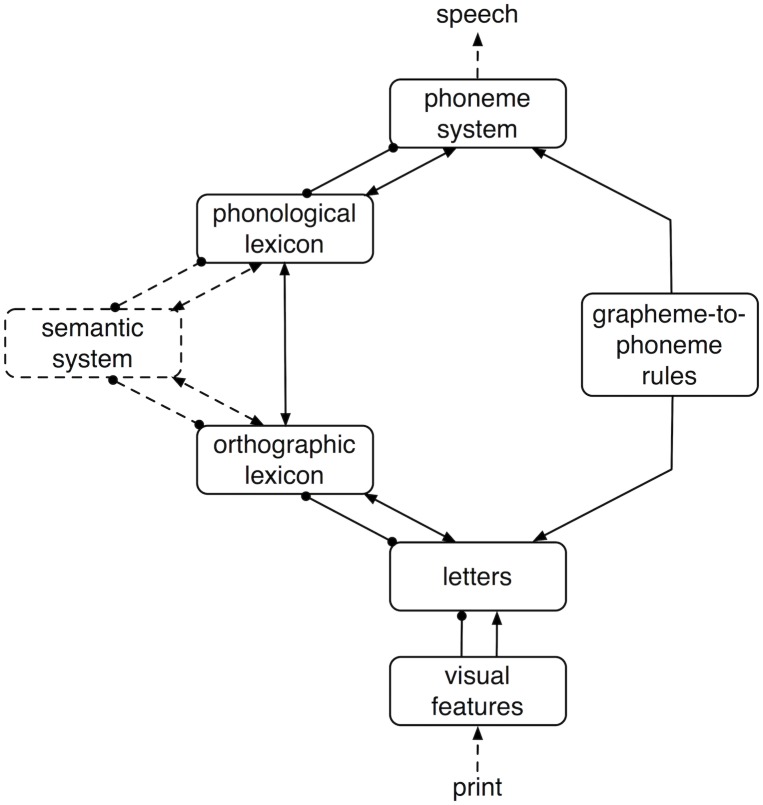
DRC model. Basic architecture of the Dual-Route Cascaded (DRC) model.

A second class of models was developed in parallel to the different versions of the triangle model. While later versions of the triangle model did include a second, lexical route [4], the Dual-Route Cascaded model (henceforth drc) [1] was the first computational implementation of a dual-route architecture. The architecture of the drc model is displayed in Fig 2.

The first stage of the model is shared by both routes and consists of an interpretation of the visual input in terms of visual features [17] that activate letter units. From this orthographic level the phonological representations required for speech can be accessed through two routes. The sub-lexical route maps letter units directly onto phonemes, whereas in the lexical route this mapping is mediated by a lexico-semantic system.

The sub-lexical route of the drc model is based on grapheme-to-phoneme conversion rules [18]. This route, which is posited to be necessary for reading non-words, operates serially in an all-or-none fashion. This sub-lexical route also underlies the successful simulation of the increased processing costs associated with words with irregular orthography to phonology mappings (i.e., mappings not predicted by the set of rules in the model). As a result of the all-or-none operation of the grapheme-to-phoneme conversion rules, however, the model has problems simulating the results of graded consistency experiments in which the number and frequency of words with consistent (i.e., the same) and inconsistent (i.e., different) orthography-to-phonology mappings is taken into account. Furthermore, the rule-based implementation of the sub-lexical route is psychologically and biologically less plausible than the learning algorithms that underlie the direct orthography to phonology mapping in other models. This concern, however, was alleviated by Pritchard et al. (2016) [19], who demonstrated that the grapheme-to-phoneme rules in the drc can be learned through implicit induction [19].

The lexical route of the drc model is based on the interactive activation model of McClelland and Rumelhart [20] and is parallel rather than serial in nature. Like the rule-system in the sub-lexical route, the interactive activation model in the lexical route of the drc model is fully hard-coded and ignores the problem of learning. Pritchard et al. (2018) [21], however, proposed a self-teaching model based on the drc model. This demonstrates that the architecture of the drc is not incompatible with learning. A remaining problem with the drc model is that it does not capture a number of important findings in the experimental literature [22, 23] (see Perry et al. [2] for a comprehensive discussion of the shortcomings of the drc model in this respect).

### The Connectionist Dual Process model

**Fig 3:**
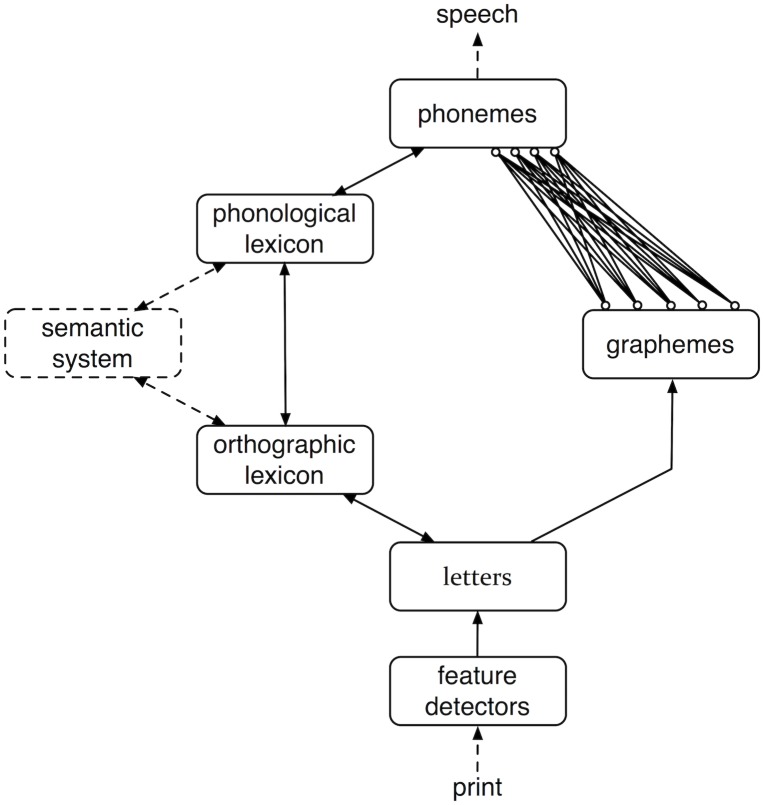
CDP model. Basic architecture of the Connectionist Dual Process (CDP) model.

The latest dual-route model is the Connectionist Dual Process model (henceforth cdp) [2, 5, 9]. Similar to the drc, the different versions of the cdp model consist of a lexical and a sub-lexical route. The basic architecture of the cdp model is presented in Fig 3.

The major advancement of the cdp over the drc model is the implementation of a two-layer associative learning network in the sub-lexical route [9, 24]. To learn the connection strengths between orthographic and phonological units the network uses the delta rule [25], which is a general algorithm for human learning [26]. As such, the implementation of the sub-lexical route of the cdp models is an important step towards a neurobiologically plausible model of reading aloud. In the most current versions of the cdp model this learning network was complemented with a graphemic buffer in the sub-lexical route [2]. This graphemic buffer organizes the orthographic information into a graphosyllabic template that uses the most frequent graphemes as representational units [5, 27].

In the original cdp model the lexical route was, in the words of Perry et al. [2], p. 297), “not implemented beyond the provision of frequency-weighted lexical phonological activation” (see Zorzi et al. [9]). The cdp+ model [2] implemented the lexical route of the drc model to overcome this problem. In doing so, however, the latest versions of the cdp model inherited the problems of interactive activation models. As such, one of the problems of the cdp+ model is that there is no learning in the lexical route (see Perry et al. [2], p. 303).

The lexical and sub-lexical routes of the cdp+ model are connected at the orthographic input and phonological output levels. On the input side of the model the visual input (i.e., the printed word) is first decoded into features with a slightly altered version of the McClelland and Rumelhart [20] feature detectors. These features are then translated into letters. At the output side of the model the information from the lexical and sub-lexical routes is integrated in a phonological decision system. Naming latencies in the cdp+ model are based on a settling criterion that terminates processing when the network is in a stable state [9].

In a comprehensive study, Perry et al. [2] demonstrated that the cdp+ model accounts for a wide range of experimental findings and shows item-level correlations with observed naming latencies that are an order of magnitude higher than those in the drc and the triangle model. We therefore consider the cdp+ model the leading model of reading aloud.

In a recent extension of the cdp+, Perry et al. [5] extended the model to bi-syllabic reading aloud. This cdp++ model correctly captures a number of experimental effects that are specifically relevant for multi-syllabic words, including effects of stress and the number of syllables. For mono-syllabic words, the cdp++ model behaves similar to the cdp+ model, with minor changes in parameter settings and the assignment of graphemes to slots in the graphemic buffer.

## The Naive Discriminative Reading Aloud model

Here, we propose the Naive Discriminative Reading Aloud (ndr_a_) model of response times in the reading aloud task. The ndr_a_ differs from existing models of reading aloud in two ways. First, the computational implementation of the ndr_a_ is entirely based on the general principles of human learning described by the Rescorla-Wagner equations [28]. These equations are similar to the delta rule that is used in the sub-lexical network of the cdp model. As such, the ndr_a_ stands in sharp contrast to the lexical route of the cdp and drc models, which are based on the interactive activation model of McClelland and Rumelhart [20]. The computational engine of the ndr_a_ also differs substantially from the connectionist networks that underlie the triangle model. It uses simple, transparent two-layer learning networks that directly map input units onto output units. In contrast to connectionist networks, these networks do not rely on the often uninterpretable hidden layer units or back-propagation of error. We provide a detailed description of the Rescorla-Wagner learning principles below [28].

Second, unlike the models discussed in the previous section, the ndr_a_ consists of a single lexical architecture. The most recent version of the triangle model and the drc and cdp models assume the use of both a lexical and a sub-lexical route in reading aloud, whereas the earlier single-route implementations of the triangle model were sub-lexical in nature. By contrast, ndr_a_ applies a single lexical mechanism in both word and non-word reading.

The architecture for word reading in the ndr_a_ is straightforward and similar to the processes underlying word reading in the lexical routes of existing models. Visual stimuli activate orthographic units. These orthographic units activate lexical representations of target words. In addition, they spread activation to lexical representations of orthographically similar words. The lexical representations of both the target word and the orthographic neighbors then activate phonological output units.

We propose that the reading of non-words occurs in a similar fashion. For non-words, however, no lexical representations exist. Therefore, instead of activating the lexical representations of both the target word and orthographically similar words, non-word orthographies only activate the lexical representations of orthographic neighbors and only these lexical representations subsequently activate phonological units.

In what follows we demonstrate that a wide range of non-word reading effects documented in the experimental literature follow straightforwardly from this simple architecture. This architecture also accounts for a novel finding, namely a non-word frequency effect in reading aloud. This non-word frequency effect suggests that the distinction between words and non-words may not be as black and white as previously thought and provides independent evidence for the involvement of lexical processes in non-word reading.

### Model architecture

**Fig 4:**
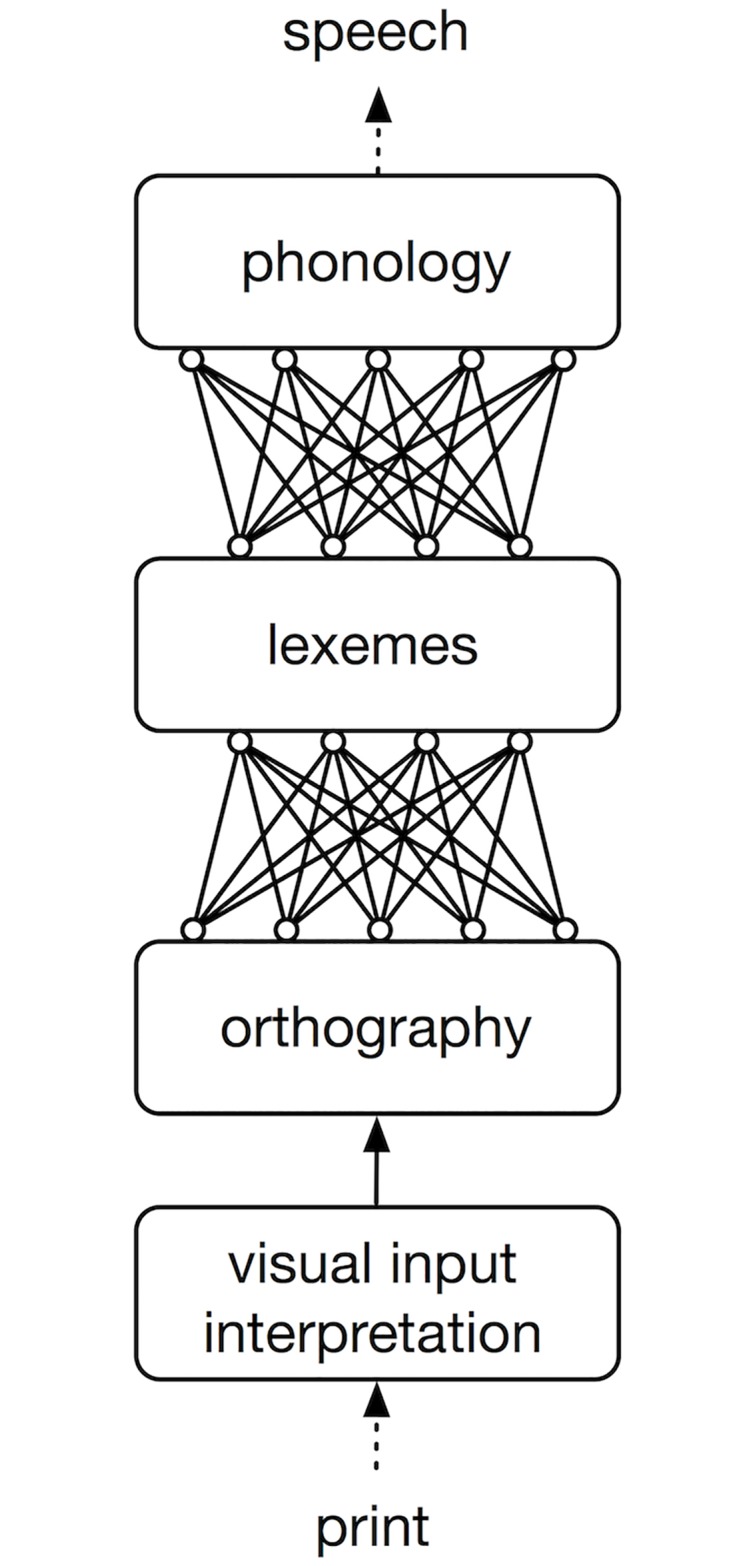
NDR_A_ model. Basic architecture of the Naive Discriminative Reading Aloud (NDR_A_) model.

The architecture of the ndr_a_ model is presented in Fig 4. The model assumes that reading aloud involves three processing stages. In the first stage, the visual input is interpreted and decoded into orthographic units. In the second stage, these orthographic units activate lexical representations in the mental lexicon that we will refer to as lexemes (i.e., lexical targets that link orthographic, phonological and semantic properties of words [29]). In the third stage these lexemes activate phonological output units. The second and third stages of the model are implemented as two-layer associative learning networks, using the Rescorla-Wagner learning rule.

### The Rescorla-Wagner model

In the Rescorla-Wagner model [28] cues are associated with outcomes. Both cues and outcomes are not position-specific. In a model of silent reading, for instance, cues may be letters or letter bigrams, whereas outcomes may be lexical representations for words. We define the presence of a cue or outcome *X* at time *t* as present(*X*, *t*) and its absence as absent(*X*, *t*). The association strength $V_{i}^{t+1}$ between outcome *O* and cue *C*_*i*_ at time *t* + 1 is given by the recurrent relation:

$$\begin{array}{c} V_{i}^{t+1}=V_{i}^{t}+\Delta V_{i}^{t}. \end{array}$$ (1)

The change in association strength $\Delta V_{i}^{t}$ is defined by Rescorla and Wagner [28] as:

$$\begin{array}{c} \Delta V_{i}^{t}=\{\begin{array}{cc} 0 & \;\text{if}\;\text{absent}\left(C_{i},t\right)\;\\ \alpha_{i}\beta_{1}(\lambda -\sum_{\text{present}\left(C_{j},t\right)\;}V_{j}) & \;\text{if}\;\text{present}\left(C_{j},t\right)\&\text{present}\left(O,t\right)\\ \alpha_{i}\beta_{2}(0-\sum_{\text{present}\left(C_{j},t\right)\;}V_{j}) & \;\text{if}\;\text{present}\left(C_{j},t\right)\&\text{present}\left(O,t\right) \end{array}. \end{array}$$ (2)

Standard values for the parameters are λ = 1, all *α*’s equal, and *β*_1_ = *β*_2_. When a cue and an outcome co-occur, the association of the cue to that outcome is strengthened. When a cue occurs without the outcome being present, the association strength decreases.

The Rescorla-Wagner model describes learning over time. The current paper, however, focuses on the adult language processing system. For simplicity, we assume that the adult system is in a relatively stable state. We therefore use the implementation of the equilibrium equations for the Rescorla-Wagner model [30] in version 0.2.18 of the ndl package for the statistical software r to estimate the connection strength (*V*_*ik*_) of cue (*C*_*i*_) to outcome (*O*_*k*_):

$$\begin{array}{c} \mathrm{Pr}\left(O_{k}|C_{i}\right)-\sum_{j=0}^{n}\mathrm{Pr}\left(C_{j}|C_{i}\right)V_{jk}=0 \end{array}$$ (3)

where Pr(*C*_*j*_|*C*_*i*_) is the conditional probability of cue *C*_*j*_ given cue *C*_*i*_, Pr(*O*_*k*_|*C*_*i*_) is the conditional probability of outcome *O*_*k*_ given cue *C*_*i*_ and *n* + 1 is the number of different cues. The estimation of the connection strength in Eq 3 is completely parameter-free and determined by the distributional properties of the training data.

The association strengths from cues to a specific outcome *O*_*k*_ are estimated separately and independently of all other outcomes. This assumption of independence is a simplification of reality similar to the independence assumption in naive Bayes classifiers that increases the computational efficiency of the model without compromising accuracy. It motivates the word naive in the name of the Naive Discriminative Reading Aloud model.

For a given input only a small set of cues is active. Denoting the set of active cues by C, the activation *a*_*k*_ of an outcome *O*_*k*_ is defined as

$$\begin{array}{c} a_{k}=\sum_{j\in C}V_{jk}. \end{array}$$ (4)

with *j* ranging over the active cues and *V*_*jk*_ being the equilibrium association strength for cue *C*_*j*_ and outcome *O*_*k*_. We add a small back-off constant (*b*, set to 0.01) to all activations. This prevents division by zero during the generation of simulated naming latencies (see below).

### Visual input interpretation

Prior to linguistic processing a decoding of the visual input is necessary. Both the drc and the cdp models use feature detection mechanisms that are similar in nature to the features detection mechanism in McClelland and Rumelhart [20]. The visual input interpretation mechanism in the ndr_a_ is a quantitative implementation of a feature decoding mechanism that is based on the idea that more complex visual patterns should take longer to decode.

We used a variant of the Manhattan city-block distance measure [31] to quantify the complexity of a letter in English. First, we constructed vector representations of the bitmaps of all 26 letters as written in black Lucida typewriter font on a white background (font size: 16). Each vector contains 400 elements representing the bit values for 20 horizontal and 20 vertical pixels. Black pixels are encoded as 1, white pixels as 0. Given the vector *B* of bit values, the complexity *C* of a given letter *i* is defined as the square root of the summed difference in pixels between that letter and the other letters *j*_1,…,26_:

$$\begin{array}{c} C_{i}=\sqrt{\sum_{j=1}^{26}\sum_{k=1}^{400}\left|B_{ik}-B_{jk}\right|} \end{array}$$ (5)

where *k*_1,2,…,400_ are the indexes of the pixels.

Eq 5 quantifies the prototypicality of the visual features of a letter. Values of *C*_*i*_ are low for letters that are similar to many other letters, such as *o* and *c*, and high for letters that are dissimilar to most other letters, such as *y* or *w*. To obtain the complexity of the visual input for a word *w* we summed over the complexities *C* of the letters *i*:

$$\begin{array}{c} {\text{Complexity}}_{w}=\sum_{i=1}^{n}C_{i} \end{array}$$ (6)

where *n* is the number of letters in the word.

The *Complexity* measure is an obvious simplification of the complex processes involved in the uptake of visual information and merely serves as an approximation of the processing costs associated with the decomposition of a visual word form into orthographic features. Given that the uptake of visual information is not part of the linguistic core of the model this approximation suffices for the current purposes. In the discussion section we briefly discuss alternative implementations of a visual input interpretation mechanism.

### Orthography to lexemes

The first part of the linguistic core of the ndr_a_ model consists of a Rescorla-Wagner network that maps orthographic units onto lexical representations. The orthographic input cues in this network are letters and letter bigrams. For instance, for the word *bear* the input units are the letters *b*, *e*, *a*, *r* and the letter bigrams *#b*, *be*, *ea*, *ar* and *r#*. Richer encodings could be used, but in the interest of parsimony we opted for the least rich encoding scheme that offered satisfactory performance.

The outcomes of the orthography to lexeme learning network are lexical representations. For the word *bear*, for instance, the outcome is the lexeme *BEAR*. In addition to the lexeme of the target word, we allowed the orthographic input units to co-activate the lexemes corresponding to other words. The orthographic word form *bear*, for instance, co-activates the lexemes *YEAR* and *FEAR*. The co-activation of orthographic neighbors predicts neighborhood and consistency/regularity effects and allows for lexical route processing of non-words. The number of co-activated words taken into consideration is a technical parameter of the model. In all simulations reported in this study this parameter was set to 20. Simulation accuracy is highly similar across a wide range of parameter settings and asymptotes for higher values.

The activation of a word’s lexeme given its orthographic representation is defined as the sum over the weights from the letters and letter bigram cues to the lexeme outcome (see Eq 4) and will henceforth be referred to as *ActLexeme*. As noted above, we add a small back-off constant (*b*, set to 0.01) to all activations to prevent division by zero when we generate simulated naming latencies.

### Lexemes to phonology

The mapping from lexical representations to phonology occurs through a similar Rescorla-Wagner learning network. This network maps lexical representations onto phonological units. As before, lexical representations are lexemes. The phonological units are demi-syllables [32]. Using the disc notation from the celex lexical database [33]), for instance, the target word *bear*, consists of two demi-syllables: *b8* and *8R*. Note that these representations are approximations of the demi-syllables used in the speech recognition literature. In our demi-syllables vowels are repeated, whereas in acoustic applications they are split at maximum intensity. Furthermore, it is again important to note that demi-syllables are merely a practically convenient approximation of the acoustic gestures necessary for speech production. We return to this issue in the discussion section.

While the activation flow in the ndr_a_ is from lexemes to demi-syllables, we trained the model with demi-syllables as input cues and lexemes as outcomes. This training regime optimizes discriminative learning, because it uses a one-to-many rather than a many-to-one mapping [34]. Training a network with demi-syllables as cues and lexemes as outcomes is necessary on independent grounds for speech perception. The training regime adopted here reflects the temporal precedence of speech perception over speech production in language acquisition.

The activation of a demi-syllable is obtained by summing over the weights on incoming connections from the active lexemes. The majority of activation spreads from the target word lexemes. We refer to this activation from the target lexeme as *a*_*t*_. Additional activation *a*_1,…,*n*_ spreads to a target demi-syllable from the lexical representations of orthographic neighbors. Given the orthographic input *bear*, for instance, the activations of lexemes of the orthographic neighbors *YEAR* and *FEAR* are 0.036 and 0.004. We weighted the contribution of co-activated lexemes to a demi-syllable for the amount of activation they received from the target word orthography (*w*_*i*_). Thus, the activation of a demi-syllable *k* is given by:

$$\begin{array}{c} {\text{ActPhon}}_{k}=w_{lex}*a_{t}+\sum_{i=1}^{n}w_{i}*a_{i} \end{array}$$ (7)

where *n* is the number of lexical neighbors taken into account (set to 20 in the current simulations). As before, we add a small back-off constant (*b* = 0.01) to all activations to prevent division by zero when generating simulated naming latencies.

The parameter *w*_*lex*_ indicates the relative weight of the activation from the target lexeme as compared to the activation from lexical neighbors and was set to 4.700 in the current simulations. As such, the activation of a demi-syllable from the target lexeme has a greater weight than the activation from the lexemes of co-activated orthographic neighbors. This is possible only if the language processing system is able to verify that the target lexeme corresponds to the orthographic input, whereas the lexemes of co-activated neighbors do not. Importantly, this assumption is not unique to the ndr_a_. Instead, it is a general assumption of discrimination learning that is necessary to evaluate if the outcome of a learning event is predicted correctly and, consequently, to update the association strengths between the cues that are present in the input and all outcomes.

The fact that the ndr_a_ performs optimally when the relative weight of the activation from the target lexeme is greater than the relative weight of the activation from the lexical neighbors suggests that while lexical neighbors spread activation to demi-syllables during initial bottom-up processing, this activation is suppressed during subsequent processing stages due to top-down verification of the activated lexemes vis-a-vis the current orthographic input. As such, the architecture of the ndr_a_ is consistent with the idea that successful processing may be characterized by a bi-directional pass of information between higher and lower level cortical representations [35]. As we demonstrate below, the bottom-up pass of information through the principles of discrimination learning captures a wide range of effects observed in naming latencies. The principles underlying the verification processes in the backward top-down information pass, by contrast, are much less well-understood. We return to this issue when discussing the pronunciation performance of the ndr_a_ model.

Two demi-syllables need to be activated for the mono-syllabic words in this study. We refer to the activation of these demi-syllables as *ActPhon_1_* and *ActPhon_2_*. The activation of two demi-syllables introduces a choice problem: one of the activated demi-syllables has to be articulated first. The more dissimilar the activations of the demi-syllables, the harder it may be to produce the right demi-syllable at the right time. A relatively high activation of the second demi-syllables, for instance, may interfere with the production of the first demi-syllable. We model the difficulty of the selection of the appropriate demi-syllable by taking the Shannon entropy [36] over the activations (transformed into probabilities *p*_1_ and *p*_2_) of the first and second demi-syllable. We refer to this measure as *H*, which is defined as:

$$\begin{array}{ccc} p_{1} & = & {\text{ActPhon}}_{1}/\left({\text{ActPhon}}_{1}+{\text{ActPhon}}_{2}\right),\\ p_{2} & = & {\text{ActPhon}}_{2}/\left({\text{ActPhon}}_{1}+{\text{ActPhon}}_{2}\right),\\ \text{H} & = & -\sum_{i=1}^{2}\left(p_{i}*\log_{2}\left(p_{i}\right)\right). \end{array}$$ (8)

### Simulating naming latencies

Together, the measures *Complexity*, *ActLexeme*, *ActPhon_1_*, *ActPhon_2_* and *H* describe the total amount of bottom up support for the target pronunciation. Lexeme activations for non-words are, by definition, not available. For non-words, *ActLexeme* was therefore set to 0.01 (0 plus the back-off constant *b* that was added to all lexeme activations for words. Total activation units in the ndr_a_ are modeled through a multiplicative integration of these measures:

$$\begin{array}{c} \begin{array}{c} Act=\frac{{\text{Complexity}}^{w_{1}}}{{\text{ActLexeme}}^{w_{2}}*{\text{ActPhon}}_{1}^{w_{3}}*{\text{ActPhon}}_{2}^{w_{4}}*{\text{H}}^{w_{5}}} \end{array} \end{array}$$ (9)

where *w*_1,…,5_ are weight parameters that establish the relative contribution of each source of information.

Model parameters were chosen to optimize the quantitative and qualitative performance of the model. For the current simulations, we use the following parameter settings: *w*_1_ = 1.270, *w*_2_ = 0.200, *w*_3_ = 0.050, *w*_4_ = 0.098 and *w*_5_ = 0.152. Parameter settings are identical in all simulations reported in this study.

We convert activation units to simulated reaction times through a simple linear transformation:

$$\begin{array}{c} \begin{array}{c} RT\propto w_{6}*Act+w_{7}. \end{array} \end{array}$$ (10)

For all simulations reported below, we set *w*_6_ to 0.055 and *w*_7_ to 450. Including the two technical parameters described earlier (i.e., the back-off parameter that prevents division by zero (0.01) and the number of co-activated neighbors taken into consideration (20)), as well as the parameter for the relative importance of demi-syllable activations from the target word lexeme and the lexemes of lexical neighbors (4.700), the ndr_a_ model thus has a total of 10 free parameters.

### Generating pronunciations

The processes by which responses are learned are relatively well understood and large-scale linguistic corpora provide us with realistic input to these processes. As demonstrated through the simulations reported in this paper, the discriminative learning algorithm that underlies the ndr_a_ provides a precise and powerful explanation of these bottom-up processes and their behavioral manifestations in observed naming latencies. What the discrimination learning core of the ndr_a_ model does not do, however, is generate actual pronunciations.

The selection of the appropriate target response is perhaps best thought of as a response conflict resolution task. In the words of Ramscar et al. [37], “Response conflict will arise whenever the requirements in a specific task conflict with an equally or more strongly learned pattern of responding that is prompted by the same context. To successfully resolve this conflict, an individual must be able to effectively override the biased response in favor of a less well-learned (or less well-primed) response that is more appropriate to the context” (see also [38, 39]). In the ndr_a_ model a response conflict arises whenever a non-target demi-syllable receives a higher activation than the target demi-syllables.

Response conflicts are typically resolved by a top-down verification mechanism that integrates the activated responses with the context of the current task. Dell [40] and Levelt et al. [41], for instance, proposed such top-down verification mechanisms in their models of language production. In reading aloud, the task of a top-down checking mechanism is to find out which of the activated phonological units should be pronounced given the visual presentation of a word or non-word. What we suggest, therefore, is that there is a functional separation between the bottom-up linguistic support for phonological units that arises in the discrimination learning networks that form the linguistic core of the ndr_a_ model and the top-down verification mechanism that evaluates the appropriateness of these phonological units given the task of naming the presented word or non-word.

There is a wealth of evidence in both the neuroscience and reading literatures to support a functional separation of this kind (see e.g. [38]). In particular, the anterior cingulate cortex (acc) and the pre-frontal cortex (pfc) seem to play an important role in resolving competition between different potential responses (see e.g. [42]). Functionally, the acc appears to serve as a detector, monitoring conflict between candidate responses and activating areas in the pfc that facilitate the selection of the appropriate target response when conflicts arise.

The Stroop task, in which subjects have to name the text color of an orthographic representation of a conflicting color word neatly illustrates the dynamics of this process in a reading task. When the word “blue” is printed in red, the correct response is “red”. In literate adults, however, the orthographic activation of “blue” interferes with the correct response. In the Stroop task, activation in the pfc, and in particular the left inferior frontal gyrus has been shown to reflect the effort required to produce the text color “red” rather than the strongly activated competitor “blue” [43]. As noted by Novick et al. [39], the pfc plays a functionally similar role when response conflict arises in a range of more straightforward lexical tasks, including lexical decision (see e.g. [44]), verb generation [45], picture naming [46], and phonological and semantic judgment tasks [47], as well as when interpretative conflicts arise during normal reading [39].

As noted above, the processes by which responses are learned are relatively well understood. By contrast, a lot of uncertainty remains about how exactly the top-down verification processes in the pre-frontal cortex that select the appropriate response from a set of activated potential responses work. The objective of the present study is to demonstrate that the discrimination learning networks in the ndr_a_ model capture important aspects of the bottom-up learning processes and their manifestations in observed naming latencies, much like the original ndr model captures a wide range of reaction time effects in the lexical decision task. Given the increased prominence of the actual response in the reading aloud task as compared to the lexical decision task, however, it is useful to demonstrate that the architecture of the ndr_a_ model is compatible with a top-down checking mechanism that generates concrete and plausible pronunciations. At the end of the simulations section below, we therefore present a crude implementation of a verification mechanism, as well as the word and non-word naming performance of the ndr_a_ model when such a checking mechanism is added on top of the discrimination learning networks of the model.

## Simulations

### Training and test data

For all simulations described below we trained the orthography-to-lexeme network of the ndr_a_ model on an input lexicon that consisted of all words with a frequency of at least 20, 000 in the the Google 1*T* n-gram corpus [48]. The resulting orthography-to-lexeme network consists of 754 unique letters and letter bigrams and 217, 170 unique lexemes. The training data for the lexeme-to-phonology network consisted of a set of 3, 198 mono-morphemic, mono-syllabic words and their unigram frequencies in the Google 1*T* n-gram corpus [48]. The set of 3, 198 words was constructed in the following manner. Following the simulation of Baayen et al. [6] for silent reading, we restricted the simulations for the word naming latencies to mono-morphemic, mono-syllabic words that can be used as nouns. First, we therefore extracted all mono-morphemic, mono-syllabic words that can be used as nouns from the celex lexical database [33]. We excluded 1 and 2 letter words (1.64% of all monosyllabic word types in the celex lexical database) from the training data to prevent biasing the results in favor of a coding scheme that adopts bigram representations at the orthographic level, such as the one used here. This resulted in a set of 3, 146 words. To this set of words we added 52 words that were necessary for the correct pronunciation of words or non-words in our test data (i.e., words that contained an orthography-to-phonology mapping that was present in the test data, but not in the set of 3, 146 words extracted from celex). The trained phonology-to-lexeme network consists of 1, 228 unique demi-syllables and 3, 198 unique lexemes.

For the word naming simulations, we used a data set consisting of the 2, 510 mono-morphemic mono-syllabic words present in our training data that can be used as nouns and for which naming latencies are available in the elp [49]. Prior to analysis we inverse transformed (−1000/RT) the observed naming latencies to remove a rightward skew from the naming latency distribution. In addition, to allow for a comparison of effect sizes, we standardized observed and simulated latencies by converting them to *z*-scores.

No large-scale database of naming latencies is available for non-words. We therefore extracted a set of non-words from the arc non-word database [50]. We restricted the range for non-word length to that observed in our set of real words and extracted non-words with orthographically existing onsets and bodies only. Furthermore, we restricted the non-words to the words for which both demi-syllables existed in our training lexicon. This resulted in a non-word data set that consisted of 1, 784 non-words: 876 standard non-words and 908 pseudo-homophones.

We looked at the effects of 16 linguistic predictors, related to the length, neighborhood characteristics, orthography-to-phonology consistency, frequency, and semantics of a word or non-word. Predictor values were extracted from the elp and the *english* data set in the *languageR* package for r [51]. Whenever necessary, a more detailed description of each predictor will be provided prior to the description of the results for that predictor.

### Model evaluation

Model evaluation in cognitive psychology typically involves comparing a model’s performance to both observed naming latencies and alternative model architectures. The observed data used in our simulations are the elp naming latencies for the set of 2, 510 mono-morphemic nouns described above. We compare the ndr_a_ model not only to the observed naming latencies, but also to the drc [1], cdp+ [2], and cdp++ [5] models.

The ndr_a_ model is implemented in the *NDR_A_* package for r [52]. We simulated naming latencies for the 2, 510 words under investigation through this package. Simulated naming latencies for the other models were obtained using version 1.2.3 of the drc model and the implementation of the cdp+ and cdp++ models available at http://ccnl.psy.unipd.it/CDP.html.

### Simulation approach

The adequacy of a model can be investigated by comparing its predictions against observed data. This comparison typically focuses on two levels of description. The first level is the overall fit of the model to a set of observed data. Typically, this overall fit is gauged through the *regression approach*. In the regression approach item-level correlations between simulated and observed naming latencies are compared for a large-scale database of words. Here, we follow this approach by looking at the item-level correlations between the elp naming latencies and the latencies simulated by the ndr_a_, drc, cdp+, and cdp++ models. To further probe the overall performance of these models we furthermore conduct a regression analysis on the principal components extracted from the multidimensional space described by all predictors in our simulations. This provides more insight into how well each model captures the overall structure in the observed data.

The second level at which the performance of a model can be investigated concerns the effects of individual predictors on observed naming latencies. The approach that is most typically used to do this is the *factorial approach*. In the factorial approach patterns of results related to predictors are simulated on an experiment-by-experiment basis (for an application, see, e.g. [1, 2]). As noted by Adelman and Brown [53], however, there are a number of problems with the factorial approach.

First, the data gathered in single experiments tend to provide an incomplete picture of the effect of a predictor. The experimental data that models of reading aloud are assessed on are often acquired in experiments with a limited number of carefully selected items and under different experimental conditions. As a result, optimizing the parameter set of a model on the basis of individual experiments may lead to local over-fitting. The model then becomes overly sensitive to the potentially idiosyncratic experimental conditions, item lists and predictor combinations in individual experiments, which comes with the cost of a suboptimal overall model fit (see e.g., Seidenberg and Plaut [54]).

Second, modeling on an experiment-by-experiment basis makes it hard to compare the relative effect sizes of different predictors. Due to variations in item lists, experimental conditions and participant populations, the effect sizes for a given predictor can vary substantially between experiments. Given this variance in the effect sizes for a *given* predictor, it is hard to compare effects sizes *between* predictors in the factorial approach.

Third, a large number of experiments are based on factorial contrasts. This leads to a potential distortion of non-linear patterns of results that can range from a simplification of a non-linear effect to masking a predictor effect completely. Applying a median-split dichotomization to a predictor that has a U-shaped effect on response latencies, for instance, would yield a null effect.

To overcome these problems with the factorial approach we adopt a different simulation philosophy. Instead of looking at predictor effects on an experiment-by-experiment basis we will investigate the effects of all relevant predictors in the naming latencies for the set of 2, 510 words in the elp. All of the elp naming latencies were obtained in the same task, under very similar experimental conditions and for a homogenous participant population. The presence of an effect in the elp is a clear indication that computational models should account for this effect. In addition, using elp naming latencies allows for a comparison of effect sizes between predictors. Furthermore, it allows us to look at the effects of different predictors in a setting where parameters should not be allowed to vary. Finally, because we have access to naming latencies for individual items we can get away from the dichotomization of numeric predictors and start investigating non-linear predictor effects.

### Predictor simulations

We investigate a large number of effects that have been documented in the experimental reading aloud literature. For each effect under investigation, we first verify whether an effect was present in the elp naming latencies. For a large majority of the effects documented in the literature this is indeed the case. Whenever an effect is not present in the elp naming latencies we explicitly mention its absence. For those effects that are present in the elp naming latencies we proceed with an analysis of the effect for the simulated latencies of the ndr_a_, drc, cdp+, and cdp++ models.

To investigate the effects of predictors we use the implementation of generalized additive models (henceforth gams) [55] provided by the r package *mgcv* [56]. GAMs are an extension of generalized linear models that allow for the modeling of non-linearities. For each predictor effect we fitted both a linear and a non-linear gam. The linear gam is mathematically equivalent to a simple linear regression model. This linear model provides a conventional assessment of the presence or absence of predictor effects. In addition it provides an effect size measure that allows for the comparison of the relative magnitude of effects of different predictors. To allow for such a comparison, we scaled all predictors prior to analysis.

The non-linear gams allow us to capture non-linearities. The smooth functions in gams do not presuppose particular non-linear structures and can therefore model a wide range of predictor-related non-linearities. Furthermore, tensor products allow us to model two-dimensional non-linear interactions between numerical predictors. As a result, we do not have to dichotomize predictors even when inspecting interaction effects. We allowed all predictor smooths to describe up to 6th order non-linearities (*k* = 6) and did not impose any restrictions on tensor products. We removed predictor values further than 3 standard deviations from the predictor mean in all non-linear gams to prevent smooth estimates from being overly influenced by extreme predictor values. To establish the significance of tensor product interactions, we compared the aic score [57] of a tensor product gam to that of a gam with additive non-linear effects of both predictors (i.e., separate predictor smooths). Unless explicitly stated otherwise, we considered interactions only when the aic score of the tensor product gam was significantly lower than that of a gam with an additive non-linear effect of both predictors.

Many of the predictors under investigation are strongly correlated. As a result introducing a model term to or removing it from a model that contains all predictors could have a strong effect on the effects of the other terms in the model. To side-step this problem of multicollinearity we decided to fit separate models for each predictor. Fitting separate models for each predictor comes at the cost of masking potential effects of covariates. We addressed this problem in two ways. First, we simulated only effects that have been documented in experimental studies with carefully controlled item lists. Nonetheless, it is important to note that the reported effect of individual predictors may be confounded the values of other predictors that are not entered into the models for the individual predictors. Second, to ensure that our model captures the joint effects of the predictors we conducted a principal components regression analysis for both observed and simulated latencies on the multidimensional input space described by all 16 predictors. The principal components analysis establishes to what extent the model correctly captures the effects of a predictor once the effects of the other predictors have been taken into account.

For word naming we fitted models to the observed naming latencies, as well as to the simulated naming latencies for the ndr_a_ and cdp+ models. As mentioned earlier, no large-scale database of non-word naming latencies exists. To simulate the non-word effects documented in the literature we therefore could not compare our model to observed naming latencies. We did, however, have the possibility of comparing non-word naming performance in the ndr_a_ and cdp+ models. This allows us to establish whether or not the single-route architecture of the ndr_a_ model captures the experimental effects of non-word naming that are successfully simulated by the drc, cdp+, and cdp++ models. Furthermore, it allows us to identify whether and where predictions for non-word naming differ between the ndr_a_ and the other models. These differences describe explicit test-cases for the performance of the models that can be addressed in future non-word naming experiments.

## Simulation results

Below, we present the results of five types of simulations. First, we investigate the effects of individual predictors describing the length, neighborhood density, orthography-to-phonology consistency, frequency, and semantic properties of words and non-words, as well as the relevant interactions between these predictors. Second, we evaluate the overall performance of the ndr_a_, the drc, the cdp+, and the cdp++ through item-level correlations, as well as a principal components analysis. Third, we investigate whether or not the addition of a sub-lexical route improves the performance of the ndr_a_ model. Fourth, we discuss a hitherto unobserved effect of frequency in non-word naming. Fifth, we describe the pronunciation performance of the ndr_a_ when we add a crude implementation of a top-down verification mechanism on top the discrimination learning core of the model.

### Non-word naming disadvantage

Before we turn to the discussion of predictor-specific effects, there is an overall difference between word and non-word naming that requires attention. Several studies have documented that words are named faster than non-words [58–60]. All models correctly predict this effect (ndr_a_: *t* = −60.999, *β* = −1.383; drc: *t* = −225.820, *β* = −1.949; cdp+: *t* = −73.590, *β* = −1.515; cdp++: *t* = −72.572, *β* = −1.505. There is, however, a large difference in the relative magnitude of the predicted effects. The drc model predicts the largest difference between naming latencies for words and naming latencies for non-words. On average, non-words are named 85% slower than words (138 vs 74 cycles). In the cdp+ and cdp++ models the non-word naming disadvantage is reduced to 57% (159 vs 101 cycles) and 64% (129 vs 79 cycles), respectively. The difference between word and non-word naming latencies is smallest in the ndr_a_. The mean naming latency for non-words is 23% longer than the mean naming latency for words (781 vs 635 ms).

Although a direct comparison to the effect size in observed data is not possible due to the absence of a large-scale database of non-word naming latencies, we compared the processing disadvantage for non-words predicted by the models to that observed in the studies of McCann and Besner [58] and Ziegler et al. [60]. The average naming latency for words in McCann and Besner [58] was 454 ms, whereas that for non-words was 579 ms. The processing disadvantage for non-words in this study was therefore 29%. In Ziegler et al. [60], average naming latencies across eight conditions were 611 ms for non-words and 521 ms for words, for a non-word processing disadvantage of only 17%. These data suggest that the drc, cdp+, and cdp++ models overestimate the processing costs for non-words, while the ndr_a_ provides a more reasonable estimate.

We should note, however, that the size of the non-word naming disadvantage in the ndr_a_ depends to a large extent on the settings of the parameters involved in the linear transformation from activation units to simulated reaction times (*w*_6_, *w*_7_). Parameter *w*_7_, in particular, captures the processes the are present in response times, but that are outside of the scope of the discrimination learning core of the model. Such processes may include, but are not limited to, response selection, lack of sensitivity of the voice key to low acoustic energy during pronunciation onset [61], and participant fatigue (see [1], p. 221). The inclusion of a similar parameter in the drc, cdp+, and cdp++ models would results in more accurate estimates of size of the non-word naming disadvantage in these models.

### Length effects

#### Word length

**Fig 5:**
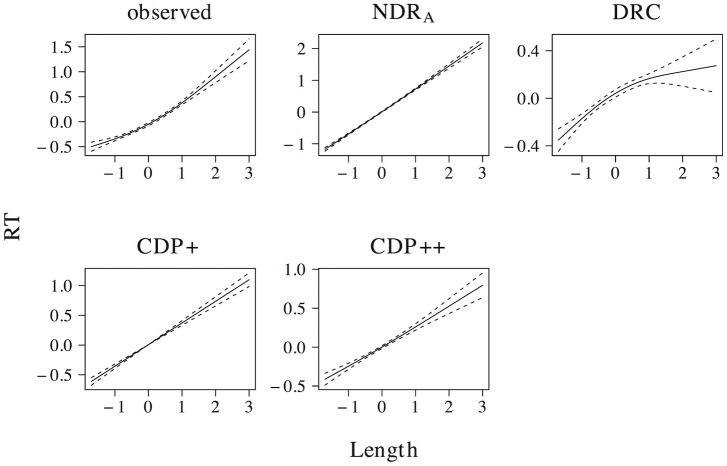
Length. The effect of length in word naming.

The effect of word length on naming latencies has been documented in a large number of studies, with longer naming latencies for words that consist of more letters (see e.g. [59, 61–67]). This length effect is present in the elp naming latencies (*t* = 20.076, *β* = 0.372, as well as in all models (ndr_a_: *t* = 51.917, *β* = 0.718; drc: *t* = 8.015, *β* = 0.158; cdp+: *t* = 20.099, *β* = 0.372; cdp++: *t* = 13.230, *β* = 0.255). The results of a non-linear model are presented in Fig 5 and indicate that this effect is linear or near-linear for the observed naming latencies, as well as for the latencies simulated by the ndr_a_, cdp+ and cdp++ models. For the naming latencies simulated by the drc model there is some indication that the effect incorrectly levels off for long words, although the confidence interval for high predictor values is large.

The effect size of the length effect is larger in the ndr_a_ model than in the observed data. The length effect in the ndr_a_ model is primarily driven by the complexity of the visual input. In all reported simulations, the visual complexity parameter is set to 1.270. The overall fit of the data, however, is quite robust to changes in this parameter setting (e.g., overall data fit: *r* ≥ 0.48 for parameter values between 0.450 and 2.902). There are two reasons we decided to use the current parameter setting. First, we believe that the overall fit of the model should be optimal. This is the case for the current parameter settings. Second, because the model operates under noise-free conditions, the effect sizes in the ndr_a_ tend to be somewhat larger than those in the observed data. As we will show in the overall model fit section, the effect size of length in the current simulations is of the correct relative magnitude compared to the effect sizes of the other predictors.

In addition to a length effect for words, a length effect for non-words has been observed as well. Non-word naming latencies increase linearly for each additional letter [59, 60]. All models capture the effect of length in non-word naming (ndr_a_: *t* = 160.667, *β* = 0.968; drc: *t* = 39.347, *β* = 0.683; cdp+: *t* = 21.342, *β* = 0.452; cdp++: *t* = 14.225, *β* = 0.320). The results of non-linear models revealed that all models predict a linear or near-linear length effect for non-words. Furthermore, consistent with the experimental findings of Weekes [59] and Ziegler [60], all models predict a larger effect size for length in non-word naming than in word naming (ndr_a_: Δ*β* = 0.250; drc: Δ*β* = 0.525; cdp+: Δ*β* = 0.080; cdp++: Δ*β* = 0.065). The relative magnitude of the length effect for non-words as compared to that for words is much larger in the drc ($\frac{\beta_{nw}}{\beta_{w}}=4.323$) than in the other models (ndr_a_: $\frac{\beta_{nw}}{\beta_{w}}=1.348$; cdp+: $\frac{\beta_{nw}}{\beta_{w}}=1.215$; cdp++: $\frac{\beta_{nw}}{\beta_{w}}=1.255$).

In addition to the effects of word length reported above, Weekes [59] also reported an interaction of length with frequency, with a stronger length effect for low frequency words. In a reanalysis of the Weekes [59] data, however, Perry et al. [2] demonstrated that this interaction was not significant. For the current set of observed naming latencies the interaction was not supported either: a model with additive non-linear terms of frequency and length resulted in a lower aic score than a gam with a tensor product of frequency and length.

### Neighborhood effects

#### Orthographic neighborhood size

Although the unique variance accounted for by neighborhood measures is small [68], these effects have played a central role in the assessment of models of reading aloud. The experimental naming literature has consistently documented that words with many orthographic neighbors are processed faster than words with fewer neighbors [69–73]. In interactive activation models, however, the inhibitory links between lexical items lead to more competition for words with many orthographic neighbors. As a result, the drc model, which uses the interactive activation model of McClelland and Rumelhart [20] as its lexical route, could only model the effect of orthographic neighborhood density with altered parameter settings. Although the cdp+ and cdp++ models use the same interactive activation architecture for its lexical route, these models capture the orthographic neighborhood density effect, presumably through their sub-lexical route. Nonetheless, the authors acknowledge that the interactive activation model in their lexical route may have inherent problems with neighborhood density effects and that there may be better alternatives for the lexical route of the cdp+ and cdp++ models (see [2], p. 303)).

The ndr_a_ model predicts orthographic neighborhood density facilitation as a consequence of the co-activation of orthographically similar words. Each co-activated orthographic neighbor activates its lexeme, from which in turn activation spreads to the corresponding demi-syllables. The target word *band*, for instance, co-activates the lexical representations of words like *bank*, *bang* and *ban*, which spread activation to the target demi-syllable *b*{. In addition, *band* co-activates *land*, *hand* and *sand*, which spread activation to the target demi-syllable {*nd*. The more orthographic neighbors a word has, the more activation will spread from co-activated lexemes to the target demi-syllables and the faster a word will be named.

**Fig 6:**
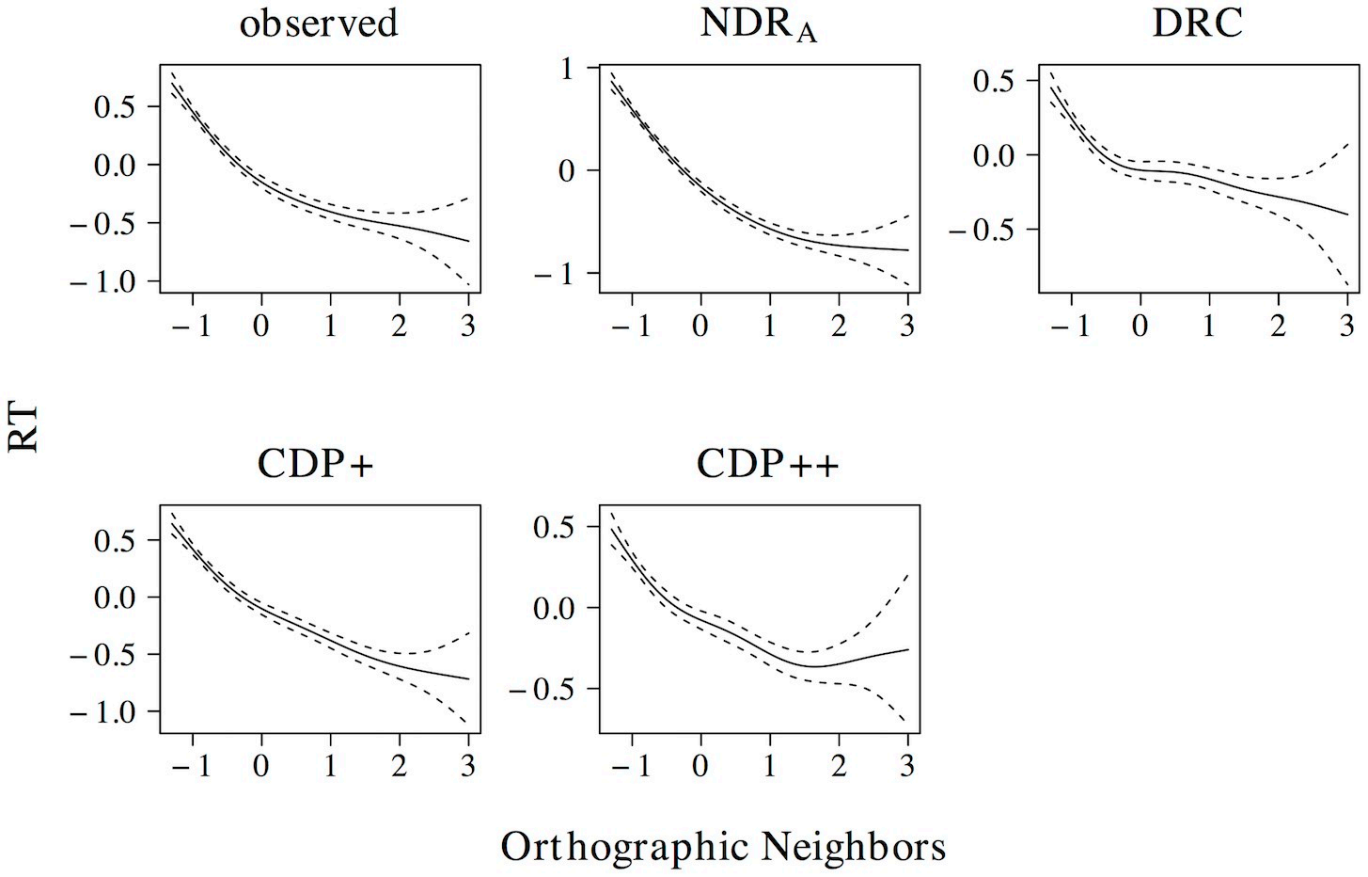
Orthographic neighborhood density. The effect of orthographic neighborhood density in word naming.

A linear model on the elp naming latencies shows the predicted facilitatory effect of orthographic neighborhood density (*t* = −19.131, *β* = −0.357). The ndr_a_ captures this linear effect of orthographic neighborhood density (*t* = −27.639, *β* = −0.483), as do the drc (*t* = −9.452, *β* = −0.185), the cdp+ (*t* = −19.229, *β* = −0.358), and the cdp++ (*t* = −12.530, *β* = −0.243). The non-linear effect of orthographic neighborhood density is shown in Fig 6. The observed data show a quadratic curve, with a more prominent facilitatory effect of orthographic neighborhood density for low predictor values. All models correctly capture the quadratic effect of orthographic neighborhood density.

In addition to an effect of orthographic neighborhood density in word naming, an effect of this measure in non-word naming has also been documented [71]. As for real words, the effect is facilitatory in nature, with faster naming latencies for non-words with many orthographic neighbors as compared to non-words with few orthographic neighbors. A linear model on the simulated naming latencies shows that all models correctly simulate this effect (ndr_a_: *t* = −30.188, *β* = −0.577; drc: *t* = −14.633, *β* = −0.325; cdp+: *t* = −14.056, *β* = −0.314; cdp++: *t* = −12.421, *β* = −0.280). Non-linear models revealed that the drc predicts a near-linear of orthographic neighborhood density on non-word naming, whereas the ndr_a_, cdp+, and cdp++ predict a quadratic non-linearity that is similar to the observed effect of orthographic neighborhood density in word naming.

#### Phonological and body neighborhood size

The effect of orthographic neighborhood density is not the only neighborhood density effect that has been documented. As noted by Perry et al. [2], several studies have argued that phonological neighborhood density [74] or body neighborhood density [60, 75, 76] may be more adequate measures of neighborhood density effects in reading aloud. The linear effect of phonological neighborhood density (observed: *t* = −12.768, *β* = −0.247; ndr_a_: *t* = −18.761, *β* = −0.350; drc: *t* = −5.327, *β* = −0.106; cdp+: *t* = −15.304, *β* = −0.292; cdp++: *t* = −10.355, *β* = −0.202), as well as that of body neighborhood density (observed: *t* = −5.897, *β* = −0.117; ndr_a_: *t* = −4.648, *β* = −0.092; drc: *t* = −16.471, *β* = −0.313; cdp+: *t* = −14.112, *β* = −0.271; cdp++: *t* = −10.700, *β* = −0.209) are captured by all models. The ndr_a_ model somewhat underestimates the magnitude of the body neighborhood density effect relative to that of the orthographic and phonological neighborhood density effects. By contrast, the drc, the cdp+ and the cdp++ overestimate the effect of body neighborhood density.

**Fig 7:**
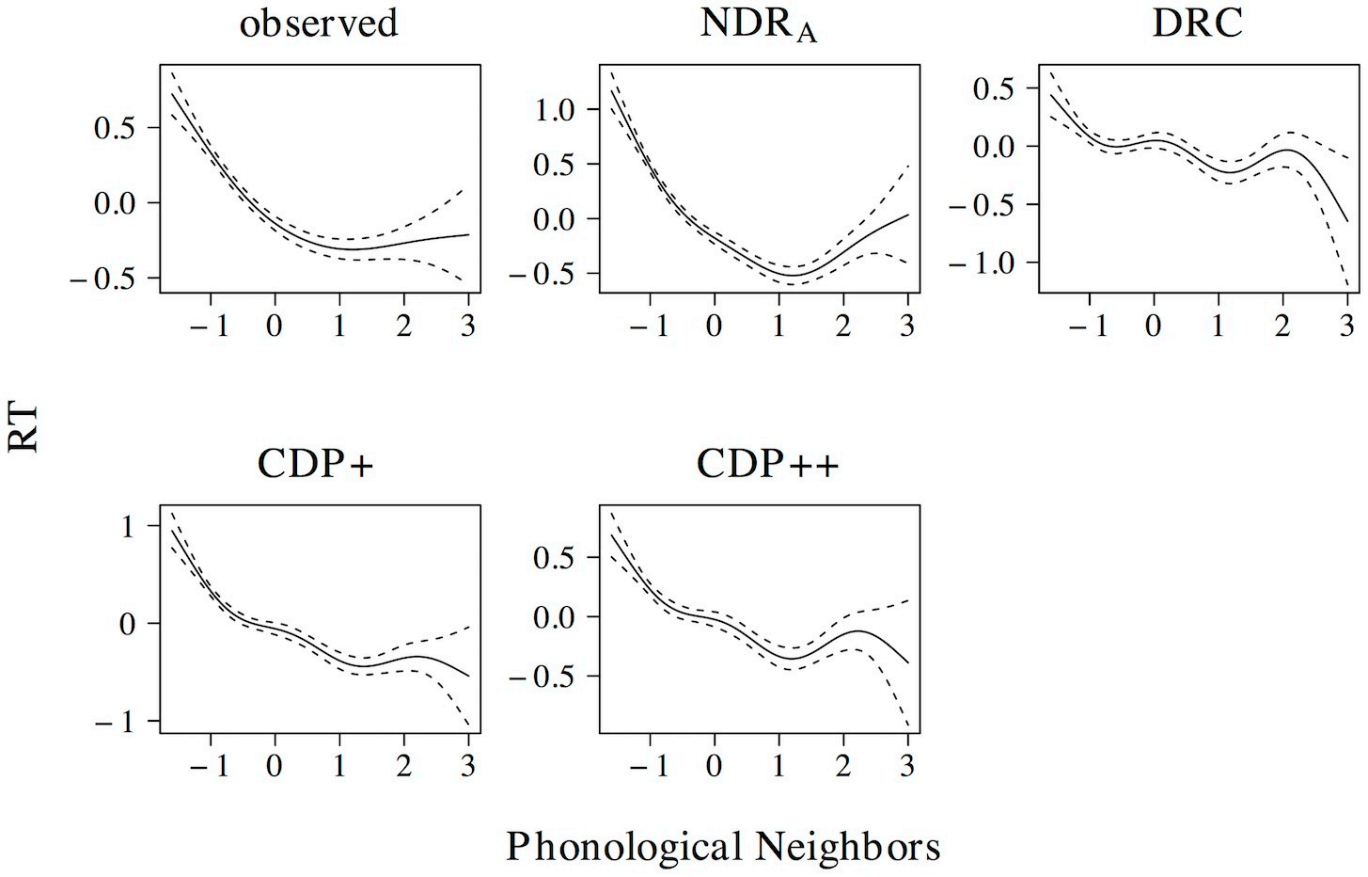
Phonological neighborhood density. The effect of phonological neighborhood density in word naming.

The non-linear effect of phonological neighborhood density is presented in Fig 7. The observed naming latencies reveal a quadratic curve that levels off for high predictor values. The ndr_a_, cdp+ and cdp++ correctly capture the overall nature of this effect, although the simulated effect in the ndr_a_ more closely resembles the observed effect as compared to the simulated effects in the cdp+ and cdp++ models. The drc fails to capture the non-linear nature of the effect of phonological neighborhood density and instead predicts a wriggly effect that is more uniform across the predictor range.

**Fig 8:**
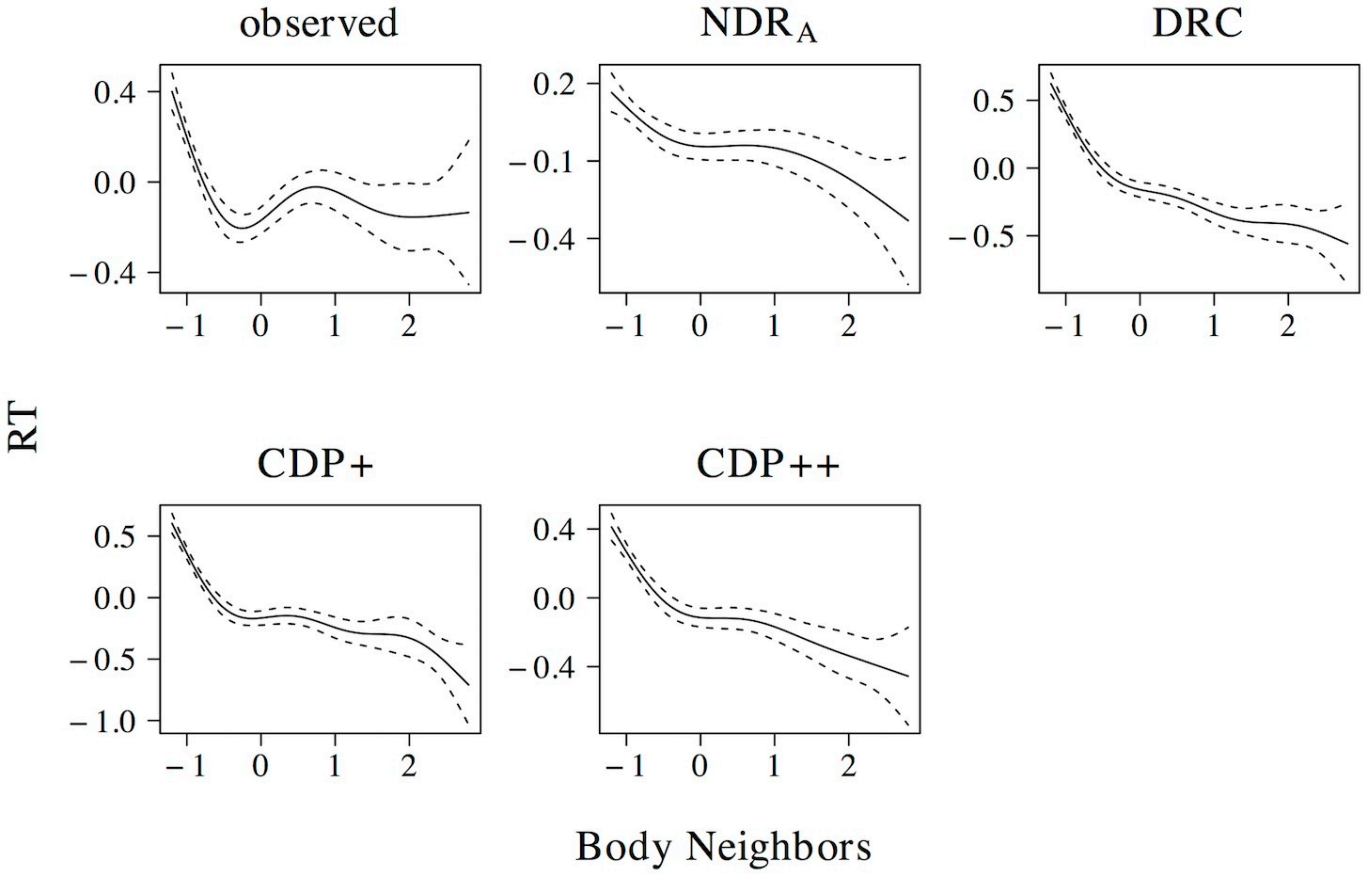
Body neighborhood density. The effect of body neighborhood density in word naming.

Fig 8 presents the effect of body neighborhood density. For the observed naming latencies, the effect of body neighborhood density is u-shaped in nature, with particular difficulties for words with few body neighbors. Although all models correctly predict that the effect should be most prominent for low predictor values, none of the models captures the non-linear effect of body neighborhood density to its full extent. The deviations of the simulated effect from the observed effect, however, are greater for the ndr_a_ than for the drc, the cdp+, and the cdp++.

In addition to effects in word naming, all models predict effects of both phonological (ndr_a_: *t* = −18.125, *β* = −0.395; drc: *t* = −17.159, *β* = −0.377; cdp+: *t* = −9.268, *β* = −0.214; cdp++: *t* = −7.523, *β* = −0.175) and body neighborhood density (ndr_a_: *t* = −9.021, *β* = −0.209; drc: *t* = −6.485, *β* = −0.152; cdp+: *t* = −9.057, *β* = −0.210; cdp++: *t* = −10.659, *β* = −0.245) in non-word naming. As was the case for the effect of orthographic neighborhood density, the non-linear estimates of these effects are qualitatively similar across models. All models predict a quadratic facilitatory effect that levels off for high predictor values for phonological neighborhood density and a more linear facilitatory effect of body neighborhood density.

#### The interplay of neighborhood density measures

As noted above, the ndr_a_ predicts that the effect of neighborhood density is primarily an orthographic neighborhood density effect, whereas several studies have argued that phonological or body neighborhood density characteristics may underlie the effect of orthographic neighborhood density. To investigate which neighborhood density measure drives the neighborhood effects, we entered all three predictors into a single linear regression model. Table 1 shows *t*-values and *β* coefficients for the neighborhood density measures in this model. When taking the effect of orthographic neighborhood density into account, phonological neighborhood density no longer has a significant effect on the observed naming latencies and body neighborhood density has a small inhibitory effect, which may be due to suppression [77].

**Table 1:** The linear interplay of orthographic, phonological and body neighborhood density. Listed are *t*-values and *β* coefficients for each of the predictors in an additive linear model.

	Orthographic N		Phonological N		Body N	
	*t*	*β*	*t*	*β*	*t*	*β*
observed	−13.733	−0.368	−0.929	−0.023	2.695	0.057
ndr_a_	−21.132	−0.524	−2.177	−0.049	8.146	0.159
drc	−1.728	−0.047	−0.387	−0.010	−13.476	−0.289
cdp+	−8.060	−0.213	−5.025	−0.121	−7.015	−0.146
cdp++	−4.412	−0.122	−3.723	−0.094	−6.072	−0.132

The ndr_a_ model captures the general pattern of results: orthographic neighborhood density remains highly significant, the effect of phonological neighborhood density has a small negative coefficient, and body neighborhood density becomes inhibitory. As in the individual models for the three predictors, however, the ndr_a_ somewhat underestimates the effect of body neighborhood density, which is reflected in an overly large *positive* *t*-value. The drc, cdp+, and cdp++ models have more significant problems with the interplay of the neighborhood density measures. All three models underestimate the contribution of orthographic neighborhood density and incorrectly predict strong inhibitory effects for body neighborhood density.

To further explore the interplay of the neighborhood density measures, we fitted two gams to assess the potential non-linear interplay of orthographic neighborhood density with phonological and body neighborhood density. The first gam includes a tensor product of orthographic neighborhood density and phonological neighborhood density, the second a tensor product of orthographic neighborhood density and body neighborhood density.

**Fig 9:**
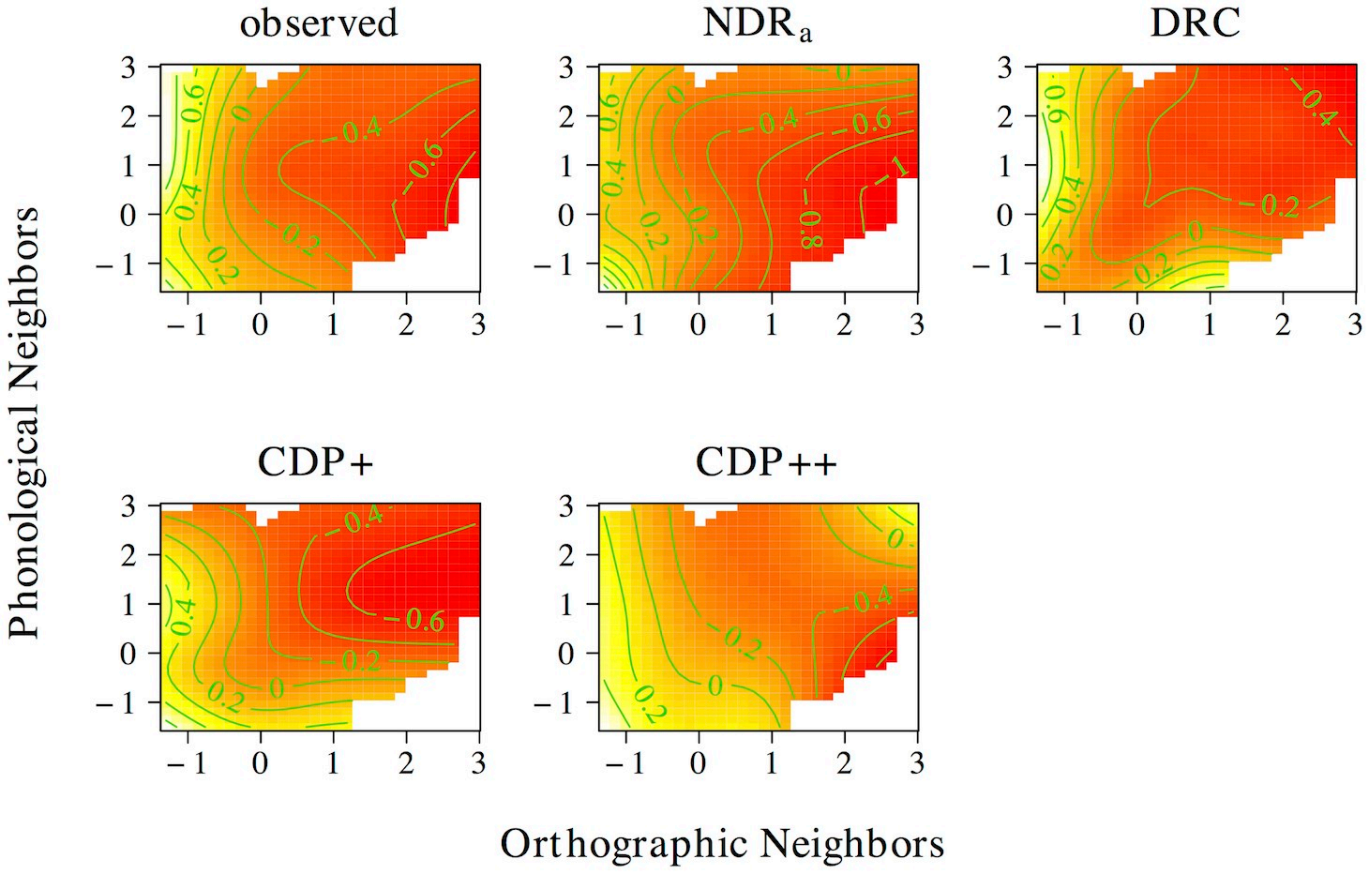
Interplay of orthographic and phonological neighborhood density. The non-linear interplay of orthographic neighborhood density and phonological neighborhood density in tensor product GAMs.

The results of the tensor product models for the interaction between orthographic neighborhood density and phonological neighborhood density are shown in Fig 9. Predictor values for orthographic neighborhood density are on the *x*-axis, whereas predictor values for phonological neighborhood density are on the *y*-axis. The *z*-axis visualizes adjustments to the average response time as a function of both predictors. Warmer colors (red) indicate shorter response times, whereas colder colors (yellow) indicate longer response times.

The pattern of results for the observed naming latencies is dominated by a strong facilitatory effect of orthographic neighborhood density. An effect of phonological neighborhood density is present for words with many orthographic neighbors only. For these words the effect of phonological neighborhood density is inhibitory. The ndr_a_ model captures the qualitative nature of the numerical interaction between orthographic neighborhood density and phonological neighborhood density and shows a pattern of results that is highly similar to that in the observed data. The cdp++ model captures the general nature of the interaction as well, albeit in a less accurate manner as compared to the ndr_a_. The drc and the cdp+ incorrectly predict a facilitatory effect of phonological neighborhood density for words with many orthographic neighbors.

**Fig 10:**
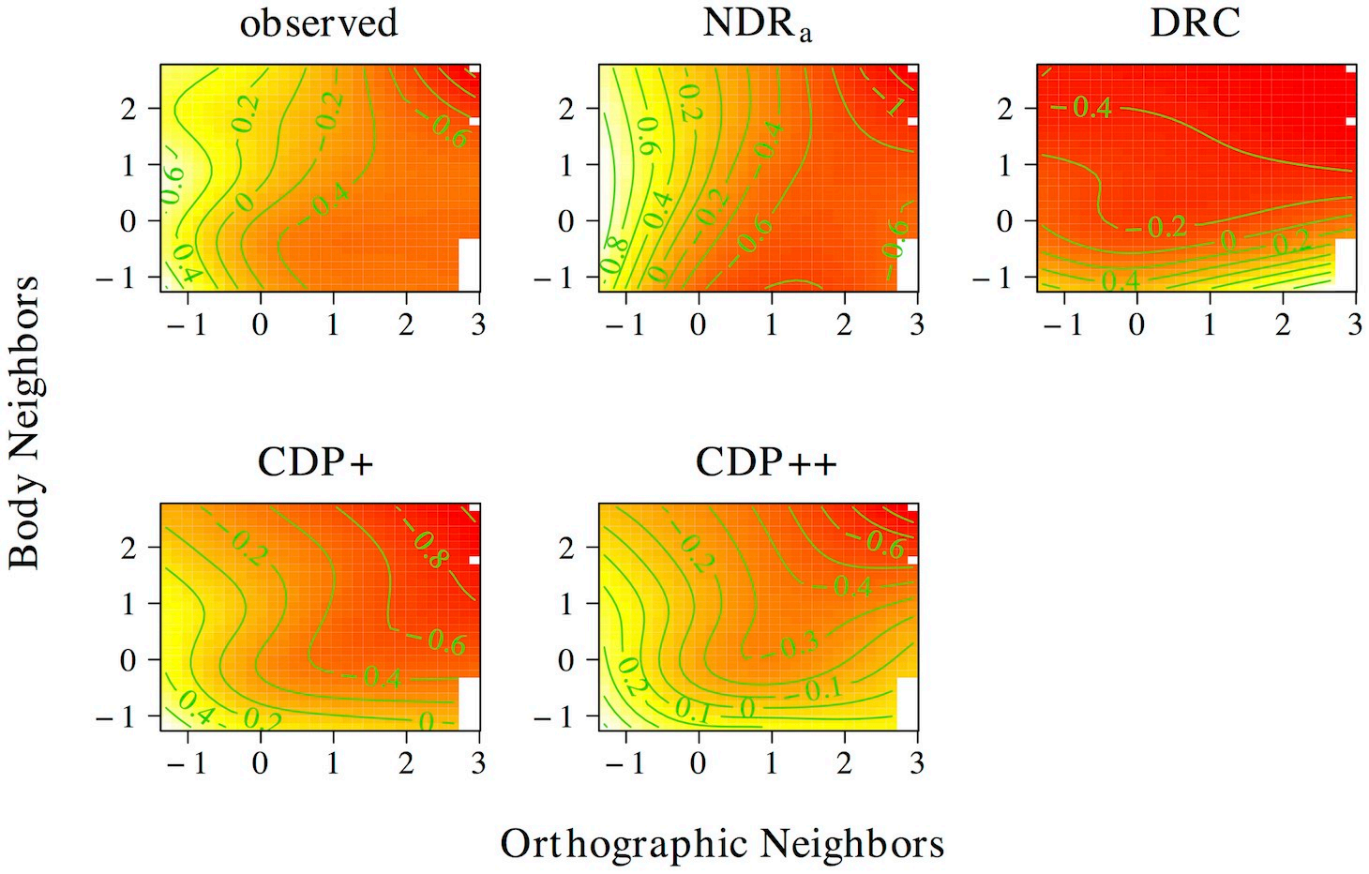
Interplay of orthographic and body neighborhood density. The non-linear interplay of orthographic neighborhood density and body neighborhood density in tensor product GAMs.

Fig 10 presents the non-linear interplay between orthographic neighborhood density and body neighborhood density in the observed data, as well as for each of the models under investigation. The observed naming latencies again show a strong facilitatory effect of orthographic neighborhood density. As was the case for phonological neighborhood density, an effect of body neighborhood density is present for words with many orthographic neighbors only. Unlike the effect of phonological neighborhood density, however, the effect of body neighborhood density is facilitatory in nature. The ndr_a_ simulates this pattern of results with remarkable accuracy. By contrast, the cdp+ and cdp++ underestimate the effect of orthographic neighborhood density for words with few phonological neighbors. The drc fails to capture the effect of orthographic neighborhood density and incorrectly predicts a main effect of body neighborhood density only.

Two conclusions can be drawn from the results of these simulations. First, the neighborhood density effect seems to primarily be an effect of *orthographic* neighborhood density. This argues against an interpretation of neighborhood effects as being driven by phonological or body neighborhood density. Second, the tensor product gams on the observed data indicate that the effect of orthographic neighborhood density is modulated by phonological and body neighborhood density for words with many orthographic neighbors. This effect is facilitatory for body neighborhood density and inhibitory for phonological neighborhood density. The correct characterization of this pattern by the ndr_a_ suggests that the model is sensitive to the influence of the neighborhood similarity structure that characterizes the lexical-distributional space in English on response patterns in the reading aloud task.

It is worth taking a moment to consider why the ndr_a_ model captures the complex interplay of the neighborhood density measures. Neighborhood effects in the ndr_a_ arise due to bottom-up co-activation of orthographic neighbors of the target word. When the orthographic word *bear* is presented, for instance, not only the corresponding lexeme *BEAR* is activated, but lexemes of orthographic neighbors such as *PEAR*, *WEAR*, *HEAR* and *YEAR* receive activation as well. The more lexemes are co-activated, the more activation spreads from these co-activated lexemes to the phonological level. The fact that the neighborhood density effects seem to primarily be driven by orthographic neighborhood density therefore follows straightforwardly from the architecture of the ndr_a_ model.

Body neighbors are words that share the orthographic rhyme with the target word. The more of the orthographic neighbors are body neighbors, the faster the second demi-syllable of the target word is activated, and the faster that target word is named. The effect of phonological neighborhood density is opposite to that of body neighborhood density. For words with many orthographic neighbors the observed naming latencies show an inhibitory effect of phonological neighborhood density: words with many phonological neighbors are named slower than words with few phonological neighbors. As counter-intuitive as this inhibitory effect of phonological neighborhood density might seem, it follows straightforwardly from the architecture of the ndr_a_ model.

In contrast to orthographic and body neighbors, the lexemes of phonological neighbors are not necessarily co-activated by the orthographic presentation of the target word. The orthographic presentation of the word *bear*, for instance, does not co-activate the lexical representations *HAIR* and *AIR*. *HAIR* and *AIR* therefore do not help activate the target word phonology, despite the fact that these lexemes share the second demi-syllable with *BEAR*. The model, however, has learned to associate *HAIR* and *AIR* with the word-final demi-syllable *8R*. The higher the number of lexemes that share a demi-syllable, the less well the association between each lexeme and that demi-syllable will be learned. The existence of the phonological neighbors *HAIR* and *AIR* therefore leads to a lower connection strength from the lexeme *BEAR* to the demi-syllable *8R*. This results in a longer naming latency for the word *bear* than would be the case if its phonological neighbors *hair* and *air* did not exist.

#### Pseudo-homophones

As noted by Coltheart et al. [1], the neighborhood density effects reported above are complemented by a pseudo-homophone effect in non-word naming [58, 78, 79]. Naming latencies for non-words that can be pronounced as real words (e.g., *bloo*) are shorter as compared to naming latencies for normal non-words. All models correctly predict a pseudo-homophone advantage. The effect size of this effect, however, is much larger in the drc (*t* = −18.241, *β* = −0.793) than in the other models (ndr_a_: *t* = −5.269, *β* = −0.248; cdp+: *t* = −2.686, *β* = −0.127; cdp++: *t* = −2.794, *β* = 0.132).

Additionally, there has been some debate as to whether or not there is a base word (e.g., *blue*) frequency effect for pseudo-homophones. In a review of the evidence, Reynolds and Besner [80] conclude that “the published data are most consistent with the conclusion that there is no base word frequency effect on reading aloud when pseudohomophones are randomly mixed with control nonwords”. In pure non-word blocks, however, an effect of base word frequency has been observed [81, 82]. In our non-word simulations, the naming latencies simulated by the drc model reveal a relatively strong base word frequency effect (*t* = −9.454, *β* = −0.280), whereas the ndr_a_ (*t* = −4.763, *β* = −0.153) and cdp+ (*t* = −2.668, *β* = −0.092) predict weaker base word frequency effects. A base word frequency effect is absent in the naming latencies simulated by the cdp++ model (*t* = 0.050, *β* = 0.002). The results of the current simulations suggest that further experimental work on base word frequency effects for pseudo-homophones could help inform the architecture of reading aloud models.

#### Orthographic neighborhood size by frequency

**Fig 11:**
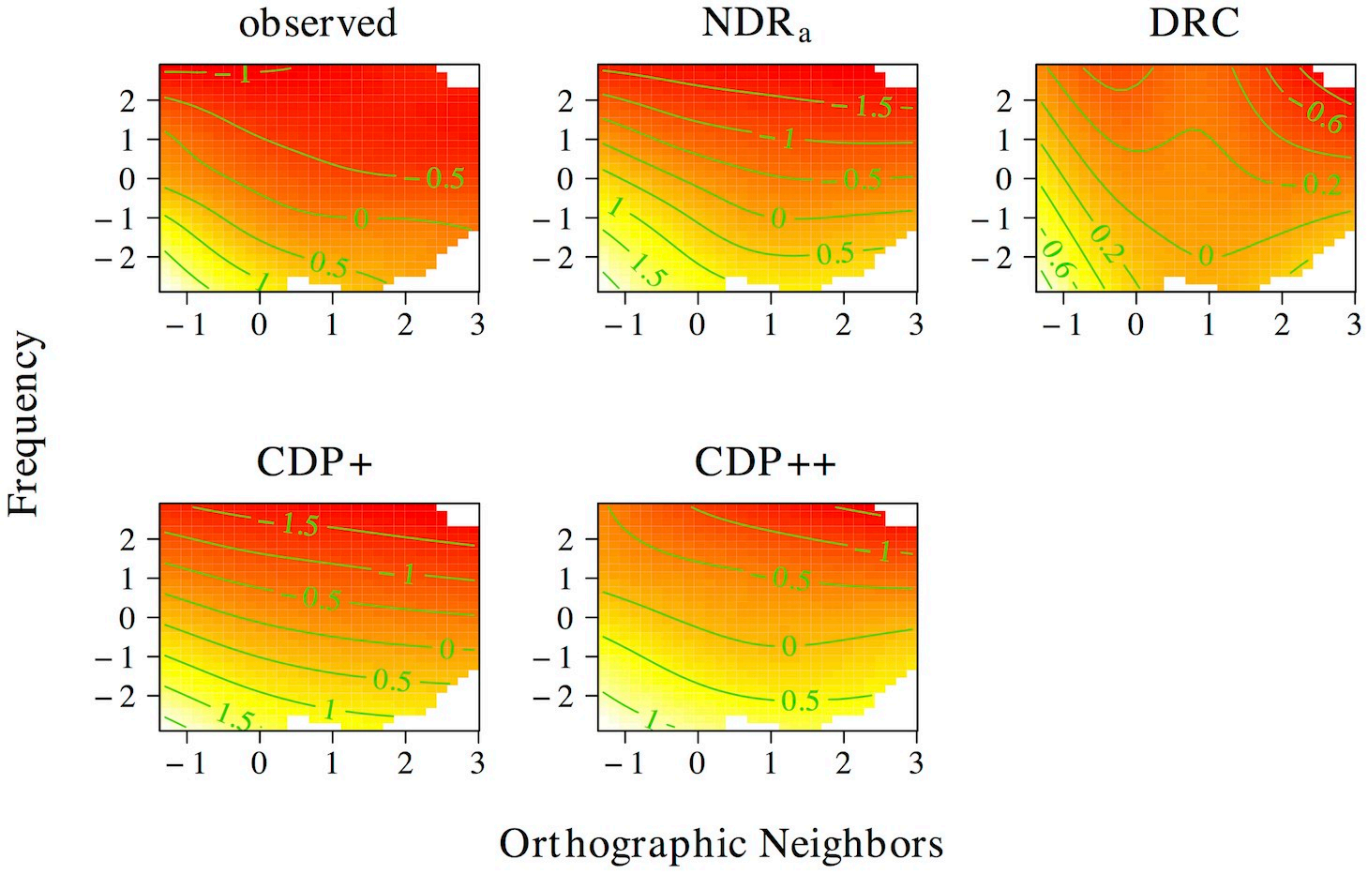
Frequency by neighborhood density interaction. The interaction of frequency with orthographic neighborhood density and phonological neighborhood density in tensor product GAMs.

A further important neighborhood density effect concerns the interaction of orthographic neighborhood density with frequency. Several studies found that low frequency, but not high frequency words are read faster when they have many neighbors [69, 70, 83]. We fitted tensor product gams to look at the interaction of frequency and neighborhood density in the observed and simulated naming latencies. The results of this model are shown in Fig 11. The observed data show the expected pattern of results: a facilitatory effect of neighborhood density that is most prominent for low frequency words. All models capture the general nature of the frequency by orthographic neighborhood density interaction and show the longest latencies for low frequency words with few orthographic neighbors. The simulated effects in the ndr_a_, the cdp+, and the cdp++, however, resemble the orthographic neighborhood density by frequency interaction in the observed naming latencies more closely than the simulated effect in the drc.

### Consistency/Regularity effects

#### Regularity

The relation between the orthography and phonology of a word has been a hotly debated topic in the reading aloud literature. Coltheart et al. [1] focused on the concept of regularity and defined a word as regular “if its pronunciation is correctly generated by a set of grapheme to phoneme conversion rules” [1] (p. 231). The drc model predicted that regular words should be pronounced faster than irregular words. This was confirmed by a number of experimental findings [84–87]. We therefore consider the effect of regularity a good starting point for the investigation of the relation between orthography and phonology.

In our simulations we defined regularity as a two-level factor, based on the regularity of a word given the grapheme to phoneme (henceforth gpc) rules underlying the sub-lexical route of the drc model. A linear model on the elp naming latencies shows the predicted facilitation for regular words (*t* = −9.121, *β* = −0.403). This effect is somewhat underestimated by the ndr_a_ (*t* = −6.637, *β* = −0.295) and somewhat overestimated by the cdp+ (*t* = −14.807, *β* = −0.637) and the cdp++ (*t* = −12.015, *β* = −0.524). The drc model dramatically overestimates the effect of regularity (*t* = −41.810, *β* = −1.440).

#### Position of irregularity

The size of the regularity effect depends on the position at which the irregularity occurs. A number of studies found larger irregularity effects for words with early-position irregularities as compared to words with late-position irregularities [18, 88, 89]. A similar effect of position of irregularity is present in the elp naming latencies (*t* = −4.934, *β* = −0.732) and the naming latencies simulated by the drc (*t* = −6.058, *β* = −1.099), the cdp+ (*t* = −2.954, *β* = −0.533), and the cdp++ (*t* = −3.755, *β* = −0.736). The ndr_a_ model, however, fails to capture this effect (*t* = 1.424, *β* = 0.200). The inability of the ndr_a_ to model the position of irregularity effect is not surprising given the fact that the model is insensitive to the sequential nature of the orthographic input and the phonological output. We return to this issue in the discussion section.

#### Consistency

A number of studies have investigated non-binary measures of the relationship between the orthography and the phonology of a word. Following Glushko [90], these studies adopted measures of consistency, rather than regularity. Originally, Glushko [90] defined consistency as a two-level factor, for which words were defined as inconsistent if their orthographic body mapped onto more than one phonemic sequence. For instance, while the pronunciation of the word *wave* is correctly predicted by the gpc rules of the drc model it is inconsistent, because its word body is pronounced differently in the word *have*.

**Fig 12:**
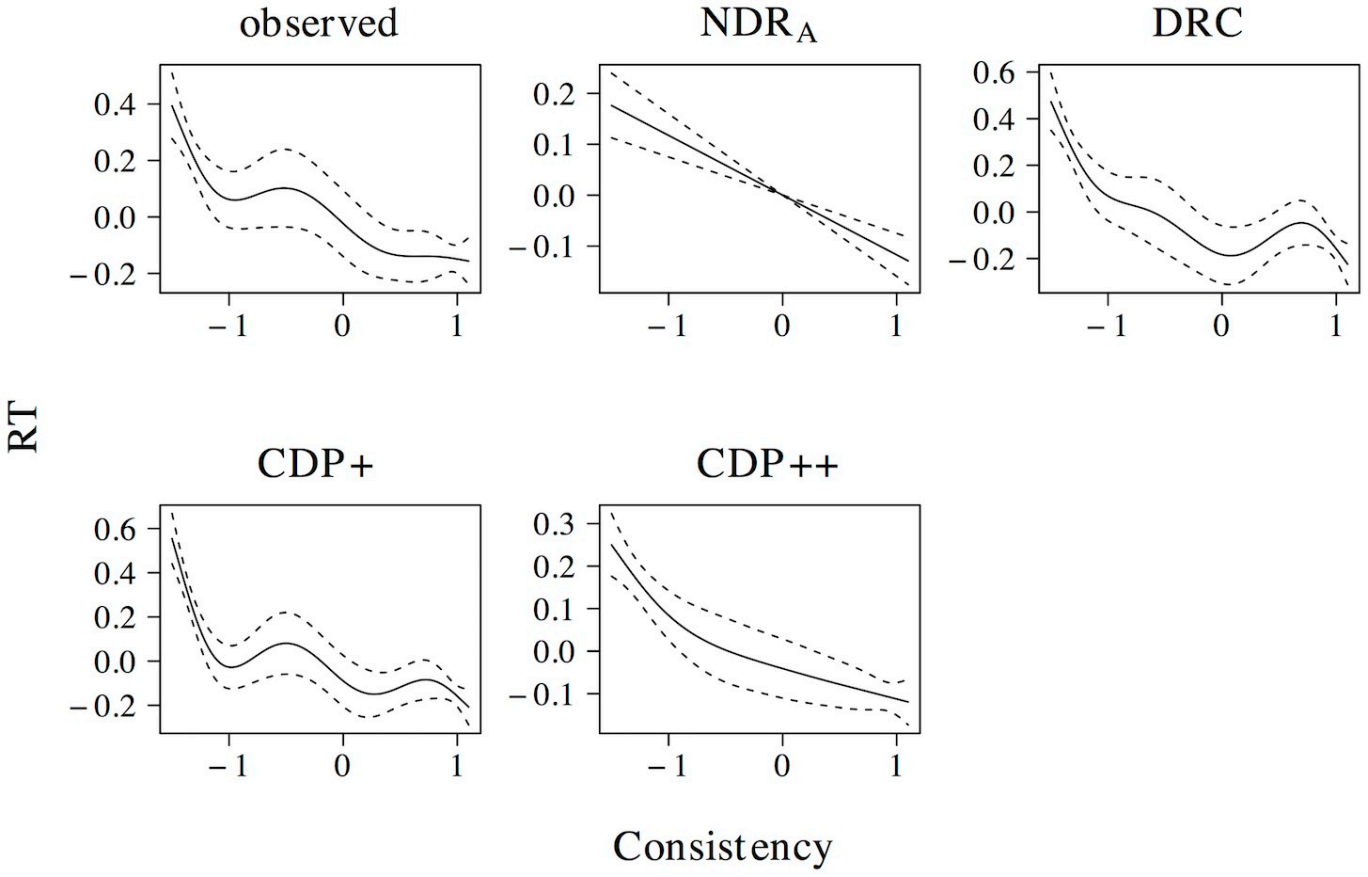
Consistency. The effect of consistency of the orthography to phonology mapping in word naming.

Further research indicated that consistency is better conceptualized as a continuous variable [8, 18, 76, 91]. We tested a number of consistency measures and found the proportion of consistent word tokens to explain most variance in the elp naming latencies (*t* = −8.281, *β* = −0.171). This linear effect of consistency was captured by all models (ndr_a_: *t* = −5.553, *β* = −0.117; drc: *t* = −8.850, *β* = −0.190; cdp+: *t* = −9.671, *β* = −0.188; cdp++: *t* = −7.809, *β* = −0.127). Fig 12 shows the non-linear effect of consistency. The consistency effect is more prominent for low predictor values in the observed naming latencies. The drc, cdp+, and cdp++ capture the general nature of this non-linearity, although the cdp+ overestimates its strength and the cdp++ underestimates its strength. The ndr_a_ fails to capture the non-linear effect of consistency effect and, instead, predicts a linear effect. Given the width of the confidence intervals for the observed effect of consistency, however, it is unclear how pronounced the non-linearity of the consistency effect in the observed data is.

In addition to the effect of consistency in word naming, a consistency effect has also been observed in non-word naming [90, 92]. For our set of non-words, all models predict a facilitatory effect of consistency (ndr_a_: *t* = −9.276, *β* = −0.215; drc: *t* = −7.074, *β* = −0.166; cdp+: *t* = −12.941, *β* = −0.294; cdp++: *t* = −10.309, *β* = −0.238). Non-linear models revealed that the ndr_a_ predicts a non-linear effect of consistency for nonwords that is similar in nature to the effect of consistency for words in the observed naming latencies, as does the drc. By contrast, the cdp+ and cdp++ predict that the effect of consistency for nonwords is more prominent for high rather than low predictor values. As was the case for words, however, the confidence intervals of the predicted effects were relatively large.

The ndr_a_ ($\frac{\beta_{nw}}{\beta_{w}}=1.834$), the cdp+ (($\frac{\beta_{nw}}{\beta_{w}}=1.560$), and the cdp++ ($\frac{\beta_{nw}}{\beta_{w}}=1.875$) all predict a larger magnitude of the consistency effect in non-word naming than in word naming. By contrast, the drc model predicts similar effect sizes in word and non-word naming ($\frac{\beta_{nw}}{\beta_{w}}=0.871$). The prediction of the drc model fits well with the findings of Glushko [90], who found a 29 ms facilitatory effect of consistency in both word and non-word naming. In the absence of a large-scale database of non-word naming latencies or further experimental findings, however, any conclusions regarding the simulation of the relative effect sizes of the effects of consistency in word and non-word naming in the ndr_a_ and cdp+ models are tentative.

#### Consistency by regularity

Now that we established the presence of both a consistency and regularity effect in the observed naming latencies, we return to the question of which measure best characterizes the effect of the orthography to phonology mapping on naming latencies. It is problematic for the drc model if an independent graded consistency effect is present on top of the regularity effect, because its sub-lexical route is based on hard-coded rules that operate in an all-or-none fashion [92, 93]. In contrast, the cdp+ and cdp++ models are sensitive to the probabilistic characteristics of orthography to phonology mappings [9, 94]. These models therefore allow for the possibility of a graded consistency effect over and above the effect of regularity.

In the ndr_a_ model, regularity and consistency effects originate from the co-activation of lexical items with similar orthographies. The word *band* co-activates the lexical representations of phonologically consistent words like *hand*, *sand* and *land*. These words provide additional support for the target demi-syllable {*nd* and hence speed up naming latencies. In contrast, *bough* co-activates the lexemes of phonologically inconsistent neighbors, such as *tough*, *rough* and *cough*. The lexemes corresponding to these inconsistent neighbors activate the non-target demi-syllable *Vf* and therefore do not facilitate the pronunciation of the target word *bough*. The amount of support for the target demi-syllables directly depends on the number of co-activated lexemes of orthographically consistent and inconsistent words. The ndr_a_ therefore predicts that graded consistency should be a better measure of orthography to phonology mapping effects than regularity.

An inspection of the naming latencies in the elp revealed not only an independent contribution of both regularity and consistency, but also a significant interaction between both measures. Table 2 shows the results of a linear model that includes regularity, consistency and a regularity by consistency interaction term. For the observed naming latencies, the strongest effect is that of consistency. The effect of regularity becomes weaker in a model that includes consistency, but remains significant. Furthermore, there is a significant interaction of regularity with consistency. The ndr_a_ captures the general pattern of results, with a stronger main effect of consistency, a weaker main effect of regularity, and a positive estimate for the interaction between consistency and regularity. The interaction between consistency and regularity, however, fails to reach significance (*t* = 1.574, *p* = 0.116).

**Table 2:** The interplay of regularity and consistency. Listed are *t*-values and *β* coefficients for each of the predictors in an additive linear model.

	Consistency		Regularity		Interaction	
	*t*	*β*	*t*	*β*	*t*	*β*
observed	−6.182	−0.255	−3.226	−0.167	3.040	0.146
ndr_a_	−3.727	−0.158	−2.350	−0.125	1.574	0.079
drc	−0.038	−0.001	−36.731	−1.521	−0.797	−0.031
cdp+	−5.747	−0.221	−7.371	−0.357	2.292	0.103
cdp++	−2.232	−0.072	−8.493	−0.343	−0.661	0.025

The drc dramatically overestimates the effect of regularity and fails to predict the main effect of consistency. The grapheme to phoneme conversion rules in the drc model thus fail to capture the independent contribution of graded orthography-to-phonology consistency. The cdp+ and cdp++ models capture the main effects of both consistency and regularity. Both models, however, incorrectly predict a larger effect size of regularity as compared to consistency. The cdp+ model captures the interaction between consistency and regularity, whereas the cdp++ model does not.

**Fig 13:**
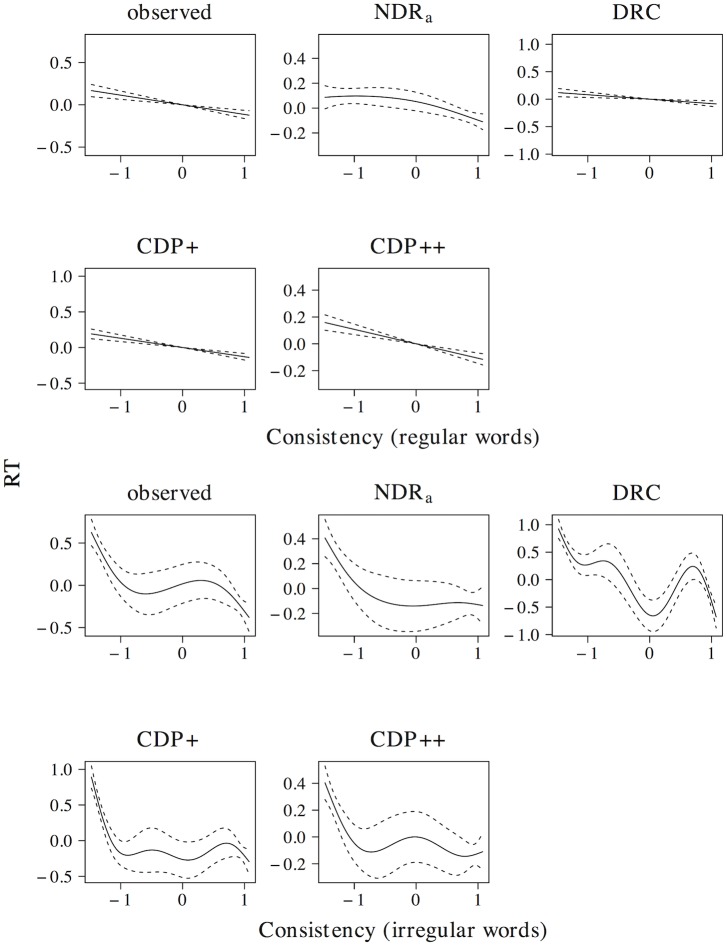
Interplay of consistency and regularity. The interplay of consistency and regularity in word naming. Top two rows shows results for regular words, bottom two rows for irregular words.

Fig 13 shows the non-linear interaction between consistency with regularity, which sheds further light on the issue. Regular words (top two rows) show a subtle linear effect in the observed data, as well as in the simulations of the all models. For irregular words, however, a non-linear curve characterizes the elp naming latencies, with particularly long reaction times for inconsistent irregulars. The general shape of this curve is captured by all models, although the drc yields a more complex non-linear effect that is not present in the observed data.

#### Consistency by friends-enemies

Consistency has also been shown to interact with another measure of the consistency of the orthography-to-phonology mapping: the number of friends (words with the same body and rime pronunciation) and enemies (words with a different body and rime pronunciation) a word has. Jared [91, 95], for instance, found an effect of consistency that was limited to words with more enemies than friends. Different friend-enemy measures have been proposed. Here, we use the measure that explained most of the variance in the elp naming latencies, which is the number of friends minus the number of enemies (*t* = −6.357, *β* = −0.132). All models revealed a significant main effect of this friend-enemy measure on the simulated naming latencies (ndr_a_: *t* = −2.754, *β* = −0.059; drc: *t* = −16.222, *β* = 0.335; cdp+: *t* = −8.900, *β* = −0.174; cdp++: *t* = −5.393, *β* = −0.088). The ndr_a_ somewhat underestimates its effect size, whereas the drc overestimates its effect size.

**Fig 14:**
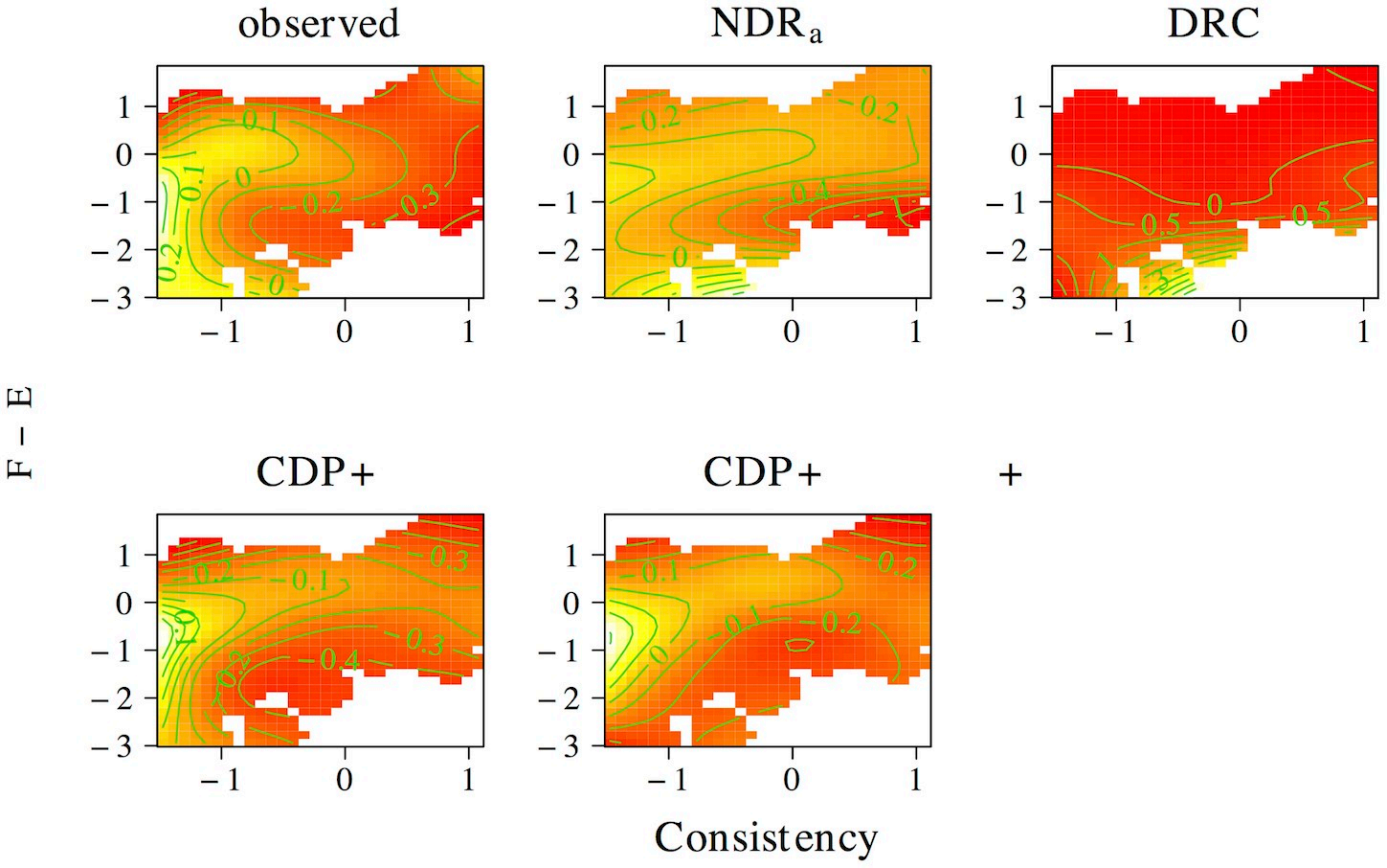
Interplay of consistency and friends minus enemies. The interaction of consistency with friends minus enemies in tensor product GAMs.

More interestingly, the observed data support a tensor product gam with an interaction between consistency and our friend-enemy measure. This interaction is displayed in Fig 14. The non-linear interaction between consistency and the friend-enemy measures is highly complex in nature, with the consistency effect being most prominent for medium values of the friend-enemy measure. The ndr_a_, cdp+, and cdp++ succeed in simulating the general nature of this interaction. By contrast, the drc incorrectly predicts a null effect of consistency for most values of the friend-enemy measure.

#### Consistency by frequency

A final effect of consistency/regularity that warrants some discussion is the interaction of frequency with these measures. Jared [91, 95] did not find evidence for an interaction of either regularity or consistency with frequency. As noted by Perry et al. [2], these null results stand in contrast to previous studies [84–87] that reported longer naming latencies for irregular or inconsistent low-frequency words, but not for high-frequency words. The elp naming latencies revealed similar aic scores for a model with a tensor product interaction of consistency and frequency (aic: 4989.23) and a model with separate smooths for consistency and frequency (aic: 4988.50). The evidence for a consistency by frequency interaction in the elp naming latencies, therefore, is subtle at best.

**Fig 15:**
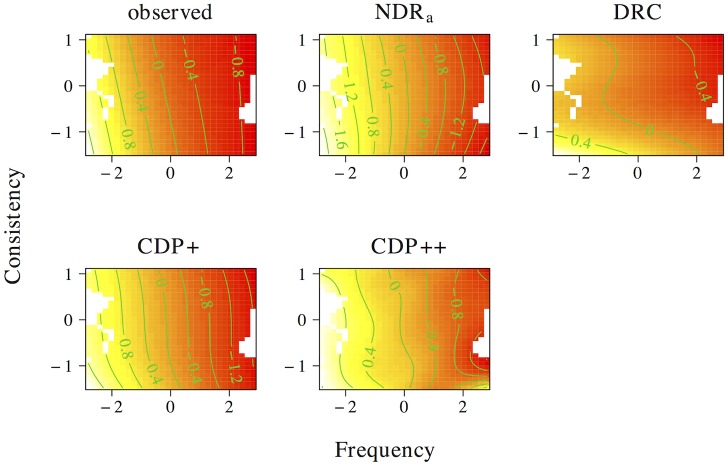
Interplay of frequency and consistency. The interaction of frequency with consistency in tensor product GAMs.

For completeness, we nonetheless show the results of the tensor product gam in Fig 15. The panel for the observed data shows a subtle interaction in the expected direction, with a consistency effect that is more prominent for low frequency than for high frequency words. The ndr_a_, the cdp+, and the cdp++ simulations predict a qualitatively similar subtle interaction. The current simulations therefore suggest that these models are capable of explaining the subtle interplay between consistency and frequency. The drc, by contrast, predicts a somewhat different pattern of results, with similar effect sizes of orthographic neighborhood density across the word frequency range.

### Frequency effects

#### Frequency

**Fig 16:**
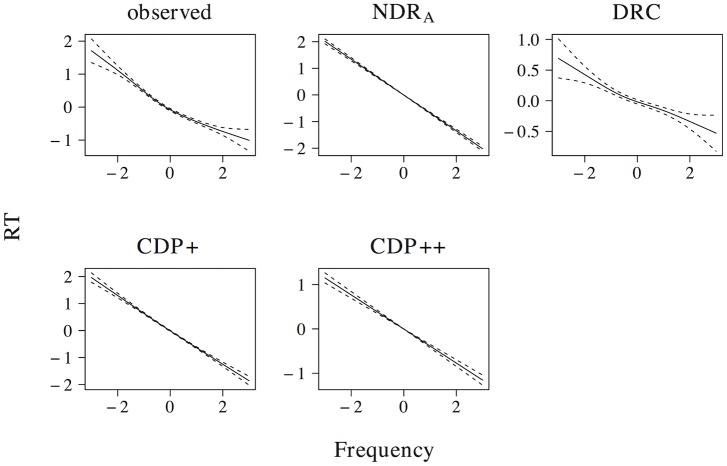
Frequency. The effect of frequency in word naming.

The lexical predictor with the highest correlations with observed naming latencies is word frequency. The effect of frequency is well-established (see e.g. [59, 65, 95, 96]) and is highly significant in the observed naming latencies (*t* = −25.333, *β* = −0.454). As expected, all models capture the frequency effect (ndr_a_: *t* = −44.748, *β* = −0.670; drc: *t* = −8.790, *β* = −0.174; cdp+: *t* = −40.441, *β* = −0.631; cdp++: *t* = −20.654, *β* = −0.383). As can be seen in Fig 16 the effect is linear or near-linear in the observed data, as well as in all model simulations.

#### Familiarity

**Fig 17:**
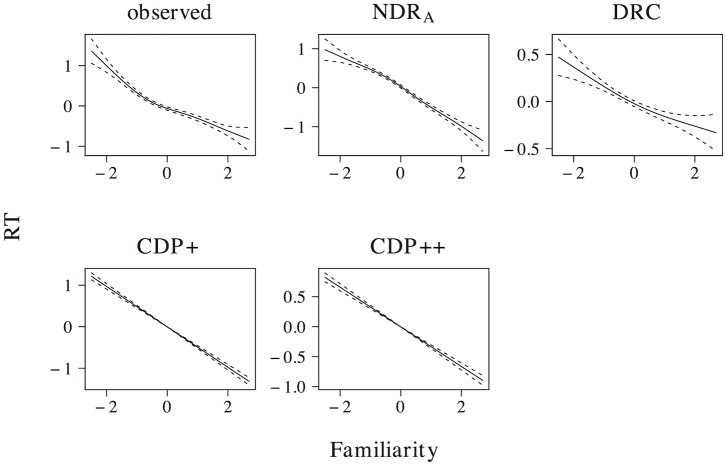
Familiarity. The effect of familiarity in word naming.

In addition to the frequency effect, we also investigated the effect of familiarity on the elp naming latencies. As can be seen in Fig 17, the effect of familiarity in the observed data is linear and highly similar to that of frequency (*t* = −17.760, *β* = −0.348). This is unsurprising, given the high correlation between both measures (*r* = 0.770). For completeness, we nonetheless include a description of the effect of familiarity here. All models capture the general linear trend of this effect (ndr_a_: *t* = −25.464, *β* = −0.474; drc: *t* = −7.063, *β* = −0.154; cdp+: *t* = −28.939, *β* = −0.487; cdp++: *t* = −22.267, *β* = −0.330). While the observed data and the naming latencies simulated by the drc model reveal an effect that slightly levels off for high predictor values, however, the naming latencies simulated by the ndr_a_, cdp+, and cdp++ models are characterized by a linear effect of familiarity.

#### Bigram frequency

All models accurately capture the frequency effect at the word level. Frequency effects, however, also exists at a finer grain size. Baayen et al. [68], for instance, found an effect of bigram frequency on word naming latencies. In the ndr_a_, bigrams have explicit representations at the orthographic level, and many of the demi-syllable representations at the phonological level are diphones. In the cdp+ and cdp++ models, no explicit bigram representations exist. We therefore hypothesized that there might be an advantage for the ndr_a_ over these models with respect to bigram frequency effects.

Here, we explore the effect of two measures of orthographic bigram frequency: summed bigram frequency and mean bigram frequency. Both of these measures were predictive for the elp naming latencies (summed bigram frequency: *t* = 7.517, *β* = 0.148; mean bigram frequency: *t* = 11.392, *β* = 0.231). The ndr_a_ simulates the linear effect of both summed (*t* = 17.823, *β* = 0.335) and mean bigram frequency (*t* = 28.996, *β* = 0.517). Consistent with the effect of word frequency, the effect sizes in the ndr_a_ are larger than those in the observed data. As we clarify in the section on the overall fit of the model below, however, the effects of the bigram frequency measures in the ndr_a_ have the correct relative magnitude as compared to the effects of other lexical predictors.

The cdp+ and cdp++ also capture the effects of summed bigram frequency (cdp+: *t* = 3.091, *β* = 0.062; cdp++: *t* = 3.018, *β* = 0.060) and mean bigram frequency (cdp+: *t* = 9.777, *β* = 0.190; cdp++: *t* = 10.361, *β* = 0.166), although both models underestimate the effect size of the summed bigram frequency effect. The drc captures the effect of mean bigram frequency (*t* = 5.998, *β* = 0.130), but does not predict a significant effect of summed bigram frequency (*t* = 1.198, *β* = 0.024, *p* = 0.231).

**Fig 18:**
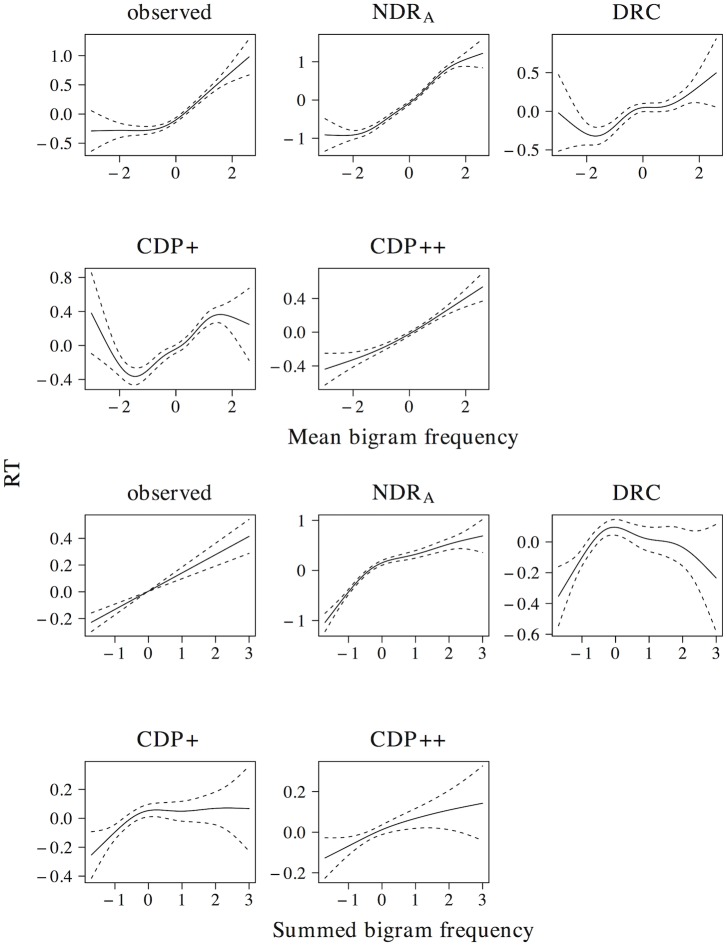
Mean bigram frequency and summed bigram frequency. The effects of mean bigram frequency (top two rows) and summed bigram frequency (bottom two rows) in word naming.

Fig 18 shows the results of a non-linear model for mean (top two rows) and summed (bottom two rows) bigram frequency. In the observed naming latencies, there is a facilitatory effect of mean bigram frequency that increases in size for larger values of bigram frequency. The ndr_a_ captures this pattern of results, as does the drc. The cdp++ model fails to capture the non-linear effect of mean bigram frequency, and instead predicts a linear effect. The cdp+, by contrast, overestimates the quadratic component of the effect for low predictor values, although the confidence intervals for this part of the predictor range are wide. The effect of summed bigram frequency is linear in the observed naming latencies. All models correctly predict facilitation for the lowest values of summed bigam frequency. The models, in particular the drc and the cdp+, however, incorrectly predict that the effect would level off for higher summed bigram frequencies.

**Fig 19:**
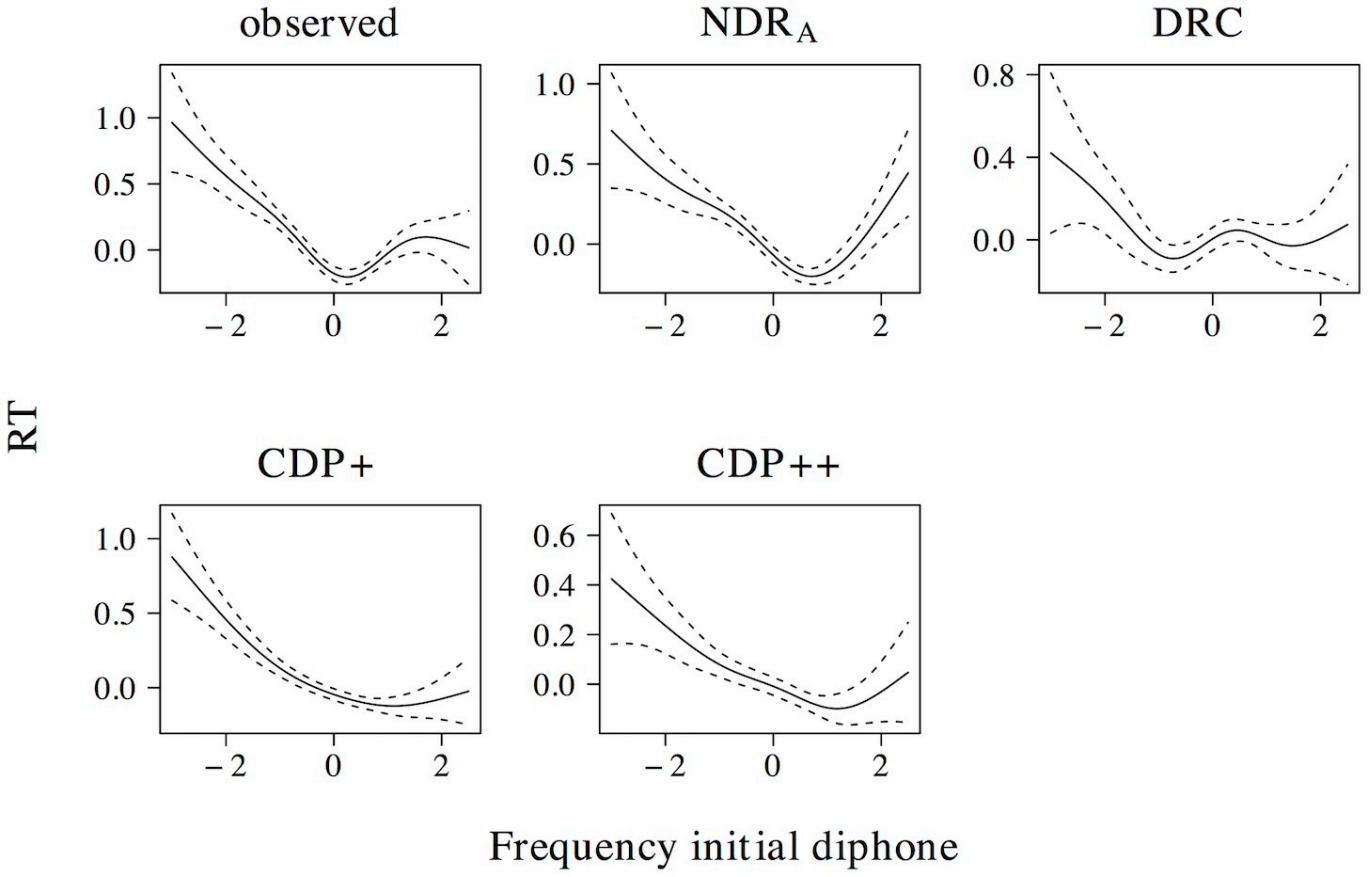
Initial diphone frequency. The effect of the frequency of the initial diphone in word naming.

In addition to the effect of orthographic bigram frequency, we also investigated the effect of phonological bigram frequency. The observed naming latencies showed a facilitatory linear effect of the frequency of the initial diphone (*t* = −6.700, *β* = −0.139). The ndr_a_ (*t* = −5.382, *β* = −0.114) captures this linear effect, as do the cdp+ (*t* = −6.670, *β* = −0.131) and the cdp++ (*t* = −4.558, *β* = −0.075). The drc, however, does not (*t* = −0.975, *β* = −0.019). As can be seen in Fig 19, the non-linear effect is u-shaped in nature, with greater naming latencies for words with low-frequency initial diphones and—to a lesser extent—for words with high frequency initial diphones. All models successfully capture the nature of this non-linear effect. The ndr_a_, however, overestimates the difficulty for words with high frequency initial diphones. Given the sparsity of data points at the high end of the predictor range and the resulting increased width of the confidence intervals, however, strong conclusions about the performance of the models for words with high frequency initial diphones would be premature.

### Semantic predictors

A final class of effects we investigated are the effects of semantic predictors. In particular, we report the simulation results for two types of semantic measures: synonym sets and morphological family size.

#### Synonym sets

We investigated the effects of two predictors that are based on the number of synonym sets that a word appears in (as listed in WordNet [97]). The more different meanings a word has, the more synsets it appears in and the faster it is named [68]. Following Baayen et al. [68], we consider two related measures: the number of simplex synsets and the number of complex synsets. The number of simple synsets simply refers to the number of synsets a word occurs in. The number of complex synsets is defined as the number of synsets in which a word is part of a compound or phrasal unit.

Both measures have an inhibitory effect on the observed naming latencies, which is slightly larger for the number of complex synsets (*t* = −13.272, *β* = −0.267) than for the number of simplex synsets (*t* = −11.118, *β* = −0.228). The ndr_a_ correctly simulates this pattern of results, although it overestimates the difference between the effect sizes for both predictors (number of simplex synsets: *t* = −13.962, *β* = −0.286; number of complex synsets: *t* = −22.475, *β* = −0.428). By contrast, the drc predicts a larger effect of the number of simplex synsets (*t* = −5.797, *β* = −0.127) than for the number of complex synsets (*t* = −4.797, *β* = −0.105). Consistent with the observed naming latencies, the naming latencies simulated by the cdp+ and cdp++ reveal somewhat larger effect sizes for the number of complex synsets (cdp+: *t* = −20.583, *β* = −0.373; cdp++: *t* = −15.886, *β* = −0.247) than for the number of simplex synsets (cdp+: *t* = −20.227, *β* = −0.371; cdp++: *t* = −14.410, *β* = −0.228). The difference between the effect sizes for the effects for both predictors in the cdp+, however, is marginal.

**Fig 20:**
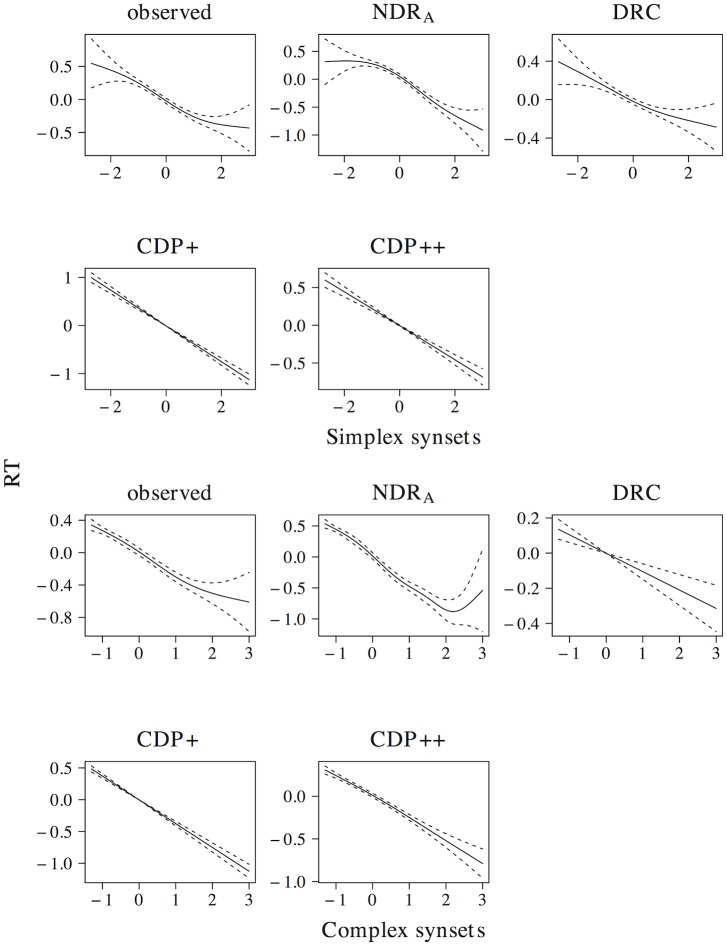
Number of synsets. The effects of the number of simplex (top two rows) and complex (bottom two rows) synsets in word naming.

Fig 20 presents the effect of both predictors, which are linear or near-linear in the elp naming latencies, as well as in the simulated naming latencies in all models. The effects of both predictors in the observed naming latencies as well as the naming latencies simulated by the ndr_a_ and drc models show some non-linearity for high predictor values. Again, however, given the sparsity of data points at both ends of the predictor range and the resulting wide confidence intervals, the statistical robustness of this non-linearity is questionable.

#### Morphological family size

**Fig 21:**
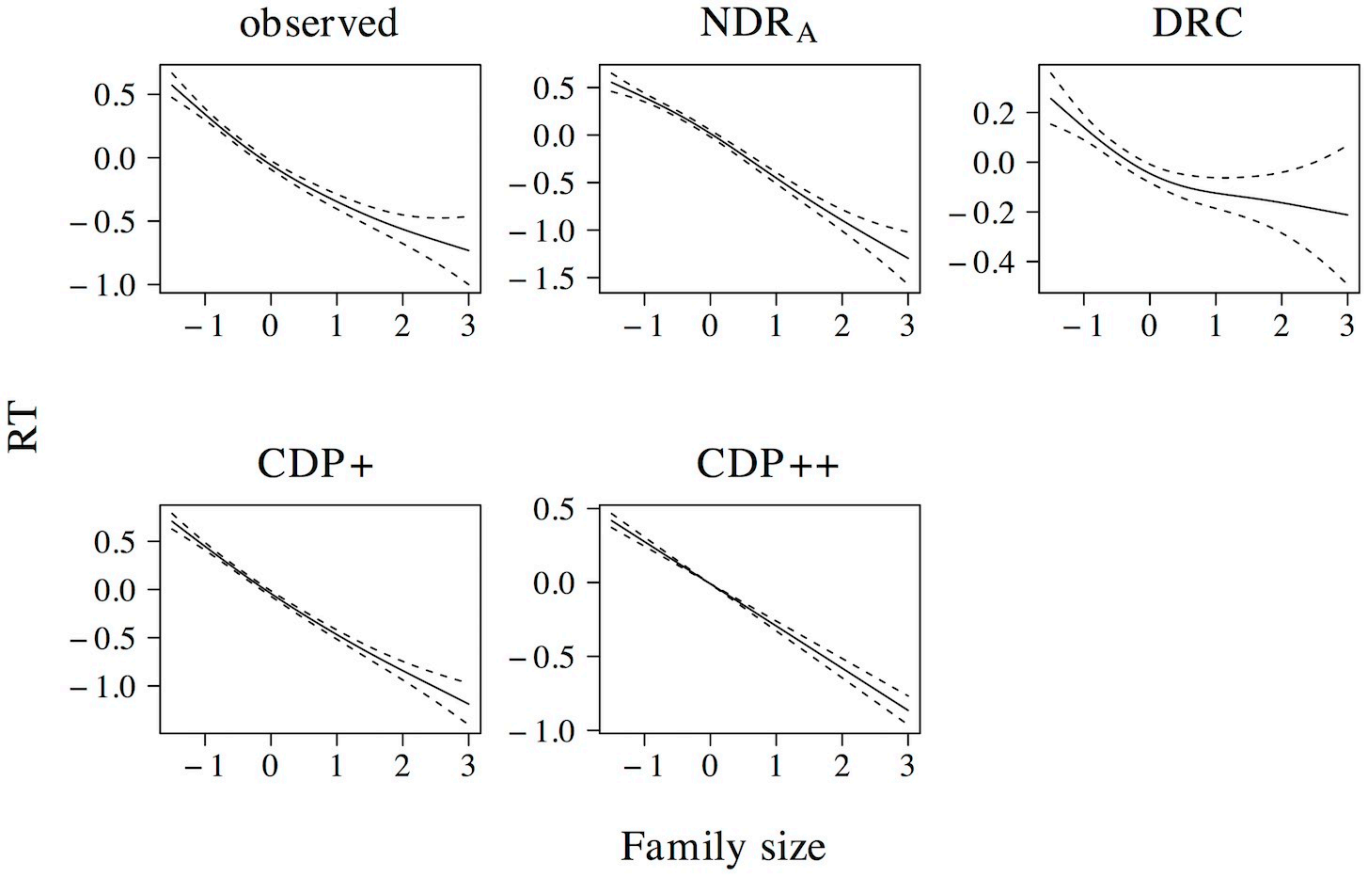
Family size. The effect of family size in word naming.

A second type of semantic predictor we looked at is morphological family size. Morphological family size is defined as the number of morphologically complex words in which a word occurs as a constituent (see, e.g. [98]). Words that occur in many complex words (such as *work*) are named faster than words that occur in fewer complex words [68]. This facilitatory effect of family size was confirmed in the elp naming latencies (*t* = 15.381, *β* = −0.305). All models correctly simulate this effect of family size (ndr_a_: *t* = −21.503, *β* = −0.413; drc: *t* = −5.209, *β* = −0.114; cdp+: *t* = −24.365, *β* = 0.427; cdp++: *t* = −18.371, *β* = −0.280). The drc model, however, substantially underestimates the magnitude of the effect. As can be seen in Fig 21, the effect of family size in a non-linear gam is similar in the observed naming latencies and in the naming latencies simulated by the models.

**Fig 22:**
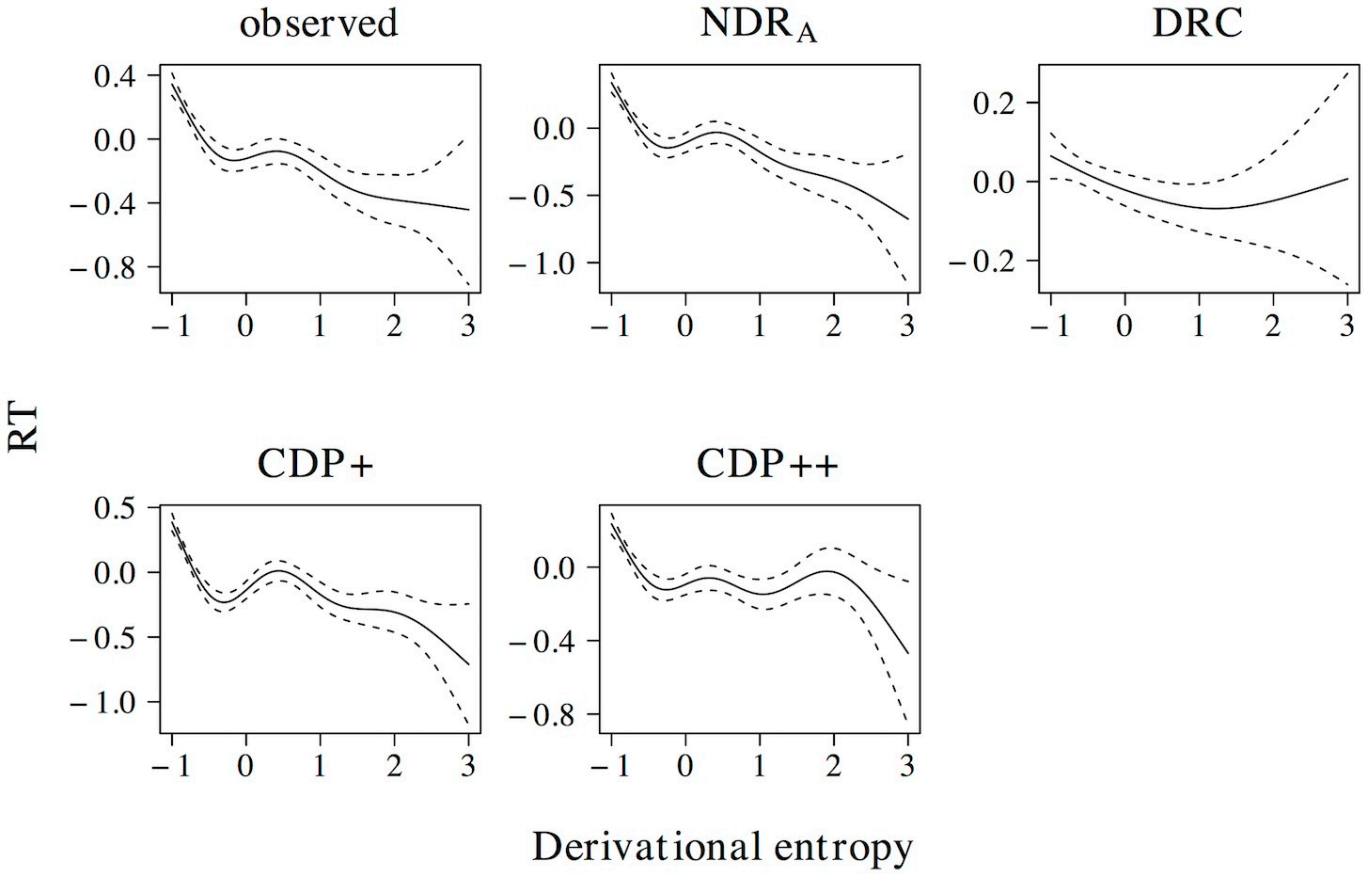
Derivational entropy. The effect of derivational entropy in word naming.

A related measure is derivational entropy [99]. Derivational entropy is the entropy [36] over the probabilities of a word’s morphological family members. As such, it provides an alternative to the family size measure, with family members weighted for their token frequency. Similar to the effect of family size, derivational entropy showed a facilitatory effect in the observed naming latencies (*t* = 8.984, *β* = −0.185) that was correctly simulated by all models (ndr_a_: *t* = −10.180, *β* = −0.211; drc: *t* = −2.201, *β* = 0.048; cdp+: *t* = −9.552, *β* = −0.186; cdp++: *t* = −6.743, *β* = −0.110). As was the case for the effect of family size, however, the drc underestimates the effect size of the effect of derivational entropy. A non-linear model of derivational entropy on the observed naming latencies revealed a fairly complex non-linear pattern of results. As can be seen in Fig 22, the ndr_a_, cdp+, and—to a lesser extent—the cdp++ models capture this non-linear pattern with remarkable accuracy. The drc replicates the facilitatory nature of the effect, but fails to capture its non-linear subtleties.

### Predictor effect sizes

**Fig 23:**
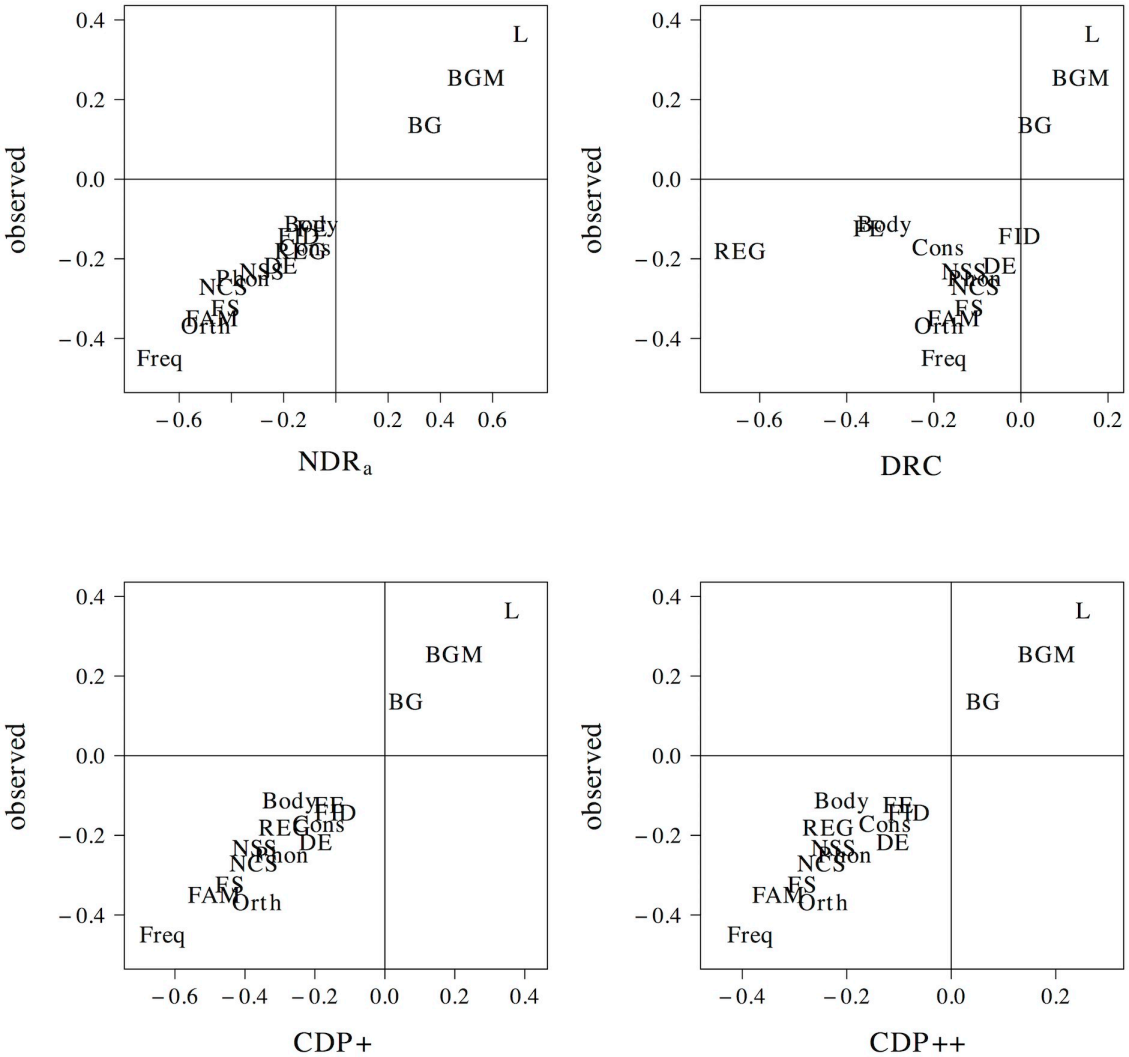
Predictor effect sizes. Comparison of predictor coefficients for the observed data and the simulations of the ndr_a_ (top left panel), drc (top right panel), CDP+ (bottom left panel), and cdp++ (bottom right panel) models. Predictors from bottom to top: Freq (frequency), Orth (orthographic neighborhood density), FAM (familiarity), FS (family size), NCS (number of complex synsets), Phon (phonological neighborhood density), NSS (number of simplex synsets), DE (derivational entropy), REG (regularity), Cons (consistency), FID (frequency initial diphone), FE (friends-enemies measure), Body (body neighborhood density), BG (summed bigram frequency), BGM (mean bigram frequency), L (length).

Above, we evaluated whether or not the ndr_a_, drc, cdp+, and cdp++ models capture the effects of lexical predictors. We now take a closer look at the relative effects these lexical predictors in the model simulations. Fig 23 plots the modeled predictor coefficients (*β*s) in the linear regression models for each predictor in the observed data against the coefficients in the naming latencies simulated by the ndr_a_ (top left panel), drc (top right panel), cdp+ (bottom left panel), and cdp++ (bottom right panel). Ideally, the points in these graphs are on a straight line. This would indicate that the relative effect sizes in the simulated data are identical to those in the observed data. The plot for the ndr_a_ deviates very little from this ideal pattern of results. The accuracy of the predictor effect size in the ndr_a_ is confirmed by a correlation of *r* = 0.995 between the coefficients for the observed data and the coefficients in the ndr_a_ simulations.

The effect sizes for the ndr_a_ are larger than those for the observed data. Importantly, this does not imply that the models are overfitting predictor effects. As noted by Adelman and Brown [53], the standard deviation for modeled naming latencies is smaller than that for observed latencies. The reason for this is that models operate under perfect noise-free conditions. This stands in sharp contrast to the observed naming latencies, even when those observed latencies are averaged over participants. We normalized the observed and simulated latencies prior to our simulations. As a consequence, the smaller standard deviation in the simulated data results in larger estimated effect sizes. The increased effect sizes in the ndr_a_ as compared to the observed data, therefore, are a result of the noise-free conditions in the model simulations.

The coefficients in the cdp+ (*r* = 0.972) and cdp++ (*r* = 0.966) simulations are highly correlated with the coefficients for the observed data as well. Nonetheless, the relative effect sizes deviate more from those in the observed data for the cdp+ and cdp++ models than for the ndr_a_. The coefficients for the cdp+ and cdp++ reveal two particular problems with the relative effect sizes for these models. First, the effect sizes of the neighborhood density measures are too similar. Both models overestimate the effect of body neighborhood density and underestimate the effect of orthographic neighborhood density. Second, the effect of regularity is substantially larger than that of consistency. This stands in contrast to the observed data, where both effects are similar in size. These observations indicate that the cdp+ model puts too much importance on processes underlying the effects of body neighborhood density and regularity.

Finally, the correlation between the coefficients in the observed data and the coefficients simulated by the drc model is *r* = 0.506. The drc thus has substantial problems capturing the relative effect sizes for the effects of the lexical-distributional variables. Due to its reliance on explicit grapheme-to-phoneme conversion rules in its sub-lexical route, the drc drastically overestimates the effect size of the effect of regularity. Furthermore, as was the case for the cdp+ and cdp++ models, the simulated effect of body neighborhood density is much stronger than the effect of this predictor in the observed data.

### Overall model fit

#### Item-level performance

Now that we discussed the effects of individual predictors it is time to consider the overall fit of the ndr_a_ model to word naming data. A first issue to address is the item-level performance of the models (see e.g. [61]). The correlation between the observed naming latencies from the elp and the naming latencies simulated by the ndr_a_ for the 2, 510 mono-syllabic mono-morphemic nouns under investigation is *r* = 0.515. For the dual-route models under investigation, the correlation between simulated and observed naming latencies is highest for the cdp+ model: *r* = 0.491. The item-level correlations for the cdp++ (*r* = 0.307) and the drc (*r* = 0.224) are substantially less high.

To further investigate the overall performance of the models, we extracted average response times for all mono-morphemic, mono-syllabic words that can be used as nouns and that are present in the celex lexical database from four more data sets: Balota and Spieler (1998) [100] (averaged over young and old participants), Seidenberg and Waters (1989) [101], Treiman (1995) [102], and Kessler and Treiman (2002) [103]. Next, we calculated item-level correlations for all models for each data set. These item-level correlations are presented in Table 3. Table 3 furthermore presents the results of a meta-analysis, in which we calculated the correlation of the simulated naming latencies with the averaged (normalized) naming latencies for each word across the five studies.

**Table 3:** Item-level correlations. Item-level correlations for the English Lexicon Project, Balota and Spieler (1998), Seidenberg and Waters (1989), Treiman (1995), and Kessler and Treiman (2002) data sets, as well as for a meta-analysis of these data sets.

	NDR_A_	DRC	CDP+	CDP++
English Lexicon Project	0.515	0.224	0.491	0.307
Balota and Spieler (1998)	0.492	0.196	0.461	0.390
Seidenberg and Waters (1989)	0.384	0.175	0.318	0.274
Treiman (1995)	0.372	0.211	0.414	0.335
Kessler and Treiman (2002)	0.413	0.161	0.365	0.233
meta-analysis	0.534	0.224	0.480	0.326

Consistent with the item-level performance for the elp data, the ndr_a_ outperforms the other models for three of the four additional data sets: the Balota and Spieler (1998), Seidenberg and Waters (1989), and Kessler and Treiman (2002) data. For the fourth data set, Treiman (1995), the item-level correlation between simulated and observed naming latencies is somewhat higher for the cdp+ than for the ndr_a_. The meta-analysis confirms the excellent performance of the ndr_a_. The correlation of the naming latencies simulated by the ndr_a_ and the averaged (normalized) response times across the five studies is 0.534.

William’s tests [104] on the correlations in the meta-analysis revealed that the item-level correlation for the ndr_a_ is significantly higher than the item-level correlation for the drc (*r* = 0.224, *t* = 14.361, *p* < 0.001), the cdp+ (*r* = 0.480, *t* = 3.6384, *p* < 0.001), and the cdp++ (*r* = 0.326, *t* = 11.299, *p* < 0.001). Despite its parsimonious single-route architecture, the item-level performance of the ndr_a_ thus exceeds that of state-of-the-art dual-route models.

**Fig 24:**
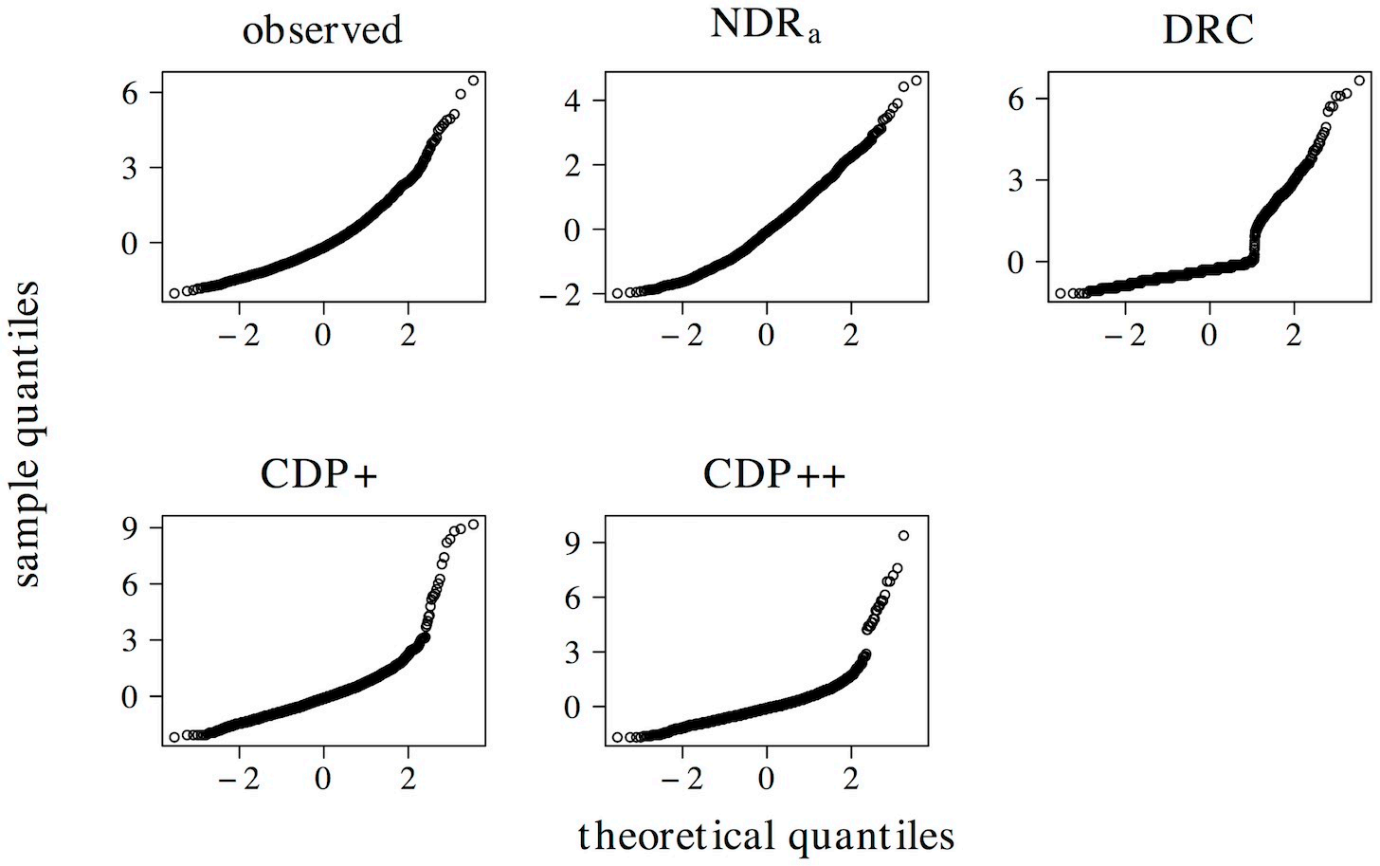
Naming latency distributions. Quantile-quantile plots of the observed naming latencies and the naming latencies simulated by the ndr_a_ and cdp+ models.

The distributions of the observed and simulated naming latencies provide additional insight into the item-level performance of the models. Fig 24 presents quantile-quantile plots of the (non-transformed) observed naming latencies and the naming latencies simulated by the ndr_a_, drc, cdp+, and cdp++. The observed naming latencies and the latencies simulated by the ndr_a_ show a near-normal distribution with a somewhat longer right tail. The distributions of the latencies simulated by the drc, cdp+, and cdp++, however, are far from normal and have very pronounced right tails. This problem is not resolved by applying an inverse or logarithmic transform to the naming latencies simulated by these models.

#### Principal components regression analysis

A second issue regarding the overall model fit is how well the model characterizes the multidimensional structure described by the predictors under investigation. Above, we established the effect of each predictor in isolation. To some extent, this allowed us to get away from the multicollinearity issue. The effects of predictors in isolation, however, may be confounded with the effects of other predictors. It could be argued, for instance, that the ndr_a_, drc, cdp+, and cdp++ models should not be sensitive to the effects of the semantic predictors related to the number of synsets in WordNet. The number of of simplex synsets (*r* = 0.534) and the number of complex synsets (*r* = 0.572) have medium strength correlations with word frequency. The possibility exists, therefore, that the reported effects of these semantic predictors are artefacts of their statistical relation with word frequency and/or other predictors. Indeed, both measures fail to reach significance in a linear regression model that includes all 16 predictors (number of simplex synsets: *t* = −1.556, *p* = 0.120; number of complex synsets: *t* = −1.269, *p* = 0.204).

We therefore sought to verify that the overall characterization of the multidimensional predictor space by the ndr_a_ is correct. To ascertain that the effects of predictors are accurately capture not only in isolation, but also when the values of other the predictors are taken into account, we carried out a principal components analysis on the 16-dimensional space described by the predictors length, orthographic neighborhood density, phonological neighborhood density, body neighborhood density, regularity, consistency, friends-enemies, frequency, familiarity, mean bigram frequency, summed bigram frequency, frequency initial diphone, number of simplex synsets, number of complex synsets, morphological family size, and derivational entropy. This principal components analysis serves as a litmus test for the extent to which the models capture the influence of the overall organisation of lexical-distribution space on the word naming latencies. Table 4 presents the loading of the predictors on the first 8 principal components. Together, these eight principal components explained 86% of the variance in the input space. PC1 has high loadings for predictors that describe the frequency of a word (family size: 0.40, frequency: 0.39, familiarity: 0.36), whereas PC2 contrasts word length (-0.43) with neighborhood density (orthographic neighborhood density: 0.34).

**Table 4:** Results of a principal components analysis on the 16 dimensional space described by the predictors. Listed are predictor loadings for the first 8 principal components.

	PC1	PC2	PC3	PC4	PC5	PC6	PC7	PC8
Length	-0.23	-0.43	-0.17	-0.05	-0.02	0.11	0.21	0.05
Orthographic N	0.27	0.34	0.00	0.35	0.04	0.21	0.01	-0.10
Phonological N	0.21	0.23	0.20	0.48	-0.04	0.11	0.30	-0.01
Body N	0.18	0.23	-0.40	0.13	-0.03	0.33	0.22	0.09
Regularity	0.07	0.17	-0.29	-0.05	-0.14	-0.74	0.53	-0.02
Consistency	0.14	0.07	-0.49	-0.12	0.08	-0.03	-0.38	-0.07
Friends-Enemies	0.11	0.14	-0.56	-0.09	0.06	0.06	-0.17	-0.01
Frequency	0.39	-0.18	0.08	-0.03	-0.31	-0.03	-0.13	-0.32
Familiarity	0.36	-0.20	0.05	-0.09	-0.36	-0.05	-0.10	-0.43
Mean Bigram Frequency	-0.06	-0.35	-0.25	0.37	0.00	0.12	0.21	-0.30
Summed Bigram Frequency	-0.00	-0.00	-0.00	-0.00	-0.00	-0.00	-0.00	-0.00
Frequency initial diphone	0.10	-0.13	0.00	0.56	0.10	-0.45	-0.48	0.30
Simplex synsets	0.32	-0.19	-0.06	-0.11	-0.23	0.18	0.13	0.65
Complex synsets	0.35	-0.18	0.09	-0.09	-0.04	-0.05	0.05	0.26
Family Size	0.40	-0.21	0.06	-0.12	0.30	0.00	0.09	0.01
Derivational Entropy	0.26	-0.11	0.06	-0.11	0.76	-0.06	0.15	-0.16

The results of a linear regression model fit to the first eight principal components are shown in Table 5. The ndr_a_ predicts the right sign for all principal components. Consistent with the effect sizes for the predictors themselves, the effect sizes of the principal components are larger for naming latencies simulated by the ndr_a_ than for the observed data. Again, however, the relative magnitude of the effect sizes (*β*s) is highly similar for the simulated and observed data (*r* = 0.942). This demonstrates that the ndr_a_ simulations capture the overall input space quite well.

**Table 5:** Results of a principal components analysis on the 16 dimensional space described by the predictors. Listed are *β* coefficients for the first 8 principal components.

	observed	NDR_A_	DRC	CDP+	CDP++
PC1	−0.235	−0.342	−0.134	−0.304	−0.205
PC2	−0.070	−0.152	−0.105	−0.026	−0.039
PC3	−0.021	−0.158	0.212	0.028	0.006
PC4	0.001	0.072	0.026	0.000	0.012
PC5	0.051	0.147	0.128	0.165	0.125
PC6	0.141	0.158	0.430	0.117	0.109
PC7	0.139	0.260	−0.336	0.021	0.003
PC8	0.077	0.147	0.044	0.095	0.074

The cdp+ and cdp++ do not capture the input space as well as the ndr_a_. This is reflected in a somewhat lower correlation with observed principal components coefficients (*r* = 0.86 for both models). Both models incorrectly predict an inhibitory effects of PC3, which has strong negative loadings for consistency, friends minus enemies, and body neighborhood density. The effect sizes in both models, as well as in the observed data, however, are limited. The correlation between the coefficients simulated by the drc and the observed coefficients is weak (*r* = 0.274). The drc incorrectly predicts a large inhibitory effect of PC3 that is much stronger than the effects of PC3 in the cdp+ and cdp++ models. Furthermore, the model incorrectly predicts a large inhibitory effect of PC7, which has a high positive loading for regularity. The shortcomings of the drc, cdp+, and cdp++ models that emerged in our analysis of the relative predictor effect sizes thus re-surface in the principal components analysis of the data.

### Comparison to a dual-route architecture

The single route architecture of the ndr_a_ model provides a good fit to observed reading aloud data. It could be the case, however, that adding a sub-lexical route would improve the model’s performance. This issue is particularly relevant given the fact that the sub-lexical route of the cdp+ model has a significant contribution in terms of explained variance, both in word and non-word naming [2]. To resolve this issue we implemented a sub-lexical route by means of a Rescorla-Wagner network that learned to associate orthographic input cues (letters and letter bigrams) with phonological outcomes (demi-syllables). We trained this sub-lexical Rescorla-Wagner network on the same set of training data as the ndr_a_. This resulted in three additional model components, describing the activation of the first (*ActPhonSub_1_*) and second (*ActPhonSub_2_*) demi-syllable in the sub-lexical route and the entropy over these activations (*HSub*). We then fitted two linear regression models to the (inverse transformed) observed naming latencies. The first linear model included as predictors the (log-transformed) components of the original ndr_a_ model. The coefficients of this linear model were highly similar to the parameter settings used in the simulations throughout this paper (*r* = 0.996). The second linear model included as predictors not only the components of the ndr_a_ model, but also the 3 additional measures derived from the sub-lexical discrimination learning network.

Table 6 presents the *t*-values associated with each component in the linear model containing the lexical components of the ndr_a_ and the linear model containing both lexical and sub-lexical components. This dual-route model will henceforth be referred to as the ${\text{ndr}}_{A}^{2}$. Table 6 shows that the relative contributions of the lexical components are similar in the ndr_a_ and ${\text{ndr}}_{A}^{2}$. Adding a sub-lexical route to the model architecture does not affect the contribution of the lexical model components much. Neither the activation of the demi-syllables from the orthography, nor the entropy over these activations reaches significance in the linear model for the ${\text{ndr}}_{A}^{2}$. Furthermore, the predicted values of the ndr_a_ and ${\text{ndr}}_{A}^{2}$ linear models are highly similar (*r* = 0.998), and both models show similar correlations with the observed naming latencies (ndr_a_: *r* = 0.500; ${\text{ndr}}_{A}^{2}$: *r* = 0.501).

**Table 6:** Results of a linear model predicting observed reaction times from model components. Listed values are component *t*-values.

	NDR_A_	${\text{NDR}}_{\text{A}}^{2}$
lexical route		
*ActLexeme*	5.256	3.425
*ActPhon_1_*	4.123	4.015
*ActPhon_2_*	11.019	10.580
*H*	7.666	7.371
*Complexity*	16.607	15.703
sub-lexical route		
*ActPhonSub_1_*	NA	1.458
*ActPhonSub_2_*	NA	0.203
*HSub*	NA	1.113

The results for the linear models presented here demonstrate that the addition of a sub-lexical route does not improve the performance of the ndr_a_ in word naming. In addition, the simulations for the individual predictors demonstrated that the effects documented in the non-word naming literature are adequately captured by the single lexical route architecture of the ndr_a_. The current simulations therefore suggest that a single-route architecture is sufficient to capture the patterns of results observed in the response times in both word and non-word naming experiments.

### Non-word frequency effect

A reanalysis of the McCann and Besner [58] naming latencies for non-words sheds interesting new light on the use of a lexical architecture for non-word naming. For each of the 154 non-words in the study, both standard non-words and pseudo-homophones, we obtained unigram frequencies from the Google 1T *n*-gram corpus [48]. The unigram frequency list from the Google 1T *n*-gram corpus includes words with a frequency of 200 or greater only. It is striking therefore, that only 15 of the 154 non-words did not appear in the Google unigram corpus. A Google web search for these 15 words showed that even the least frequent of these words still appeared on 7, 700 web pages. Furthermore, the average Google unigram frequency of non-words (184, 396) is comparable to that of low frequency English words like *matriculation* (frequency: 183, 617) or *mannequin* (frequency: 184, 551). This suggests that from a distributional perspective, the distinction between words and non-words is not as absolute as is commonly believed. As for real words, any given non-word therefore may or may not have a representation in the mental lexicon of an individual language user. The probability of such a representation existing is a function of the frequency of the word or non-word.

Given these observations we investigated whether there was a frequency effect of non-words in the naming latencies for Experiment 1 in McCann and Besner [58]. We found a highly significant effect of non-word frequency (*t* = −5.838, *β* = −0.428). This effect of non-word frequency existed over and above the effects of word length, orthographic neighborhood density, base word frequency and non-word type (regular or pseudo-homophone). Non-word frequency was the most powerful predictor of non-word naming latencies and showed a correlation to observed naming latencies (*r* = −0.428) similar to that of the word frequency measure in the elp naming latencies for real words (*r* = 0.451).

To verify that the architecture of the ndr_a_ supports non-word frequency effects, we retrained the model on input data that, in addition to the original input data the ndr_a_ was trained on, contained the non-words from the McCann and Besner [58] study with their Google unigram frequency. With parameter settings identical to those in all previously reported simulations, this model correctly simulates the non-word frequency effect (*t* = −8.056, *β* = −0.547). As expected, the cdp+ (*t* = −1.323, *β* = −0.107) and cdp++ (*t* = −0.852, *β* = −0.069) model do not capture this effect. We do expect the cdp+ and cdp++ models to capture the non-word frequency effect if the training data for these models were enriched in a similar fashion as the training data for the ndr_a_ model. Surprisingly, the drc does predict a significant effect of non-word frequency (*t* = −3.459, *β* = −0.270) without adjustments to its input data, presumably due to the strong correlation between non-word length and non-word frequency (*r* = −0.429).

**Fig 25:**
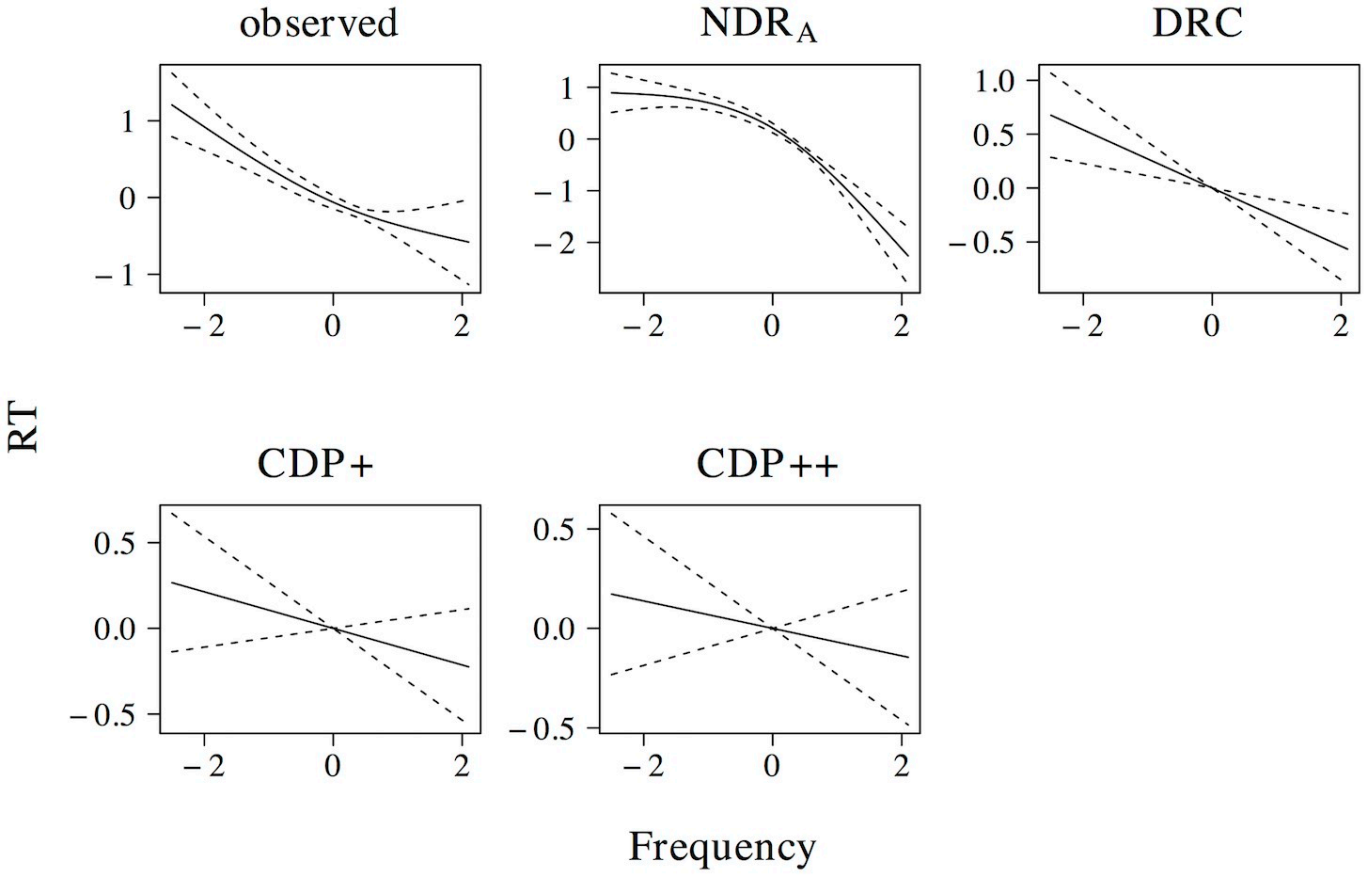
Frequency: Non-words. The effect of frequency in non-word naming.

The results of a non-linear model for non-word frequency are presented in Fig 25. The observed naming latencies show a facilitatory effect that levels off for the highest frequency non-words. The ndr_a_ captures the facilitatory trend, but predicts that the effect levels off for the 15 non-words that did not appear in the Google unigram corpus, rather than for the highest frequency non-words. Given the limited size of the current set of non-words, we are hesitant to draw strong conclusions on the basis of this discrepancy. If future research were to indicate that the observed effect is robust and that the ndr_a_ systematically underestimates its non-linearity, we hypothesize that revised, more carefully selected training data might lead to better simulation results. The latencies simulated by the drc reveal a linear effect of non-word frequency, while the cdp+ and cdp++ show a non-significant trend towards facilitation.

The non-word frequency effect suggests that the dichotomous distinction between words and non-words is perhaps better thought of as a difference on a gradient scale, with high frequency words on one end of the scale and low frequency words on the other. Conceptually, such a gradient scale fits well with the architecture of the ndr_a_, in which the difference between word and non-word processing is quantitative rather than qualitative in nature: words and non-words are processed by the same cognitive architecture, with differences only in the amount of activation flowing through the system.

We conclude this section on a note about the quantitative performance of the ndr_a_ and cdp+ models for the McCann and Besner [58] naming latencies. The predicted naming latencies from the reading aloud models correlate poorly with the observed naming latencies (ndr_a_: *r* = 0.131; drc: *r* = 0.107; cdp+: *r* = 0.078; cdp++: *r* = 0.087). One potential explanation for the poor quantitative performance of the models may be the fact that the stimulus list of Experiment 1 of McCann and Besner [58] consisted of non-words only. Task strategies, therefore, may substantially differ from experiments in which mixed stimulus lists are used. Alternatively, the visual input interpretation mechanism used in the current implementation of the ndr_a_ may be too simplistic. The predicted values of a simple linear model including a frequency-weighted version of the visual complexity measure (*Complexity*/(*LogFrequency* + back-off constant)) rather than the original complexity measure boosted the correlation with the observed non-word naming latencies to *r* = 0.461, a correlation close to the correlation between the word naming latencies in the elp and the word naming latencies simulated by the ndr_a_. We return to the issue of familiarity with the visual input in the discussion section below.

### Pronunciation performance

The discrimination learning core of the ndr_a_ models response times in the reading aloud task, and does not generate actual pronunciations of words or non-words. The reason for this is that we believe that there is a functional separation between the processes by which responses are learned and response conflict resolution. The core of the ndr_a_ models the processes by which responses are learned. To be able to generate correct pronunciations for words and non-words, however, language users must resolve conflicts that arise during the selection of a response. As noted by Novick et al. [39], the pre-frontal cortex (pfc) plays a crucial role in response conflict resolution. The processes by which responses are learned are relatively well understood. By comparison, our understanding of the functional architecture of the pfc is limited. As a consequence, considerable uncertainty remains with respect to the optimal implementation of a verification mechanism. Here, we adopt a crude approximation of what we think the architecture of a checking mechanism might look like. We evaluate the pronunciation performance of the ndr_a_ when this checking mechanism is added to the discrimination learning core of the ndr_a_.

#### Response conflict resolution in the NDR_A_

The basic rationale behind the checking mechanisms adopted here is that the pfc filters the set of lexical representations that activate demi-syllables to only include the subset of lexemes that share orthographic features with the target word or non-word. As such, the checking mechanism used here limits response conflict monitoring to the lexical level: the pre-frontal cortex monitors the set of activated lexemes and removes from this set of activated lexemes those lexical representations that are inappropriate given the orthographic input. No further response monitoring takes place at the phonological level.

Our implementation of the checking mechanism builds on the idea that a lexeme points to letters and letter order information (which is required, for instance, for writing). This information is then compared on-line against the orthographic features in the input. As pointed out earlier, the assumption that language users are able to compare the orthographic features associated with a lexeme to the orthographic features in the input is not unique to the verification mechanism proposed here. Instead, it is a general assumption of discrimination learning that is necessary to evaluate whether or not the outcome of a learning event is predicted correctly, and that is consistent with theories of cortical processing that propose a bi-directional pass of information between higher and lower levels of information [35].

How exactly does the checking mechanism work? Consider the example word *bear*. When the orthographic string *bear* is presented on the screen, activation spreads to a large number of lexical representations. The set of activated lexemes includes orthographic neighbors of *BEAR* such as *PEAR*, *HEAR* and *FEAR* as well as the target lexeme *BEAR* itself. For a correct pronunciation of the word *bear*, however, it is sufficient to consider only those demi-syllables that are activated by the target lexeme *BEAR*. The checking mechanism therefore limits activation of demi-syllables to those units that are activated by this target word lexeme. In the case of *bear*, the initial demi-syllable that receives most activation from the lexeme *BEAR* is *b8*, whereas the most active second demi-syllable is *8R*. The model therefore correctly pronounces the word *bear* as *b8R*.

For a vast majority of all words, the most active word-initial and word-final demi-syllables are compatible in the sense that the vowel in the initial and final demi-syllables is identical. For 10 out of the 2, 510 monosyllabic words in our data set, however, the vowel in the most active first and second demi-syllable are different. In these cases the checking mechanism gives preference to the vowel in the second demi-syllable. This implementational decision corresponds to the fact that the activation of the second demi-syllable has a somewhat higher weight in the ndr_a_ as compared to the activation of the first demi-syllable (weight *ActPhon_1_*: 0.050, weight *ActPhon_2_*: 0.098) and to the increased perceptual prominence of rhymes as compared to onset plus vowel sequences.

For a non-word such as *bap* no lexical representation exists. Limiting the phonological units that influence pronunciation to those activated by the target word lexeme therefore does not work for non-words. Instead, the checking mechanism needs to identify which lexical representations share relevant orthographic features with the non-word *bap*. Only the phonological activation generated by these lexemes should influence non-word pronunciation. The question then becomes how to define the term “relevant orthographic features”. One option is to include all lexemes whose orthographic representations share at least *n* orthographic bigrams with the non-word presented on the screen. The problem with such a definition is that the checking mechanism would be relatively insensitive to the serial nature of the non-word naming task.

We propose an alternative definition that takes into account the left-to-right nature of reading and speech production by varying the set of lexemes that influence pronunciation in a serial manner. For the first demi-syllable, the checking mechanism proposed here ensures that the initial demi-syllables considered for pronunciation are restricted to the set of initial demi-syllables that receive activation from lexemes that share the orthographic onset and vowel with the presented non-word. For the non-word *bap*, for instance, only those initial demi-syllables that are activated by one of the 28 lexemes that share the orthographic onset and vowel *ba* with the lexeme *BAP* are considered for pronunciation (e.g., *BACK*, *BALM*, *BALL*, …).

For the second demi-syllable, the checking mechanism considers the combination of the orthographic vowel and coda when selecting the appropriate second demi-syllable (i.e., it limits the set of word-final demi-syllables considered for pronunciation to the set of word-final demi-syllables that receives activation from lexemes that share the orthographic rhyme with the presented non-word). For the non-word *bap*, for instance, only those word-final demi-syllables that are activated by one of the 21 lexemes that share the orthographic rhyme *ap* with the lexeme *BAP* are considered for pronunciation (e.g., *CHAP*, *LAP*, *SWAP*, …). The checking mechanism proposed here assumes that the system is sensitive to the distinction between vowels and consonants. A similar assumption is made in the sub-lexical route of the cdp+ model, which parses the visual input into consonant and vowel slots in a grapheme buffer.

Consistent with the architecture of the ndr_a_, we weighted the contribution of lexical representations to demi-syllable activations for the amount of activation they received from the orthographic features of the non-word (see Eq 7). For the non-word *bap* the initial demi-syllable that received the highest activation from the co-activated lexical representations was *b*{, whereas the highest activated second demi-syllable was {*p*. Together, these demi-syllables yield the correct pronunciation of the non-word *bap*, which is *b*{*p*. The same procedure was used to resolve ties for existing words for which the activation of a demi-syllable from the target word lexeme was equally high for two or more demi-syllables (i.e., for 99 word-initial demi-syllables (3.94%) and 184 word-final demi-syllables (9.12%)).

For a vast majority of the 2, 510 words and 1, 784 non-words under consideration, the algorithm described above yields a single most highly activated first and second demi-syllable. For 101 words (4.02%) and 81 non-words (4.54%), however, two or more potential word-initial or word-final demi-syllables still receive equal activation. For these non-words the checking mechanism resorts to the phonological activations generated by the set of lexical representations that share the orthographic onset (rather than onset plus vowel) or the orthographic coda (rather than rhyme) with the word or non-word to resolve the tie, considering only those word-initial or word-final demi-syllables that share the phonological coda with one of the demi-syllables involved in the tie.

#### Simulation results

The ndr_a_ model generates correct pronunciations for 2, 493 of the 2, 510 monosyllabic words in our database, resulting in a word pronunciation performance of 99.32%. A majority of the pronunciation errors (10 out of 17) concerns words that have more than one pronunciation in the celex lexical database, such as *tear* or *wind*. For these words the model chooses the more frequent pronunciations *t8R* and *wInd* over the less frequent pronunciations *t7R* and *w2nd*, as would participants in a reading aloud task. Of the remaining 7 erroneous pronunciations in the ndr_a_, 3 were based on position-specific grapheme-to-phoneme conversions that exist in other English words: *blouse* is pronounced as *bl6s* rather than *bl6z* (analogous to *house* (*h6s*)), *draught* as *dr#t* rather than *dr#ft* (analogous to *fraught* (*fr$t*)), and *vase* is pronounced as *v#s* rather than *v#z* (analogous to *case* (*k1s*)). The remaining erroneous pronunciations contain grapheme-to-phoneme conversions that are not attested in English: the ndr_a_ pronounces *year* as *j7d* rather than *j7R*, *font* as *bQnt* rather than *fQnt*, *beige* as *kw1Z* rather than *b1Z*, and *sky* as *sk2b* rather than *sk2*.

Comparing the pronunciation performance of the ndr_a_ model to that of the other models is not entirely straightforward. Consider, for instance, the cdp+ model. While the sub-lexical route of the cdp+ model was trained on the British pronunciations in the celex lexical database, the training data for the interactive activation model in the lexical route are not explicitly specified in Perry et al. [2]. The pronunciations of the cdp+ model, however, suggest that the lexical route was trained on a variety of American English, rather than British English. Consequently, the cdp+ model typically pronounces the vowel *$* as *9*. Provided that these types of pronunciations are likely to reflect differences in training data rather than differences in model performance, we decided to not consider such pronunciations erroneous.

After correcting for differences in the training data, the drc generates correct pronunciations for 2, 493 of the 2, 510 words under investigation, for a naming performance of 99.28 (11 words with multiple pronunciations, 6 grapheme-to-phoneme conversions that exist in English, 1 grapheme-to-phoneme conversion that does not exist in English). Both the cdp+ (2, 472 correct pronunciations (98.49%); 11 words with multiple pronunciations, 18 grapheme-to-phoneme conversions that exist in English, 9 grapheme-to-phoneme conversions that do not exist in English) and the cdp++ (2, 491 correct pronunciations (99.24%); 9 words with multiple pronunciations; 4 grapheme-to-phoneme conversions that exist in English; 6 grapheme-to-phoneme conversions that do not exist in English) show excellent pronunciation performance as well.

All models thus accurately pronounce real words. Word pronunciations, however, are generated by lexical architectures both in the single-route ndr_a_ model and in the dual-route drc, cdp+, and cdp++ models. By contrast, for non-words, the dual-route models directly map orthographic units onto phonological units in their sub-lexical routes, whereas the ndr_a_ relies on co-activation of orthographic neighbors in a lexical architecture. Much more than word pronunciation, therefore, non-word pronunciation provides a litmus test for the combination of the single-route lexical architecture of the discrimination learning core of the ndr_a_ model with a top-down checking mechanism.

**Fig 26:**
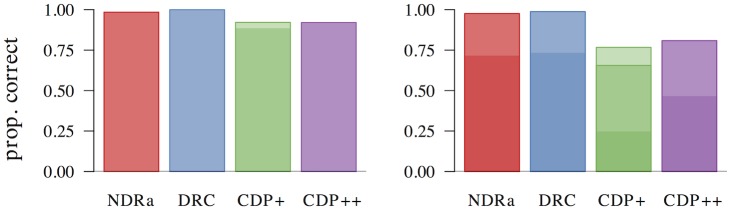
Pronunciation performance: Non-words. Non-word pronunciation performance for the nonwords from the ARC non-word database (left panel) and for the Pritchard et al. (2012) data (right panel). Lighter shaded green areas in both panels indicate additional pronunciation accuracy in the cdp+ model when the naming activation criterion parameter is changed from 0.67 to 0.50.

We report two analyses of non-word pronunciation performance. First, we establish the pronunciation performance of the models for the set of 1, 784 non-words discussed throughout this paper. Next, we compare the pronunciations of the models to the pronunciations of participants in the non-word reading study by Pritchard (2012) [105]. The performance of the models for both data sets after correcting for differences in the training data is presented in Fig 26.

Following Perry et al. [2] we adopt a lenient error scoring criterion for the pronunciation performance on the non-words from the arc non-word database. This lenient scoring criterion is similar to the scoring criterion proposed by Seidenberg et al. [84], according to which a non-word pronunciation is correct if it is based on grapheme-to-phoneme conversions that exist in real English words. The lenient scoring criterion used here, however, is a bit stricter than that proposed by Seidenberg et al. [84], in the sense that we considered non-word pronunciations as correct if and only if the orthography-to-phonology mapping for the onset, vowel and coda existed for a monosyllabic word in celex.

Using the lenient scoring criterion, the ndr_a_ mispronounces 27 non-words, for a non-word pronunciation performance of 98.49%. Out of these 27 erroneous responses, 18 concern mispronunciations of the onset (e.g., *rhewn* pronounced as *wun* rather than *run*), 6 concern mispronunciations of the vowel (e.g., *phelte* pronounced as *fQlt* rather than *fElt*), and 3 concern mispronunciations of the coda (e.g., *dred* pronounced as *drEv* rather than *drEd*). Despite this excellent performance, the ndr_a_ is outperformed by the drc, which mispronounces a single non-word only (*rhewn* pronounced as *rjun* rather than *run*). The drc thus reaches a pronunciation performance of 99.94% for the non-words in the arc database.

The pronunciation performance of the cdp+ and cdp++ models is 88.51% (205 mispronunciations) and 92.04% (142 mispronunciations), respectively. Consistent with the observations of Perry et al. [2], a large percentage of the pronunciation errors of the cdp+ model displayed the pattern that a phoneme was missing from the pronunciation. Perry et al. [2] state that reducing the naming activation criterion (i.e., the threshold activation for pronouncing a phoneme) from 0.67 to 0.50 substantially reduces the number of erroneous pronunciations in the model. Indeed, changing the naming activation criterion parameter from 0.67 to 0.50 results in correct pronunciations for 65 out of the 205 mispronounced non-words. This boosts the pronunciation performance of the cdp+ model to 92.15% (light shaded area in the left panel of Fig 26).

The Pritchard et al. data contain pronunciations of 412 non-words by 45 participants. We evaluated the pronunciation performance of the models for these data in two ways. First, we established the most common pronunciation for each non-word across participants. We henceforth refer to this pronunciation as the main pronunciation of a non-word. We then encoded whether or not a model pronunciation was identical to the main pronunciation. Second, we verified whether or not a model pronunciation coincided with the pronunciation of *any* participant. Fig 26 shows the results of this two-step evaluation. The darker shaded bars indicate the proportion of model pronunciations that were identical to the main pronunciation. The medium shaded bars represent the proportion of model pronunciations that were identical to the pronunciation of at least one participant.

As was the case for the non-words from the arc database, the drc model shows the best pronunciation performance. For 302 of the 412 non-words in the Pritchard et al. data (73.30%) the pronunciation of the drc is the main pronunciation. A further 105 (25.48%) pronunciations of the drc were produced by at least one participant. No more than 5 (1.21%) pronunciations generated by the drc model were not produced by any participant. The pronunciation performance of the drc for the Pritchard et al. data hence is 98.79%. The performance of the ndr_a_ is not much worse than that of the drc. The pronunciations of the ndr_a_ coincide with the main pronunciation for 295 words (71.60%), and a further 107 (25.97%) pronunciations generated by the ndr_a_ were produced by at least one participant. This leaves 10 (2.43%) pronunciations that were not produced by a participant, for a pronunciation performance of 97.57%.

The cdp+ model produces the main pronunciation for 102 non-words (24.76%), and a pronunciation that was produced by at least one participant for another 168 (40.78%) non-words. Adjusting the naming activation criterion parameter from 0.67 to 0.50 yields pronunciations that were produced by at least one participant for another 46 non-words (11.17%). Nonetheless, 96 (23.30%) pronunciations generated by the cdp+ model were not pronounced by a participant. The performance of the cdp+ model therefore is 76.70%. The pronunciation performance of the cdp++ model is somewhat better than that of its predecessor: 192 (46.60%) of its pronunciations are the main pronunciation, and a further 141 (34.22%) are produced by at least one participant. With 79 (19.17%) pronunciations that were not produced by a participant, the pronunciation performance of the cdp++ model for the Pritchard et al. (2012) data therefore is 80.83%.

In summary, the pronunciation performance for words is excellent for all models. For non-words, the ndr_a_ performs slightly worse than the drc, but substantially better than the cdp+ and cdp++. We expect that the pronunciation of the cdp model would improve with richer training data that contain all orthography-to-phonology mappings that are relevant for the correct pronunciation of the non-words under investigation. The evaluation of the pronunciation performance of the ndr_a_ for words and non-words reveals that the single-route architecture of the ndr_a_ allows for competitive pronunciation performance for both words and non-words when a checking mechanism is added onto the discrimination learning core of the model.

## Discussion

### Single-route architecture

The use of a single, rather than a dual-route architecture is a key aspect of the work reported here. The drc [1], cdp [2, 5, 9] and triangle models [4, 7, 8] all are dual-route models of reading aloud. Here, we presented a new single-route model of response times in the reading aloud task that is based on the equilibrium equations [30] for the learning algorithm of Rescorla and Wagner [28]. We demonstrated that this single route model replicates a wide range of predictor effects that have been documented in the experimental literature, both in isolation and in a principal components analysis that captures the joint effects of predictors on observed naming latencies. Furthermore, the model achieves an overall fit to the data comparable to or better than that of state-of-the-art dual-route models. Furthermore, we showed that adding a sub-lexical route to the model did not improve its performance.

While the single versus dual route debate remains as open in the neuroscience literature as it is in the functional level linguistics and cognitive science literature, the single-route architecture of the ndr_a_ is consistent with the results of a large number of studies in the neuroscience literature. These studies found activation of the same brain regions in word and non-word reading, with no unique brain regions that are active in non-word reading only (see [106–109]; cf. [110] for examples of conflicting evidence; see also [111]). Instead, differences in the timing [107, 112, 113] and intensity [107] of the activation of the *same* brain regions were observed between word and non-word reading. As noted by Wilson et al., [107] (p. 1), for instance, “relative to words, pseudo-words elicit more robust activation in the left inferior temporal gyrus (itg, see e.g. [114–117]) and the left inferior frontal gyrus (ifg, see e.g. [109, 115–121])”. As pointed out by an anonymous reviewer, however, it is important to note that a lack of brain regions that are active in non-word reading only does not constitute evidence against a dual-route architecture. The brain has no a priori information about the nature of a stimulus (i.e., word or non-word). Hence, the default strategy in a dual-route approach may well be to activate both routes in parallel.

Despite these successes, there are some outstanding issues that warrant further discussion. First, the ndr_a_ assumes that processing is strictly parallel, while a number of experimental findings suggest that at least some serial processing occurs when preparing to read aloud words and non-words. Second, we made decisions regarding the grain size of representations at both the orthographic (letters and letter bigrams) and the phonological (demi-syllables) level that proved adequate for the current purposes but that are likely to be an oversimplification of more complex neural structures. Third, the ndr_a_ assumes that consistency and regularity effects arise in a single-route lexical architecture. This stands in contrast to traditional theories that assume the necessity of a sub-lexical route to simulate these effects. Fourth, the leading dual-route model uses an interactive activation network in its lexical route, whereas the lexical architecture of the ndr_a_ operates on the basis of discriminative learning principles. In what follows, we discuss each of these topics in more detail.

### Serial versus parallel processing

The serial or non-serial nature of processing has been a central debate in the reading aloud literature (see [1]). Two types of experimental results are typically interpreted as evidence for serial processing. First, Weekes [59] found a length by lexicality interaction, with a stronger effect of length in non-word reading than in word reading. Second, a number of studies [18, 88, 89] found a position of irregularity effect with larger processing costs when grapheme-to-phoneme irregularities occurred in early positions (e.g., *chef*) than when irregularities occurred in later positions (e.g., *blind*). These results have been taken as evidence for a dual-route architecture. In the dual route architectures of the cdp and drc models the sub-lexical route operates in a serial manner: the uptake of orthographic information occurs in a letter-by-letter fashion. The serial nature of the sub-lexical route is conceptually linked to a left-to-right moving window of spatial attention [2, 122]. By contrast, the lexical route of the cdp models processes the entire orthographic input at once and is therefore parallel in nature. In this framework, the interaction of length with lexicality results from the fact that non-word naming exclusively involves the serial sub-lexical route, whereas word naming also involves the parallel lexical route. In non-word naming additional letters lead to additional stages of information uptake and therefore longer naming latencies. This effect is diminished in word naming, because the parallel lexical route is insensitive to differences in word length [2].

Alternatively, length effects may be peripheral to the task of reading aloud and arise from extra-linguistic sources, such as processes related to articulation [2, 123] or visual input decoding. In its current implementation, length effects in the ndr_a_ arise primarily as a result of visual input interpretation, which is consistent with an extra-linguistic interpretation of these effects. Nonetheless, the ndr_a_ correctly predicts that the length effect should be larger for words as compared to non-words. As noted by Perry et al. [2], a potential source for the length by lexicality interaction in parallel models is dispersion. Non-words tend to have less common orthographic and phonological bigrams than real words. The larger length effect for non-words may therefore be a product of the increased likelihood of encountering a low frequency orthographic or phonological bigram in longer non-words. When a low frequency orthographic bigram occurs in a word, less activation is spread to orthographic neighbors, whereas when it contains a low frequency phonological bigram the activated neighbors will send less activation to the target demi-syllables. As such, low frequency orthographic and phonological bigrams both result in longer naming latencies.

While we believe that the length effect in word naming is at least partially driven by extra-linguistic processes, the non-serial nature of the ndr_a_ in its current form does not reflect a conceptual preference in the serial versus parallel processing debate. Indeed, the inability of the current implementation of the ndr_a_ to simulate the position of irregularity effect suggests that a serial uptake of information may be beneficial to the performance of the ndr_a_ model. In a serial implementation, the position of irregularity effect would follow naturally from the increased availability of earlier orthographic input and phonological output units. Furthermore, Perry et al. [2] demonstrated that the serialization of their sub-lexical route boosted item-level correlations significantly. Sensitivity to the serial nature of the reading process also proved pivotal in the implementation of a verification mechanism for the pronunciation performance simulations.

### Visual input interpretation

In the ndr_a_, estimations of the time it takes to interpret the visual input are based on a rudimentary measure of the complexity of the visual input. When we developed the model we considered visual input interpretation peripheral to the linguistic core of the model and primarily implemented it as a convenient analogy to the feature detection systems in the drc and cdp models. In our simulations, however, it became clear that the correct simulation of the length effect in the ndr_a_ depends on the interpretation of the visual input. Given the importance of the length effect in the reading aloud literature, some further thought about the issue is warranted.

In its current form the visual input interpretation mechanism is insensitive to differences between words and non-words. Words and non-words alike are decomposed into letters and letter bigrams, which in turn activate lexical representations. Evidence from the neuroscience literature, however, suggests that the early visual processing in occipital brain regions varies not only as a function of word length [106, 124, 125], but also as a function of lexicality (e.g. [117, 119], cf. [106, 126] for studies that did not find lexicality-related differences of visual occipital region activations). Importantly, the visual occipital system is insensitive to linguistic properties of the input, which suggests that the observed effects of lexicality in this region reflect a difference in familiarity with the visual input between words and non-words.

A post-hoc analysis of the observed elp naming latencies revealed that a refinement of the visual input interpretation mechanism in the ndr_a_ that takes into account the familiarity of the visual input at the word level leads to a substantial improvement in item-level correlations. The predicted values of a simple linear model using as predictors the components of the ndr_a_ model, but replacing the complexity measure with a frequency-weighted alternative (i.e., *Complexity* divided by *(log) Frequency* + backoff constant) showed a correlation of *r* = 0.544 to the observed naming latencies. Simply adding this frequency-weighted alternative to the ndr_a_ model, however, led to a poor qualitative performance of the model. Nonetheless, a visual input interpretation mechanism that takes into account the familiarity of the visual input in a more subtle manner may well lead to further improvements in the performance of the ndr_a_ model. Such a visual complexity measure would fit well with the results of familiarization studies with objects and faces, in which greater occipital activation was found for unfamiliar objects and faces [127, 128].

### Orthographic input units

In the current implementation, orthographic representations in the ndr_a_ model are limited to letters and letter bigrams. Evidence from the neuroscience literature, however, suggests that this simple encoding scheme might be an oversimplification of the neurobiological reality of language processing. Vinckier et al. [129] and Dehaene et al. [130], for instance, found that visual word recognition is sensitive to a hierarchy of increasingly complex neuronal detectors, ranging from letters to quadrigrams.

From a discrimination learning perspective the richness of the encoding scheme is an empirical issue. Language users extract those pieces of information from the input that provide valuable cues to the outcome. The current simulation results suggest that an encoding scheme based on letters and letter bigrams is sufficiently rich to capture a wide range of experimental findings in the reading aloud literature. If future experimental work indicates that higher order *n*-grams provide valuable additional information, however, we have no a priori objections against enriching the orthographic encoding scheme of the ndr_a_. One possibility would be to include high frequency, but not low frequency letter *n*-grams as cues. Such a frequency-dependent coding scheme would help address the familiarity of the input issue raised above as well.

### Phonological output representations

As was the case for the orthographic input level, we also made a decision regarding the grain size of representations at the phonological output level of the ndr_a_. At this level we decided to use demi-syllables [32]. The use of demi-syllables, however, is not free of problems. A verification mechanism added on top of the discrimination learning core of the ndr_a_ in its current form, for instance, does not have access to the information required to correctly pronounce non-words that contain non-existent demi-syllables. As an example, the predominant pronunciation of the non-word *filced* is *[fIlst]*, which includes the non-existent demi-syllable *[Ilst]*. Without a corresponding representation in the ndr_a_, a checking mechanism cannot simulate the pronunciation of this demi-syllable.

Demi-syllables offered an easy-to-implement approximation of acoustic gestures that proved adequate for the current purposes. While this approximation worked well in the simulations reported here and shows that phoneme representations are superfluous for modeling reading aloud, we believe that an implementation of acoustic gestures at a finer grain size that more accurately reflects the biological reality of speech production would further improve the performance of the ndr_a_ and help develop an extension of the model to auditory language processing. One option worth exploring in future research is the use of time-sensitive gestural scores as used in articulatory phonology (see, e.g. [131–134]).

### Consistency effects in a lexical architecture

The effects of consistency and regularity have been important benchmark effects for models of reading aloud. The drc model [1] successfully simulates the factorial effect of regularity (see, e.g. [84–87]) through the grapheme-to-phoneme conversion rules in its sub-lexical route. These rules, however, operate in an all-or-none fashion. As a result, the drc model does not capture graded consistency effects [8, 18, 76, 91], which require the activation of not only the most common grapheme to phoneme mappings, but also that of other, less common mappings.

To overcome the difficulties of the drc model, the cdp model uses the tla sub-lexical network [24, 94] in its sub-lexical route. As noted by Perry et al. [2], the tla sub-lexical network is a simple two-layer learning network that operates on the basis of the delta rule [25]. One advantage of learning models over rule-based models is that they allow non-target words to influence the naming process [102]. Consequently, the tla network allows for the successful simulation of graded consistency effects. In the cdp model the successful simulation of consistency effects, therefore, is a result of the associative learning in the sub-lexical route [2].

By contrast, Coltheart et al. [1] suggest that consistency effects might arise in the lexical route as a result of neighborhood characteristics. Perry et al. [2] (p. 276) contest this claim, stating that “such influences are too weak to account for the majority of the consistency effects reported in the literature”. They support this claim by showing that consistency effects are still captured by a purely feedforward version of the cdp+ in which the activation of orthographic neighbors is completely disabled. The fact that a sub-lexical network can generate consistency effects, however, does not provide conclusive evidence for the claim that a lexical network cannot.

To demonstrate this point we implemented a purely sub-lexical version of the ndr_a_, in which orthographic units are mapped directly onto phonological outcomes. This sub-lexical version of the ndr_a_ captures the linear effects of consistency (*t* = −3.661, *β* = −0.080), regularity (*t* = −9.078, *β* = −0.401) and friends minus enemies (*t* = −2.789, *β* = −0.061). The simulations with the original ndr_a_ model, however, showed that all these effects can be captured in a lexical architecture as well. The fact that a sub-lexical network can capture the effects of consistency therefore does not imply that such a sub-lexical network is a necessary component of a model of reading aloud. The necessity for a sub-lexical route in the cdp+ model may not reflect the psychological reality of such a route, but instead display the shortcomings of the interaction activation model [20] that underlies the lexical route of the cdp model. We return to this issue in the next section.

In the lexical architecture of the ndr_a_, regularity and consistency effects arise due to the co-activation of lexical items with similar orthographies. The co-activated lexical representations of consistent/regular words help co-activate the target word phonology, whereas the co-activated lexical representations of inconsistent/irregular words activate non-target phonological features. As a result, co-activated words help target word naming if and only if their orthography to phonology mapping is consistent with the orthography to phonology mapping for the target word. In line with the suggestions of Coltheart et al. [1], consistency effects in the ndr_a_ therefore arise through neighborhood characteristics. These neighborhood characteristics did not only prove sufficient to simulate the observed effects of regularity and consistency in isolation, but also captured the complex interplay of these predictors as well as the interaction of consistency with friend-enemy measures [91, 95] and frequency [84–87]. Furthermore, the ndr_a_ captures the graded consistency effect for non-words [90, 92]. As such, the ndr_a_ correctly simulates the complex and challenging pattern of results for various orthography-to-phonology consistency measures through a purely lexical architecture.

### Learning

The cdp model is a hybrid model that was built from a nested modeling perspective. The idea behind nested modeling is that a new model should be based on its predecessors [135]. Perry et al. [2] therefore evaluated the strengths and weaknesses of the different components of the drc and the cdp models. They found the rule-based sub-lexical route of the drc model to be suboptimal and replaced it with the learning network of the cdp model [9]. On the other hand, the lexical route of the cdp model was not fully implemented and based on a simple frequency-weighted activation of a lexical phonology [94]. The lexical route of the cdp model was therefore replaced with the interactive activation network of the drc model [1, 20].

While we see the merit of a nested modeling approach, we are less convinced about the hybrid nature of the cdp model that resulted from it. Even if a dual-route model were conceptually correct one would expect that the lexical and sub-lexical route operate on the basis of similar neuro-computational mechanisms. The implementation of the lexical route of the cdp model seems particularly implausible given the fact that interactive activation models avoid the issue of learning (see, e.g. [6]). Perry et al. [2] (p. 303-304) acknowledge this problem and consider the lack of learning in the lexical route one of the limitations of the cdp+ model. In addition, Perry et al. [2] state, the interactive activation model has been shown to fail to account for a number of findings in the lexical decision literature (see, e.g. [22, 23]). We therefore believe that a learning network implementation of the lexical route of the cdp model would be an option worth exploring.

A learning implementation of the lexical route would help establish the necessity for a dual-route architecture in the cdp model. In the current implementation of the cdp model the sub-lexical route has a substantial independent contribution [2]. This independent contribution, however, could have two sources. First, it could reflect the correctness of a dual-route architecture in which both routes reflect different parts of the language processing that occurs in the reading aloud task. Alternatively, however, the independent contribution of the sub-lexical route of the cdp model could be a result of the suboptimal performance of the interactive activation model in its lexical route. In this case, the variance that is currently explained by the sub-lexical route of the cdp could also be explained by a better optimized lexical route. The finding that the addition of a sub-lexical learning network did not improve the performance of the ndr_a_ is consistent with such an interpretation.

## Conclusions

We presented the ndr_a_, a single-route model of response times in the reading aloud task based on the fundamental principles of discriminative learning. The ndr_a_ is an extension of the ndr model by Baayen et al. [6] for silent reading. We showed that the ndr_a_ provides a good overall fit to observed naming latencies. Through the use of generalized additive models we also demonstrated that the ndr_a_ successfully simulates not only the linear, but also the non-linear characteristics of a wide range of predictor effects and interactions documented in the experimental literature. A principal components analysis of the data furthermore indicated that the ndr_a_ captures the overall influence of the structure of lexical-distributional space. As such, the ndr_a_ provides an alternative to leading models of reading aloud, such as the drc [1], cdp+ [2], and cdp++ [5] models.

The ndr_a_ model is a major advancement over existing models of reading aloud in two ways. First, the computational engine of the ndr_a_ is based on the well-established learning algorithm provided by the Rescorla-Wagner [28] equations. Given that the Rescorla-Wagner equations have been characterized as a general probabilistic learning mechanism [136, 137], the computational core of the model has increased biological plausibility over models that assume language-specific processing mechanisms (see [6, 138]).

The learning architecture of the ndr_a_ stands in contrast to the lexical route of the drc, cdp+, and cdp++ models, which is based on the interactive activation model of McClelland and Rumelhart [20]. In the current implementation of the cdp+ model, for instance, the contribution of the lexical route is “limited to the provision of frequency-weighted lexical phonology” [2] (p. 303). Perry et al. (2007) [2] (p. 303-304) acknowledge the problems associated with the interactive activation model in their lexical route and name the lack of learning in the lexical route of the cdp+ as one of its shortcomings.

The discriminative learning mechanism underlying the ndr_a_ also differs substantially from the connectionist networks that form the computational basis of the different versions of the triangle model (see, e.g. [4, 7, 139, 140]). As noted by Baayen et al. [138], the computational engine of the ndr_a_ is much simpler than that of connectionist models. The ndr_a_ learning networks directly map input units onto outcomes, without the intervention of one or more layers of hidden units (Note, however, that the latest version of the triangle model does not contain hidden layer units, but, instead, operates on the basis of a direct mapping between input units and outcomes [4]). The ndr_a_ is therefore more transparent than connectionist models, with activations of output units representing simple posterior probability estimates of outcomes given input units. In addition, in contrast to connectionist models the ndr_a_ does not rely on the neurobiologically implausible process of back-propagation learning.

The second major advancement of the ndr_a_ is that it uses a single lexical route architecture for both word and non-word naming. We showed that a single lexical route based on discriminative learning not only provided a good overall fit to observed naming latencies, but also captured a number of experimental results that are typically attributed to processes in the sub-lexical route. The non-linear main effects and interactions of consistency and regularity measures, for instance, are accurately captured by the ndr_a_. In addition, we showed that the ndr_a_ makes predictions for non-word naming that are highly similar to those of state-of-the-art dual-route models. Furthermore, we documented the existence of a non-word frequency effect in the classic [58] non-word reading latencies, which provides evidence for the involvement of a lexical route architecture in non-word naming.

The single-route architecture stands in contrast to the dual-route architectures of leading models of reading aloud, including both traditional dual-route models such as the drc [1, 20], cdp [9], cdp+ [2] and cdp++ [5] and the most recent versions of the triangle model (see, e.g. [4]). These models contain both a direct orthography to phonology mapping and an orthography to phonology route that is mediated by semantics. While the sub-lexical route of the cdp models has a significant contribution to the model performance (see [2]), we demonstrated that the addition of a sub-lexical discriminative learning network does not improve the performance of the ndr_a_ model.

The current implementation of the ndr_a_, however, provides a highly simplified window on reading aloud. At both the orthographic and the phonological level we make use of discrete representations at a highly restricted subset of possible grain sizes. Findings from the neuroscience literature (see, e.g. [129, 130] suggest that a more flexible system operating over multiple grain sizes may further improve the performance of the model.

In addition, the simulations reported here focused on the unimpaired language processing system. A substantial amount of work has been carried out on impaired language processing in both surface and deep dyslexia patients (see, e.g. [141, 142]). It will be interesting to see to what extent selective lesioning of the discriminative learning networks could capture the patterns of results seen in these patients. One possibility is that the pre-frontal structures and conflict resolution skills that underlie target pronunciation selection in the ndr_a_ may not be as easily accessible when the system is lesioned, possibly due to capacity limitations. Such an interpretation would fit well with the findings of Hendriks and Kolk [143], who demonstrated that the behavioral symptoms used to classify dyslexic patients into deep and surface dyslexia arise not only as a result of deficiencies in the language processing system, but also due to strategic choices in the context of the task at hand.

Furthermore, similar to the cdp+ model, the current implementation of the ndr_a_ processes mono-syllabic words only. Perry et al. [5] extended the cdp+ to allow for the processing of both mono- and bi-syllabic words, which resulted in the cdp++ model. The extension of the ndr_a_ to reading beyond the single syllable level is a further topic to explore in future research.

In its current state, however, the ndr_a_ provides a single-route alternative to state-of-the-art dual route models of response times in the reading aloud task that is based on a simple general learning algorithm and that—with a parsimonious architecture—accurately captures many of the linear and non-linear patterns in experimental word and non-word reading data.
